# Variations in foliar carbon:nitrogen and nitrogen:phosphorus ratios under global change: a meta-analysis of experimental field studies

**DOI:** 10.1038/s41598-020-68487-0

**Published:** 2020-07-22

**Authors:** Shan Xu, Jordi Sardans, Jinlong Zhang, Josep Peñuelas

**Affiliations:** 1 Guangdong Key Laboratory of Integrated Agro-Environmental Pollution Control and Management, Guangdong Institute of Eco-Environmental Science and Technology, Guangzhou, 510650 China; 2 CSIC, Global Ecology Unit CREAF-CSIC-UAB, 08913 Bellaterra, Catalonia Spain; 3 CREAF, 08913 Cerdanyola del Vallès, Catalonia Spain; 4 Flora Conservation Department, Kadoorie Farm and Botanic Garden, Tai Po, New Territories, Hong Kong SAR, China

**Keywords:** Biogeochemistry, Ecology, Climate-change ecology

## Abstract

Foliar-level stoichiometry plays an important role in ecosystem elemental cycling. Shifts in foliar ratios of carbon to nitrogen (C:N) and nitrogen to phosphorus (N:P) in response to global change can therefore have a large impact upon ecosystem function. We conducted a meta-analysis with 2,236 paired observations from 123 published studies to investigate the responses of foliar C:N and N:P ratios to experimental global change treatments, i.e. warming, increased precipitation, drought, N addition and elevated carbon dioxide concentration (eCO_2_), in field conditions. Foliar C:N and N:P ratios were neither affected by warming nor by increased precipitation. Foliar C:N ratio increased with drought and eCO_2_, and decreased with N addition. Foliar N:P ratios declined with eCO_2_, and increased under drought and N addition. Our results suggested the responses of the C:N ratio to global change were mainly related to shifts in foliar [N], whereas changes in the N:P ratio were related to the responses of both [N] and [P]. Moreover, the response magnitude of foliar N:P ratio decreased with treatment duration under increased precipitation, N addition and eCO_2_. Our findings are important for our understanding of plant nutrient dynamic and modeling of nutrient biogeochemistry under global change.

## Introduction

Concentration-based ratios of carbon to nitrogen (C:N) and nitrogen to phosphorus (N:P) are key indicators of foliar chemistry and stoichiometry, which play important roles in ecosystem energy and nutrient dynamics^1–3^. Foliar C:N ratio is closely related to litter C:N ratio and reflect leaf litter quality, which will affect the proportion of litter-derived C accumulated in soils^4^; Whereas foliar N:P ratio is an important indicator of ecosystem nitrogen (N) or phosphorus (P) limitation^5,6^. Multiple global changes, including warming, altered precipitation (increased precipitation or drought), atmospheric nitrogen (N) deposition, and elevated carbon dioxigen (CO_2_) concentrations (eCO_2_), interact to have substantial impacts upon terrestrial ecosystems and alter biogeochemical cycling^7–9^. Although some previous meta-analyses have investigated the responses of N:P ratios to global changes at the whole-plant level^10^, the responses at the foliar level have received less attention. In addition, the responses of foliar [C] and foliar C:N ratio to global change have received less attention^11–13^, and there is very little information about changes in foliar stoichiometry in response to warming and increased precipitation^14–16^. Therefore, it is urgent to investigate the responses of foliar C:N and N:P ratios to global change, including warming, changes in precipitation, N deposition and eCO_2_.

Foliar C accumulation is mainly derived from the balance between foliar photosynthesis and respiration^17–19^, which is less investigated. In contrast to foliar [C], shifts in foliar [N] and [P] in response to global change have been more extensively studied^9^. Differences in the elemental cycles of N and P are important in their responses to global change. Ecosystem N cycling is underpinned by several mechanisms of biological control (N fixation, nitrification, denitrification, nitrate-photosynthetic reduction)^20–22^, and is also influenced by atmospheric N-deposition^23^. However, ecosystem P cycling depends on fewer biological mechanisms, but has a greater dependence on the initial P content of the bedrock and the processes of soil development^9,14^, and deposition of P largely limited to areas with intense agriculture^24^. As a result, global change has the potential to alter ecosystem N-cycling by affecting various biological mechanisms. But climate change also can strongly affect P-cycling by modifying physicochemical variables that in turn can displace the equilibrium among the great array of P-chemical forms of P in soils^25^. Moreover, given the difference in solubilization between chemical forms of N and P in soil, global changes in temperature and precipitation can also alter N and P availability and uptake. Despite the potential importance of foliar stoichiometry as an indicator of change, the responses of foliar-level C:N and N:P ratios to global change drivers are still unclear^14,15^.

Previous meta-analyses have demonstrated global change impacts on C:N and N:P ratios at the whole-plant level^10,16^, by combining the results of experimental field manipulations, natural environmental gradients, and controlled greenhouse or pot experiments. Although these studies have provided important insights into plant responses to change, it is important to evaluate the results from field experiments separately, as these can differ markedly from observations along natural gradients and greenhouse or pot experiments^26,27^. To gain a better understanding of the overall responses of foliar-level C:N and N:P ratios to global change, we conducted a meta-analysis with 2,236 observations from 123 reports based on field manipulative experiments, including warming (343 observations), shifts in precipitation (158 observations for increased precipitation and 655 observations for drought), N addition (750 observations) and eCO_2_ (330 observations). Our study aimed to address the following questions: (1) How do foliar-level C:N and N:P stoichiometry respond to global change? (2) The shifts in which element concentrations ([C], [N] and [P]) have the greatest influence on foliar C:N and N:P ratios under global change? (3) Is the magnitude of shifts in foliar C:N and N:P ratios correlated with the intensity and duration of a given global change?

## Results

### Responses of foliar C:N and N:P stoichiometry to global change

**Figure 1:**
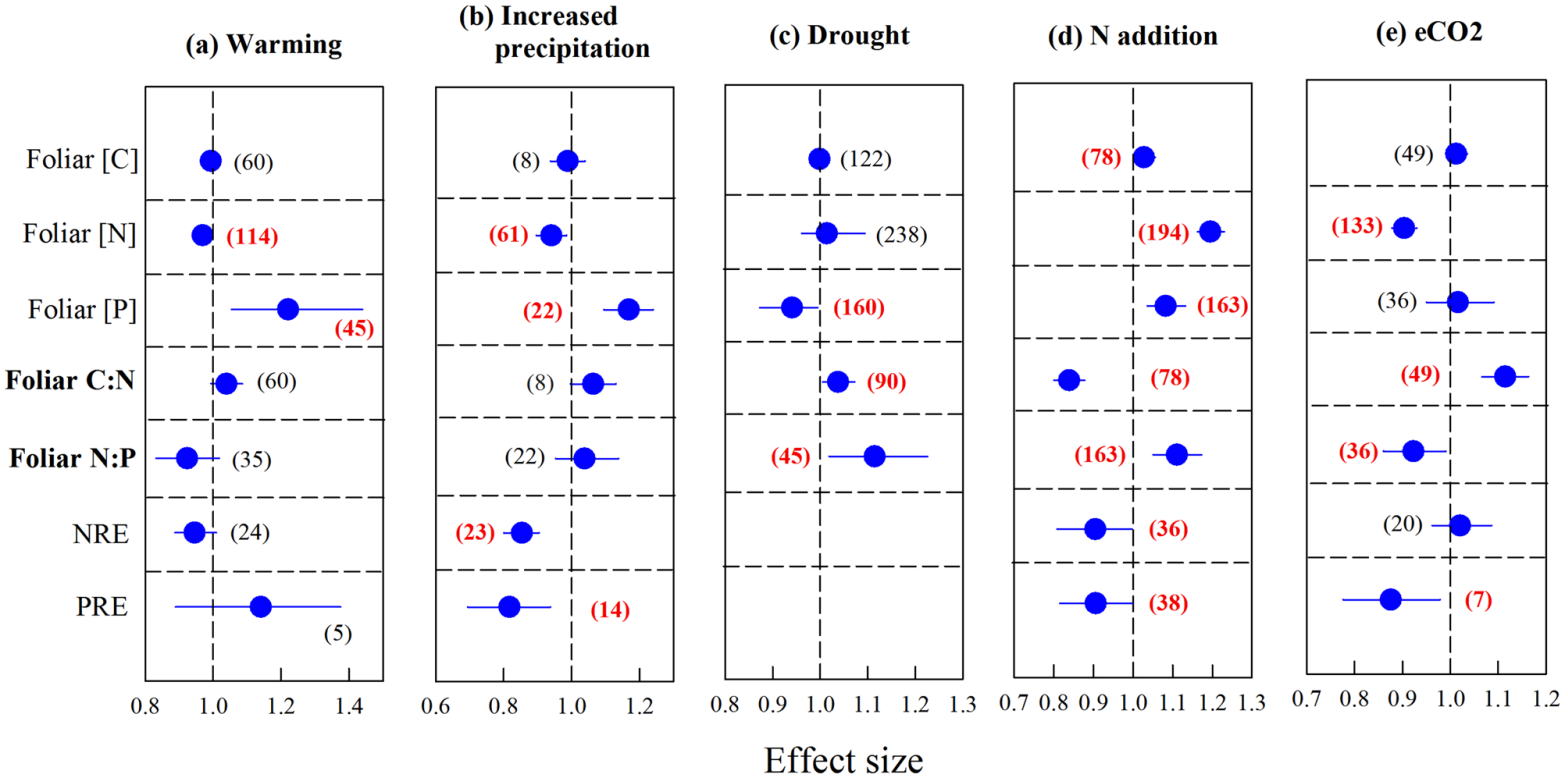
Changes in foliar carbon ([C]), nitrogen ([N]) and phosphorus ([P]) concentrations, foliar carbon to nitrogen (C:N) and nitrogen to phosphorus (N:P) ratios, and N resorption efficiency (NRE) and P resorption efficiency (PRE) under (**a**) warming, (**b**) increased precipitation, (**c**) drought, (**d**) N addition and (**e**) elevated carbon dioxide concentration (e[CO_2_]), showing effect sizes as natural log response ratios for *n* studies (numbers in parenthesis) per response variable, where an effect size of 1 (dashed line) indicates no change relative to controls. Numbers in parenthesis in bold and red color represent the significant results. The figure was performed using Sigmaplot version 11.0 (Systat Software, Inc.).

Our traditional meta-analysis demonstrates how changes in the concentrations of C, N and P have a variable influence on foliar stoichiometry under different global changes. Shifts in foliar C:N ratios largely tracked changes in foliar [N]. Foliar [C] increased slightly under N addition (n = 78, Fig. 1d), but was not affected by any other global change factors (n = 60 for warming, n = 8 for increased precipitation, n = 122 for drought, n = 49 for eCO_2_, Fig. 1a–c,e), whereas foliar [N] decreased under warming (n = 114), increased precipitation (n = 61), eCO_2_ (n = 133) and increased markedly under N addition (n = 194) (Fig. 1). Accordingly, foliar C:N ratio decreased under N addition (n = 78), but increased under eCO_2_ (n = 49) (Fig. 1). Although foliar [N] decreased under increased precipitation (n = 61), there was no change in foliar C:N ratios (n = 8) (Fig. 1b). Conversely, foliar C:N ratios increased slightly under drought (n = 90), even though neither foliar [C] nor [N] were significantly affected by drought (n = 122 and n = 238, respectively) (Fig. 1c).

However, the response ratio (LnRR) of foliar C:N ratios was positively correlated with the LnRR of foliar [C] only under increased precipitation (Fig. 3b), but negatively correlated with the LnRR of [N] under warming (Fig. 3f), increased precipitation (Fig. 3g), drought (Fig. 3h), N addition (Fig. 3i) and eCO_2_ (Fig. 3j).

For the response of foliar [P], it increased under warming (n = 45), increased precipitation (n = 22), and N addition (n = 163), but decreased under drought (n = 160) (Fig. 1). Interestingly, foliar N:P ratios were influenced by shifts in the concentrations of both nutrients, with no change under warming (n = 35) and increased precipitation (n = 22), an increase under drought (n = 45) and N addition (n = 163), and a decrease under eCO_2_ (n = 36) (Fig. 1). Thus, the LnRR of foliar N:P ratios was positively correlated with the LnRR of [N] and negatively correlated with the LnRR of [P] under warming (Fig. 4a,f), N addition (Fig. 4d,i) and eCO_2_ (Fig. 4e,j), but also negatively correlated with foliar [P] under increased precipitation (Fig. 4g) and drought (Fig. 4h).

Foliar nitrogen resorption efficiency (NRE) did not change under warming (n = 24) and eCO_2_ (n = 20), but decreased under both increased precipitation (n = 23) and N addition (n = 36) (Fig. 1). For phosphorus resorption efficiency (PRE), it did not change under warming (n = 5), but decreased under increased precipitation (n = 14), N addition (n = 38) and eCO_2_ (n = 7) (Fig. 1).

### Phylogenetic signal test and phylogenetic meta-analysis

Phylogenetic signal test have been done for the response ratios of each variable. The results showed the phylogenetic signal is significant for foliar N:P ratio under warming (K = 0.219, *P* = 0.028), foliar [N] under increased precipitation (K = 0.332, *P* = 0.023), foliar N:P ratio under N addition (K = 0.149, *P* = 0.039), and foliar C:N ratio under eCO_2_ (K = 0.642, *P* = 0.006) (Table 1). The phylogenetic signal is marginally significant for foliar [C] under warming (K = 0.121, *P* = 0.085), foliar [N] (K = 0.129, *P* = 0.067) and [P] (K = 0.132, *P* = 0.066) under N addition, foliar [N] under eCO_2_ (K = 0.167, *P* = 0.080) (Table 1).

**Table 1:** The results for the test of phylogenetic signal.

Treatment	Variable	K	*P*
Warming	Foliar [C]	**0.121**	**0.086**
Warming	Foliar [N]	0.027	0.477
Warming	Foliar [P]	0.083	0.314
Warming	Foliar CN	0.025	0.420
Warming	Foliar NP	**0.219**	**0.027**
Warming	NRE	0.116	0.794
Warming	PRE	0.388	0.894
Increased precipitation	Foliar [C]	1.108	0.103
Increased precipitation	Foliar [N]	**0.332**	**0.022**
Increased precipitation	Foliar [P]	0.491	0.113
Increased precipitation	Foliar CN	0.879	0.609
Increased precipitation	Foliar NP	0.187	0.946
Increased precipitation	NRE	0.098	0.907
Increased precipitation	PRE	0.694	0.521
N addition	Foliar [C]	0.325	0.448
N addition	Foliar [N]	**0.129**	**0.071**
N addition	Foliar [P]	**0.132**	**0.066**
N addition	Foliar CN	0.167	0.536
N addition	Foliar NP	**0.149**	**0.038**
N addition	NRE	0.117	0.593
N addition	PRE	0.218	0.913
eCO_2_	Foliar [C]	0.323	0.595
eCO_2_	Foliar [N]	**0.167**	**0.080**
eCO_2_	Foliar [P]	0.241	0.689
eCO_2_	Foliar CN	**0.642**	**0.006**
eCO_2_	Foliar NP	0.512	0.142
eCO_2_	NRE	0.162	0.256
eCO_2_	PRE	0.606	0.360

The phylogenetic signal was significant when K < 1.00 and *P* < 0.05.

*N* nitrogen, *eCO*_*2*_: elevated carbon dioxide concentration, *[C]:* carbon concentration, *[N]*: nitrogen concentration, *[P]*: phosphorus concentration, *foliar CN*: foliar carbon to nitrogen ratio, *foliar NP*: foliar nitrogen to phosphorus ratio, *NRE*: nitrogen resorption efficiency, *PRE*: phosphorus resorption efficiency.

**Figure 2:**
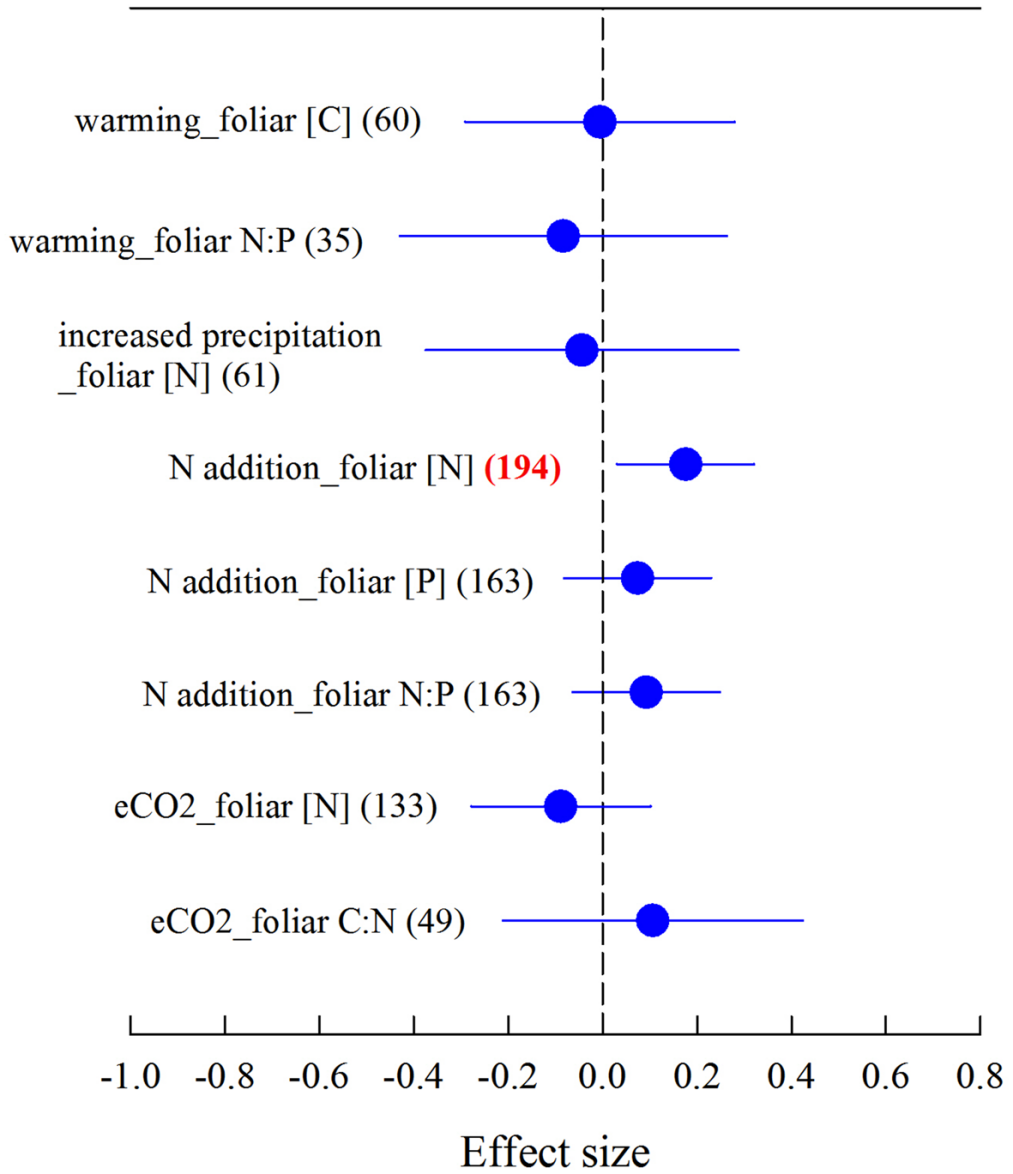
Phylogenetic meta-analysis for the variables having phylogenetic signal (we have also included those variables for which phylogenetic signal was marginally significant, i.e. *P* < 0.1), showing effect sizes as natural log response ratios for *n* studies (numbers in parenthesis) per response variable, where an effect size of 0 (dashed line) indicates no change relative to controls. Numbers in parenthesis in bold and red color represent the significant results. The figure was performed using Sigmaplot version 11.0 (Systat Software, Inc.). The figure legends follow that of Fig. 1.

The results from phylogenetic meta-analysis showed that foliar N:P ratio was not altered by warming (Fig. 2), which was consistent with the result from traditional meta-analysis (Fig. 1a). However, the unchanged foliar N:P ratio under N addition and foliar C:N ratio under eCO_2_ were inconsistent with the results from traditional meta-analysis (Figs. 1d,e, 2).

### Correlations of the response magnitudes of foliar C:N and N:P ratios with latitude, MAP and MAT

Linear regressions were used to analyze the correlations among the variations (log of response ratio: LnRR) of foliar stoichiometry with latitude, MAP, MAT under warming, increased precipitation, N addition and eCO_2_. We found relationships between the response magnitude of foliar C:N and N:P ratios with latitude, MAP, MAT were weaker under warming and eCO_2_, but stronger under increased precipitation and N addition (Table 2). Under increased precipitation, the LnRR of foliar C:N ratio was negatively related to latitude (R^2^ = 0.34, *P* = 0.036) and MAT (R^2^ = 0.34, *P* = 0.036), but positively related to MAP (R^2^ = 0.34, *P* = 0.036) (Table 2). For foliar N:P ratio, it was positively related to latitude (R^2^ = 0.19, *P* = 0.029) and MAP (R^2^ = 0.686, *P* < 0.0001), but negatively related to MAT (R^2^ = 0.458, *P* = 0.000) under increased precipitation (Table 2).

**Table 2:** Linear correlations between the variations (log of response ratio: lnRR) of foliar C:N ratio and N:P ratio with latitude, MAP, MAT under warming, increased precipitation, N addition and eCO_2_.

Treatment	Independent variables	Dependent variables	a	b	× 0	y0	*r*^2^	*P*
Warming	Latitude	C:N	–	–	–	–	–	–
		N:P	–	–	–	–	–	–
	MAP	C:N	–	–	–	–	–	–
		N:P	–	–	–	–	–	–
	MAT	C:N	–	–	–	–	–	–
		N:P	–	–	–	–	–	–
Increased precipitation	Latitude	C:N	− 0.214	9.465	–	–	0.340	**0.036**
		N:P	0.085	− 3.634	–	–	0.190	**0.029**
	MAP	C:N	0.001	− 0.159	–	–	0.340	**0.036**
		N:P	0.726	78.638	267.856	− 0.351	0.686	**< 0.0001**
	MAT	C:N	− 0.02	0.199	–	–	0.340	**0.036**
		N:P	− 0.042	0.309	–	–	0.458	**0.000**
N addition	Latitude	C:N	0.014	− 0.725	–	–	0.173	**< 0.0001**
		N:P	–	–	–	–	–	–
	MAP	C:N	− 0.001	0.107	–	–	0.115	**0.002**
		N:P	–	–	–	–	–	–
	MAT	C:N	–	–	–	–	–	–
		N:P	–	–	–	–	–	–
eCO_2_	Latitude	C:N	–	–	–	–	–	–
		N:P	–	–	–	–	–	–
	MAP	C:N	–	–	–	–	–	–
		N:P	–	–	–	–	–	–
	MAT	C:N	–	–	–	–	–	–
		N:P	–	–	–	–	–	–

The correlations were significant when *P* < 0.05.

*MAP*: mean annual precipitation (mm), *MAT*: mean annual temperature (°C), *[CO*_*2*_*]*: carbon dioxide concentration, *N*: nitrogen, *C:N*: foliar carbon to nitrogen ratio, *N:P*: foliar nitrogen to phosphorus ratio.

### Correlations of the response magnitudes of foliar C:N and N:P with treatment levels and durations

We further investigated how the level of different global change treatments affected the magnitude of the response in foliar C:N and N:P ratios (Fig. 5). The LnRR of N:P ratios were not related to treatment level for any of the studied global change drivers (Fig. 5e–h) and treatment level had no influence on the LnRR of foliar C:N to increasing temperature (Fig. 5a) or precipitation (Fig. 5b). However, the LnRR of foliar C:N ratios decreased with increasing rate of N addition (R^2^ = 0.17, *P* = 0.0002, Fig. 5c), and there was a bimodal relationship between the LnRR of foliar C:N and eCO_2_, whereby the increase in foliar C:N ratios was greatest at intermediate [CO_2_] treatment levels (R^2^ = 0.30, *P* = 0.004, Fig. 5d).

We also assessed how the treatment duration affected the magnitude of the response in foliar C:N and N:P ratios (Fig. 6). We found the LnRR of foliar N:P ratio decreased with treatment duration under increased precipitation (R^2^ = 0.25, *P* = 0.01), N addition (R^2^ = 0.20, *P* < 0.0001) and eCO_2_ (R^2^ = 0.12, *P* = 0.04) (Fig. 6f–h).

## Discussion

In this study, we investigated how foliar C:N and N:P ratios respond to global change, including warming, increased precipitation, drought, N addition and eCO_2_ by a global meta-analysis. We demonstrate that the response of foliar C:N ratio to global change was largely explained by shifts in foliar [N], whereas the response of foliar N:P ratio was influenced by shifts in both [N] and [P]. When doing linear regressions, we found the response magnitudes of foliar C:N and N:P ratios were largely affected by latitude, MAP and MAT under increased precipitation treatment. When doing multiple regressions, we found the effects of "latitude + MAP + MAT + branch length + treatment duration + treatment level" on the LnRRs of foliar C:N and N:P ratios were significant under N addition treatment. In contrast to previous meta-analyses^10,14^, our study only included the results of field manipulative experiments, which are subject to much greater environmental variability than pot and greenhouse studies. Further, distinct responses of foliar nutrients to global change, compared to responses at the whole-plant level, can reflect important shifts in nutrient allocation among plant parts.

### Responses of foliar C:N ratio were mainly related to shifts in foliar [N] under global change

**Figure 3:**
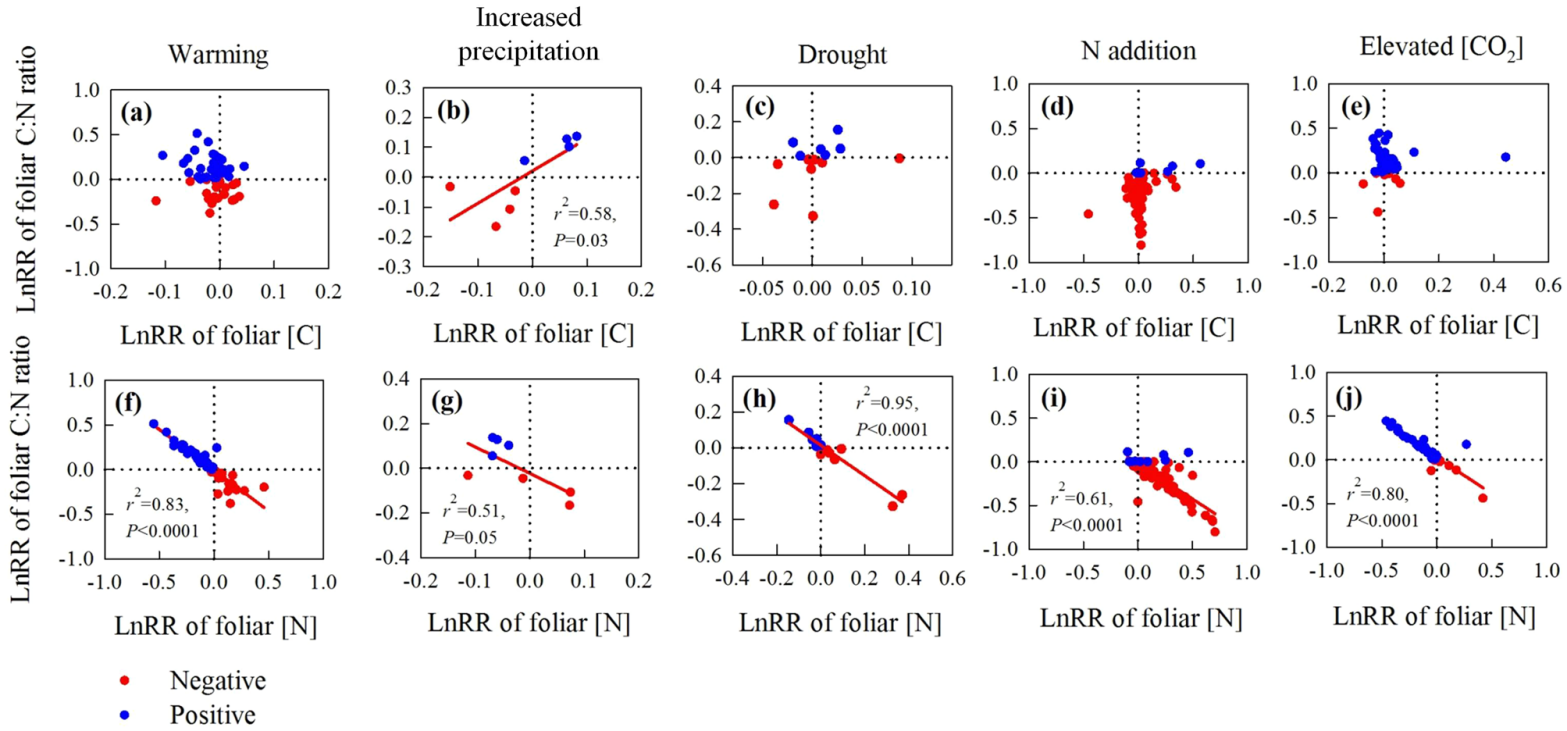
Correlations between the natural log response ratio (lnRR) of the foliar C:N ratio and [C] under (**a**) warming, (**b**) increased precipitation, (**c**) drought, (**d**) N addition and (**e**) eCO_2_ and the relationship between the lnRR of the foliar C:N ratio and [N] under (**f**) warming, (**g**) increased precipitation, (**h**) drought, (**i**) N addition and (**j**) eCO_2_. See Fig. 2 for definitions of the abbreviations. Red and blue dots represent negative and positive responses of the C:N ratio, respectively. The figure was performed using Sigmaplot version 11.0 (Systat Software, Inc.).

Our results showed changes in foliar C:N ratios were mainly explained by shifts in foliar [N] (Figs. 1, 3), whereas foliar [C] was generally unaffected by global change treatments (Fig. 1). This is perhaps not surprising because increased CO_2_ uptake by plants via photosynthesis is largely constrained by foliar [N]^28^ and foliar [C] reflects the balance of foliar-level photosynthesis and respiration^17–19^. Nonetheless, we detected a small increase in foliar [C] in response to N addition (Fig. 1d), which could indicate greater investment in structural C or chemical defenses against herbivory with increasing foliar [N]^29,30^. Indeed, the large increase in foliar [N] under N addition resulted in lower foliar C:N ratios despite increased foliar [C] (Fig. 1d), which would make leaves more palatable to herbivores^30^. Lower C:N ratios under N addition were also mediated by increased foliar [N] (Figs. 1d, 3d, i), consistent with studies along fertility gradients^13^, and previous meta analyses^10^ in which a negative correlation between the C:N ratio and [N] in foliar tissues were observed.

There is very little information about changes in foliar stoichiometry in response to warming and increased precipitation^14–16^. Interestingly, our results showed foliar C:N ratios were not affected by warming or increased precipitation (Figs. 1a,b), but increased under eCO_2_ (Fig. 1e). This increase in foliar C:N ratio was largely due to a sharp decrease in foliar [N] under eCO_2_ (Figs. 1e, 3j), which is likely attributed to a dilution effect and progressive N limitation^9,30,31^, although we found no effect of eCO_2_ on NRE (Fig. 1e). We found that the foliar C:N ratio increased under drought (Fig. 1c) even though the decline in [N] was not significant (Fig. 1c). Increased foliar C:N ratios under drought have been widely reported^29^, and are correlated with increases in C-rich compounds associated with morphological, metabolic and physiological defenses against water deficit^32,33^, and with lower N-uptake capacity under drought^31^. Our results showed that the increase in the C:N ratio is mostly due to a decrease in [N] and not due to changes in [C] is consistent with a recent meta-analysis showing that drought treatments reduce plant N-uptake capacity^31^.

### Responses of foliar N:P ratio were related to altered [N] and [P] under global change

**Figure 4:**
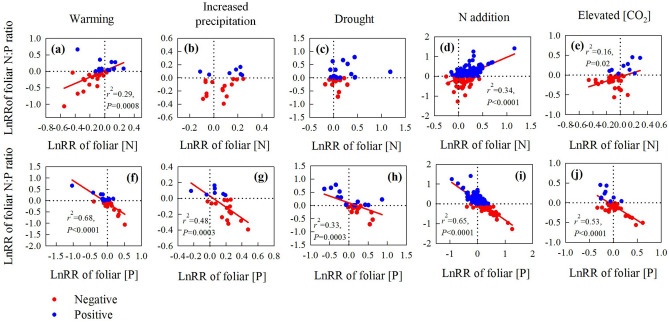
Correlations between the natural log response ratio (lnRR) of foliar N:P ratios and foliar [N] under (**a**) warming, (**b**) increased precipitation, (**c**) drought, (**d**) N addition and (**e**) eCO_2_ and the relationship between the lnRR of the foliar N:P ratio and [P] under (**f**) warming, (**g**) increased precipitation, (**h**) drought, (**i**) N addition and (**j**) eCO_2_. See Fig. 2 for definitions of the abbreviations. Red and blue dots represent negative and positive responses of the N:P ratio, respectively. The figure was performed using Sigmaplot version 11.0 (Systat Software, Inc.).

Although foliar [N] and [P] showed distinct and variable responses to global change (Fig. 1), shifts in the N:P ratio were consistently positively correlated with the response of foliar [N] and negatively related to the response of foliar [P] under all global change factors (Fig. 4, Table S3). Hence, shifts in the foliar N:P ratio in response to global change are determined by contrasting changes in both nutrients, even though the individual responses of [N] and [P] were not always statistically significant. Foliar N:P ratios were not altered by warming or increased precipitation (Fig. 1a, b), which is due to the reverse responses of foliar [N] and foliar [P] under warming and increased precipitation (Fig. 1a, b).

Nonetheless, the increased foliar N:P ratios under drought (Fig. 1c) were explained by a decrease in foliar [P]; the analyses of the complete data set (n = 238 studies for [N] and n = 160 studies for [P]) indicated that drought significantly decreased [P] but had no significant effect on [N] (Fig. 1c). This finding was corroborated by analysis of a subset of the data (n = 35), including only studies that reported results for both nutrients, in which the response of foliar N:P ratios to drought was negatively correlated with the response of foliar [P] (Fig. 4h). Although studies along precipitation gradients have also demonstrated changes in foliar N:P^26^, this contrasts with previous meta-analyses^10,14^ in which no change in foliar N:P ratios was observed in response to drought. It is conceivable that we detected the drought effect on foliar [P] and N:P ratios in our study because of the inherent differences in the solubility and mobility of both elements in the field, which will have a lesser influence in the small-scale studies included in other meta-analyses. Decreased foliar [P] under drought is consistent with the reduced capacity of plants to take up P and the lower foliar [P] in more arid environments^34^. The diffusivity of P in the soil is more sensitive to soil moisture than that of N and hence plant P-uptake will be more strongly limited by water availability than N-uptake^9,14,35^. As a result, increasing aridity may eventually decouple the N and P cycle with reduced [N] and increased [P] in the soil^36^.

Both foliar [N] and [P] increased under N addition, but the magnitude of the increase was significantly higher for [N] than [P] (Fig. 1d). Accordingly, the increase in the N:P ratio under N addition was largely attributed to increased foliar [N] (Fig. 1d), consistent with a previous meta-analysis at the whole-plant level^37^, even though N addition decreased NRE (Fig. 1d). The observed increase in foliar [P] under N addition was probably related with changes in PRE, because PRE was decreased by N addition (Fig. 1d), and was marginally correlated with changes in foliar [P] (Fig. S10b, R^2^ = 0.25, *P* = 0.07). It is conceivable that increased [P] with N-addition is due to an increase in soil P mineralization as a result of N-investment in phosphatase production^38,39^. It is noteworthy that the changes in [P] we observed in field manipulation studies were inconsistent with the results from previous global meta-analyses, which found that N fertilization did not affect foliar [P] and decreased root [P]^15^. Taken together with our results, this suggests that plants respond to N enrichment by increasing P allocation to leaves and decreasing the allocation of P to roots. The increased foliar N:P ratio under N addition nevertheless implies that P limitation will become more severe in areas with continuing substantial atmospheric N deposition.

The foliar N:P ratio decreased under elevated atmospheric [CO_2_] (Fig. 1e) and our analyses of the complete data set (n = 133 studies for [N] and n = 36 studies for [P]) attributed this to decreased [N]. However, our analyses of the subset of data containing information for both nutrients in the same studies (n = 36) showed that the response of foliar N:P ratios was both positively correlated with the response of foliar [N], but negatively related to the response of foliar [P] (Fig. 4e,j). This suggests that although the decrease in foliar N:P can mainly be attributed to decreased foliar [N], changes in the concentrations of both nutrients played a role in determining shifts in N:P ratios under eCO_2_. The greater shifts in foliar [N] relative to [P] under eCO_2_ could be due to the indirect effect of reduced water uptake and stomata conductance^40^, which should affect more N uptake more than P uptake due to the higher solubility of N^9,41^.

### How do global change treatment intensities and duration affect the response of foliar C:N or N:P ratios?

**Figure 5:**
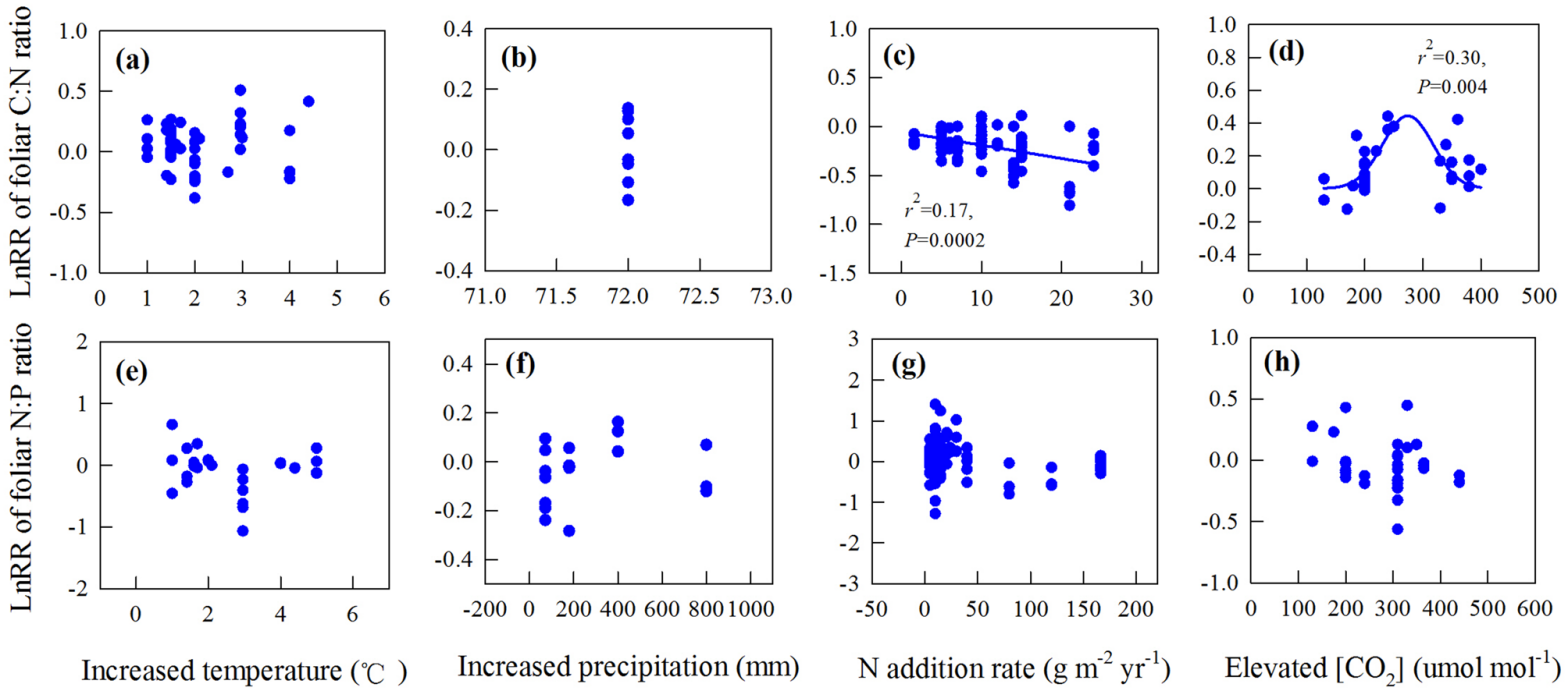
Correlations between the natural log response ratio (lnRR) of the foliar C:N ratio with (**a**) increased temperature (°C), (**b**) altered precipitation (mm), (**c**) N-addition rate (g m^−2^ years^−1^) and (**d**) eCO_2_ (µmol mol^−1^) and between lnRR of the N:P ratio with (**e**) increased temperature (°C), (**f**) altered precipitation (mm), (**g**) N-addition rate (g m^−2^ years^−1^) and (**h**) eCO_2_ (µmol mol^−1^). See Figs. 2 and 3 for the definitions of the abbreviations. The figure was performed using Sigmaplot version 11.0 (Systat Software, Inc.).

Characterizing how the magnitude of global change will affect foliar C:N and N:P ratios is important for predicting future biogeochemical cycling under different global change scenarios. Unexpectedly, both the C:N and N:P ratios were resistant to global warming in our study, even though warming levels were as high as 5 °C (Fig. 5a,e), and although there were trends towards declining foliar N:P ratios with the level of N addition and eCO_2_, these were not significant. By contrast, the response magnitude of foliar C:N ratio declined with N addition rate, especially at loads above *c*. 10 g N m^−2^ y^−1^ (Fig. 5c). The results from multiple regressions also showed N addition rate significantly affected the response of foliar C:N ratio to N addition (Table S3). Interestingly, the increase in foliar C:N ratios with CO_2_ enrichment was highest at intermediate eCO_2_ levels (Fig. 5d), which indicates plant physiological constraints to responses in foliar stoichiometry above *c*. 350 µmol mol^−1^ CO_2._ For increased precipitation, although the response magnitude of foliar C:N and N:P ratios were not affected by the level of increased precipitation (Fig. 5b,f), the LnRRs of foliar C:N ratio and foliar N:P ratio were positively related to MAP (Table 2). These results suggested the responses of foliar stoichiometry to increased precipitation were specific among sites with different levels of MAP, which results in the overall non-significant response of foliar stoichiometry to increased precipitation (Fig. 1b).

**Figure 6:**
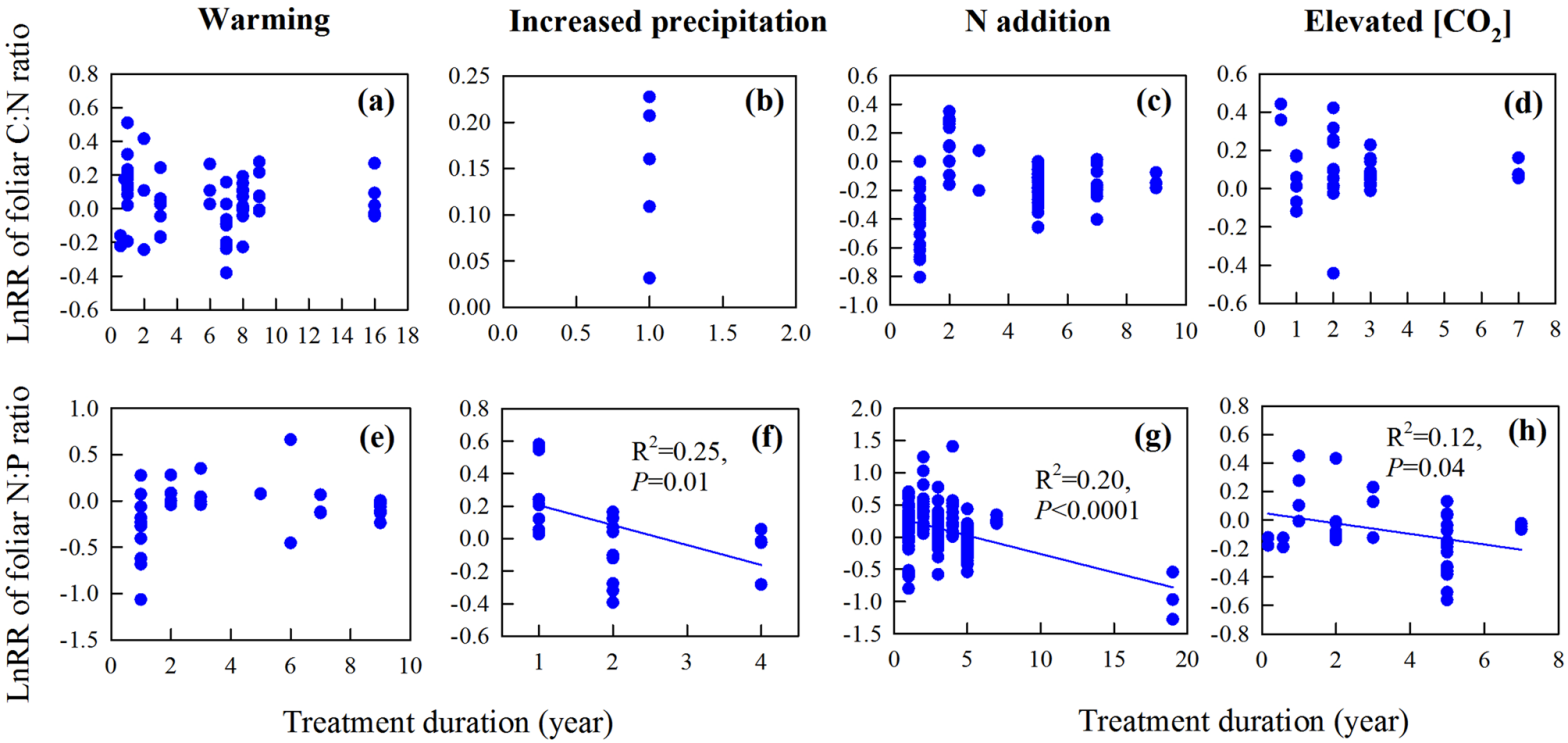
Correlations between the response ratios of foliar C:N ratio, foliar N:P ratio and the treatment duration of warming, changed precipitation, N addition and eCO_2_. *N*: nitrogen, *C:N ratio*: carbon to nitrogen ratio, *N:P ratio*: nitrogen to phosphorus ratio, *[CO*_*2*_*]*: carbon dioxide concentration, *LnRR*: natural log of the response ratio. The correlation was significant when *P* < 0.05. The figure was performed using Sigmaplot version 11.0 (Systat Software, Inc.).

Our results suggested that treatment duration did not affect the response magnitudes of foliar C:N ratio to global change (Fig. 6a–d). But the LnRR of foliar N:P ratio decreased with treatment duration under increased precipitation, N addition and eCO_2_ (Fig. 6f–h). This was probably attributed to the correlation between foliar [P] and treatment duration under increased precipitation, N addition and eCO_2_ (Fig. S13j–l). This also confirmed ecosystem P cycling is more conserved compared to ecosystem N cycling^9,14^, thus can be significantly affected by treatment duration under global change. These results are important for us to predict spatiotemporal variations in foliar stoichiometry under future global change.

## Final remarks and conclusions

Our results suggested the effects of the studied global change drivers on foliar C:N ratios were due mainly to changes in [N], but the shifts in N:P ratio were more complex. Warming enhanced foliar [P] and decreased foliar [N] with a resulting statistically not-significant trend to decreased foliar N:P ratio. Further studies are needed to separate the effects of warming in wetter and drier sites when more data will be available, since warming can have opposite effects in wetter and drier sites given its impact enhancing aridity in drier sites. Increased precipitation and drought had greater effects on foliar [P] than on foliar [N], and increased precipitation enhanced foliar [P] whereas drought reduced foliar [P]. These results indicate that availability of soil water has a great impact on plant uptake capacity of P than N given that soil P is often immobile compared to soil N. N addition increased foliar N:P due to the larger increases in [N] than in [P], and eCO_2_ reduced foliar N:P due to the decrease in [N] but not in [P].

In contrast to previous meta-analyses, by limiting our study to in situ experiments, we demonstrate that shifts in foliar N:P ratios in response to global changes can be attributed to changes in the foliar concentrations of both N and P. In addition, their responses of to global change were independent of their phylogenetic signal. We propose that greenhouse or laboratory studies may limit the influence of the distinct motilities of N and P in the soil and therefore plant nutrient uptake in response to global changes in small-scale experiments may not reflect the true responses of plants under field conditions. Our findings are important for our understanding of plant nutrient dynamic and modeling of nutrient biogeochemistry under global change.

## Material and methods

### Data selection

A comprehensive search of relevant peer-reviewed articles and dissertations published from 1997 to 2017 was conducted using the databases of the Web of Science, ProQuest and China National Knowledge Infrastructure (CNKI). We also cross-checked the references of selected articles to identify other potential book chapters and peer-reviewed reports using combinations of the following keywords: carbon, concentration, C:N, leaf, CO_2_, leaf, needle, nitrogen, N:P, phosphorus, plant, ratio, stoichiometric, stoichiometry, warming, increased precipitation, drought and N deposition/addition. We extracted data for foliar [C], [N], [P] and N- and P-resorption efficiencies of different plant species. These plant species covered multiple life forms including crop species, grasses, mosses, shrubs and trees. When data from multiple years were given for the same study, we only selected data from the last year to avoid temporal pseudo-replication^42^. We also recorded foliar C:N and N:P ratios or calculated them using the ratios of foliar [C], [N] and [P] (i.e. foliar C:N = foliar [C]/[N] and foliar N:P = foliar [N]/[P]) when only foliar [C], [N] and [P] given and there was no data for C:N and N:P in the literature. Numerical values were extracted from graphically presented data by digitizing the figures using Engauge Digitizer (Free Software Foundation, Inc., Boston, USA). The experimental sites included in our study are shown in Fig. S1, which was generated in R version 3.4.2 (R Core Team, 2017, https://www.R-project.org/) using ggplot2^43^ and the R packages ggsn^44^ and legendMap^45^.

### Meta-analysis

The data were analyzed as described by Hedges et al.^46^. The effect sizes for warming, altered precipitation, N addition and eCO_2_ for each observation were represented by log response ratios (RR): $\ln RR = \ln \left( {\overline{{X_{t} }} /\overline{{X_{c} }} } \right)$, where $\overline{{X_{c} }}$ is the control mean, and $\overline{{X_{t} }}$ is the treatment mean. The publication bias was estimated by the Gaussian function (Figs. S2–S6 in the Supporting Information, which was generated by R version 3.3.3 (R Core Team, 2017, https://www.R-project.org/), and the frequency distributions of all the RR values of the target variables followed a normal distribution, indicating an absence of publication bias in our study. The average RR for each global change was calculated using the mixed model of the meta-analytical program METAWIN (Sinauer Associates, Inc. Sunderland, USA) and the variances of the mean effect sizes were calculated using resampling techniques^47^. If the lower bound of the 95% confidence interval (CI) of a given RR was > 1, then the response was significantly positive at *P* < 0.05. If the upper bound of the 95% CI of RR was < 1, then the response was significantly negative at *P* < 0.05. A subgroup analysis was conducted for each parameter to identify differences in effect sizes among different life forms. Total heterogeneity (*Q*_*T*_) was partitioned into within-group (*Q*_*W*_) and between-group (*Q*_*B*_) heterogeneities, whereby a significant *Q*_*B*_ indicates a different RR among groups^46^ and group means were considered significantly different if their 95% CIs did not overlap. A full description of the meta-analysis is provided in SI Appendix B. Figure 1 was performed using Sigmaplot version 11.0 (Systat Software, Inc.).

### The phylogenetic information of plant species and phylogenetic meta-analysis

We created the phylogenetic tree by "phytools" package (phylogenetic tools for comparative biology—and other things)^48^ in R version 3.3.3 (R Core Team, 2017, https://www.R-project.org/) based on the Scientific names of the species given in the literature and got the branch length (million years) of each species, which represents the phylogenetic information, i.e. evolutionary history, of each species. The branch length of the plant species can be found in the SI Appendix B.

We used the function "phylosignal" in R to test whether there is phylogenetic signal for the response ratio of each variable in R version 3.3.3 (R Core Team, 2017, https://www.R-project.org/). When a phylogenetic signal exists in the corresponding response ratio, then we did the meta analysis including phylogeny, i.e. phylogenetic meta-analyses. The detailed methods for phylogenetic meta-analyses referred to the methods in the papers Adams^49^ and Yan et al.^50^. We did phylogenetic meta-analyses in R version 3.3.3 (R Core Team, 2017, https://www.R-project.org/). The R code was detailed in SI Appendix C.

### Regression analyses

Linear and nonlinear correlations were used to analyze the relationships among the variation (log of response ratio: lnRR) of each variable and latitude, mean annual precipitation (MAP), mean annual temperature (MAT) under warming, increased precipitation, N addition and eCO_2_. To test whether the phylogenetic information for specific species affect the response of foliar stoichiometry to global change, we analyzed the correlations between foliar stoichiometry with the branch length (million years) away from the phylogenetic tree of each species. To identify whether changes in foliar stoichiometry were best explained by changes in foliar [C], [N] or [P], we also analyzed the relationships between RR of the C:N ratio and foliar [C] or [N], as well as between RR of the N:P ratio and foliar [N] or [P] using Pearson’s correlations. The treatment levels for increased temperature (°C), altered precipitation (mm), rate of N addition (g m^−2^ years^−1^) and eCO_2_ (µmol mol^−1^) were available in 63% CO_2_ enrichment treatment, 88% N addition treatment, 80% warming treatment, 89% increased precipitation treatment. Therefore, we used linear regression to assess the influence of treatment levels on the RR of foliar C:N or N:P ratios. We also used linear regression to assess the influence of treatment duration (year) on the RR of foliar C:N and N:P ratios. All linear analyses were performed using R version 3.3.3 (R Core Team, 2017, https://www.R-project.org/). Figures S8–S13 was performed using Sigmaplot version 11.0 (Systat Software, Inc.).

Multiple regressions were used to analyze the effects of "latitude + MAP + MAT + branch length + treatment duration + treatment level" on the LnRR of foliar C:N and N:P ratios when all the information are given simultaneously. The multiple regression analyses were performed using the R code "lm" in R version 3.3.3 (R Core Team, 2017, https://www.R-project.org/). The results are detailed in Table S3.

## Supplementary information

Supplementary Information 1 (DOCX 2988 kb)

Supplementary Information 2 (XLSX 187 kb)

Supplementary Information 3 (DOCX 29 kb)

## Data Availability

The data set of the peer-reviewed publications will be accessible after acceptance.

## References

[1] He JS (2006). Stoichiometry and large-scale patterns of leaf C and nitrogen in the grassland biomes of China. Oecologia.

[2] Townsend AR, Cleveland CC, Asner GP, Bustamante MMC (2007). Controls over foliar N:P ratios in tropical rain forests. Ecology.

[3] Hättenschwiler S (2008). High variation in foliage and leaf litter chemistry among 45 tree species of a neotropical rainforest community. New Phytol..

[4] Zhou G (2019). Climate and litter C/N ratio constrain soil organic carbon accumulation. Nat. Sci. Rev..

[5] Vitousek PM, Porder S, Houlton BZ, Chadwick OA (2010). Terrestrial phosphorus limitation: Mechanisms, implications, and nitrogen–phosphorus interactions. Ecol. Appl..

[6] Xu S (2017). Different spatial patterns of nitrogen and phosphorus resorption efficiencies in China’s forests. Sci. Rep..

[7] Elser JJ (2010). Biological stoichiometry of plant production: Metabolism, scaling and ecological response to global change. New Phytol..

[8] Sardans J, Rivas-Ubach A, Peñuelas J (2011). The elemental stoichiometry of aquatic and terrestrial ecosystems and its relationships with organismic lifestyle and ecosystem structure and function: A review and perspectives. Biogeochemistry.

[9] Peñuelas J (2013). Human-induced nitrogen–phosphorus imbalances alter natural and managed ecosystems across the globe. Nat. commun..

[10] Yuan ZY, Chen HYH (2015). Decoupling of nitrogen and phosphorus in terrestrial plants associated with global changes. Nat. Clim. Change.

[11] Lindroth RL (2001). Consequences of elevated C dioxide and ozone for foliar chemical composition and dynamics in trembling aspen (*Populus tremuloides*) and paper birch (*Betula papyrifera*). Environ. Pollut..

[12] Liu L, King JS, Giardina CP (2005). Effects of elevated concentrations of atmospheric CO_2_ and tropospheric O_3_ on leaf litter production and chemistry in trembling aspen and paper birch communities. Tree Physiol..

[13] Luo W (2017). C and nitrogen allocation shifts in plants and soils along aridity and fertility gradients in grasslands of China. Ecol. Evol..

[14] Sardans J, Rivas-Ubach A, Peñuelas J (2012). The elemental stoichiometry of aquatic and terrestrial ecosystems and its relationships with organismic lifestyle and ecosystem structure and function: A review and perspectives. Biogeochemistry.

[15] Sardans J (2017). Changes in nutrient concentrations of leaves and roots in response to global change factors. Glob. Change Biol..

[16] Yue K (2017). Influence of multiple global change drivers on terrestrial carbon storage: additive effects are common. Ecol. Lett..

[17] Paul MJ, Pellny TK (2003). Carbon metabolite feedback regulation of leaf photosynthesis and development. J. Exp. Bot..

[18] Flexas J (2006). Keeping a positive C balance under adverse conditions: Responses of photosynthesis and respiration to water stress. Physiol. Plantarum.

[19] Chen S, Lin G, Huang J, Jenerette GD (2009). Dependence of C sequestration on the differential responses of ecosystem photosynthesis and respiration to rain pulses in a semiarid steppe. Glob. Change Biol..

[20] Booth MS, Stark JM, Rastetter E (2005). Controls on nitrogen cycling in terrestrial ecosystems: A synthetic analysis of literature data. Ecol. Monogr..

[21] Zheng MH, Zhou ZH, Luo YQ, Zhao P, Mo JM (2019). Global pattern and controls of biological nitrogen fixation under nutrient enrichment: A meta-analysis. Glob. Change Biol..

[22] Barnard R, Leadley PW (2005). Global change, nitrification, and denitrification: A review. Glob. Biogeochem. Cycle..

[23] Lu M (2011). Responses of ecosystem nitrogen cycle to nitrogen addition: A meta-analysis. New Phytol..

[24] King KW (2014). Phosphorus transport in agricultural subsurface drainage: A review. J. Environ. Qual..

[25] Hou E (2018). Effects of climate on soil phosphorus cycle and availability in natural terrestrial ecosystems. Glob. Change Biol..

[26] Yuan ZY (2017). Experimental and observational studies find contrasting responses of soil nutrients to climate change. eLife.

[27] Dunne JA, Saleska SR, Fischer ML, Harte J (2004). Integrating experimental and gradient methods in ecological climate change research. Ecology.

[28] Kattge J, Knorr W, Raddatz T, Wirth C (2009). Quantifying photosynthetic capacity and its relationship to leaf nitrogen content for global-scale terrestrial biosphere models. Glob. Change Biol..

[29] Invers O, Kraemer GP, Pérez M, Romero J (2004). Effects of nitrogen addition on nitrogen metabolism and carbon reserves in the temperate seagrass *Posidonia oceanica*. J. Exp. Mar. Biol. Ecol..

[30] Throop HL, Lerdau MT (2004). Effects of nitrogen deposition on insect herbivory: Implications for community and ecosystem processes. Ecosystems.

[31] Homyak PE (2017). Effect of drought manipulation on soil nitrogen cycling: A meta-analysis. J. Geophys. Res.-Biogeosci..

[32] Tharayil N (2011). Changes in the structural composition and reactivity of *Acer rubrum* leaf litter tannins exposed to warming and altered precipitation: Climatic stress-induced tannins are more reactive. New Phytol..

[33] Tattini M (2015). Isoprenoids and phenylpropanoids are part of the antioxidant defense orchestrated daily by drought-stressed Platanusxacerifolia plants during Mediterranean summers. New Phytol..

[34] Bertiller MB, Sain CL, Carrera AL, Vargas DN (2005). Patterns of nitrogen and phosphorus conservation in dominant perennial grasses and shrubs across an aridity gradient in Patagonia Argentina. J. Arid Environ..

[35] Lambers H, Chapin FS, Pons TL (2008). Plant Physiological Ecology.

[36] Delgado-Baquerizo M (2013). Decoupling of soil nutrient cycles as a function of aridity in global drylands. Nature.

[37] Yuan ZY, Chen HYH (2015). Negative effects of fertilization on plant nutrient resorption. Ecology.

[38] Keeler BL, Hobbie SE, Kellogg LE (2008). Effects of long-term nitrogen addition on microbial enzyme activity in eight forested and grassland sites: Implications for litter and soil organic matter decomposition. Ecosystems.

[39] Marklein AR, Houlton BZ (2012). Nitrogen inputs accelerate phosphorus cycling rates across a wide variety of terrestrial ecosystems. New Phytol..

[40] Staddon PL, Gregersen R, Jakobsen I (2004). The response of two Glomus mycorrhizal fungi and a fine endophyte to elevated atmospheric CO_2_, soil warming and drought. Glob. Change Biol..

[41] He M, Dijsktra FA (2014). Drought effect on plant nitrogen and phophorus: A meta-analysis. New Phytol..

[42] Koricheva J, Gurevitch J (2014). Uses and misuses of meta-analysis in plant ecology. J. Ecol..

[43] Wickham H (2009). ggplot2: Elegant graphics for data analysis.

[44] Baquero, O.S. ggsn: North symbols and scale bars for maps created with ‘ggplot2’ or ‘ggmap’. https://CRAN.R-project.org/package=ggsn (2017).

[45] Gallic, E. legendMap: North arrow and scale bar for ggplot2 graphics. R package version 1.0 (2016).

[46] Hedges LV, Gurevitch J, Curtis PS (1999). The meta-analysis of response ratios in experimental ecology. Ecology.

[47] Adams DC, Gurevitch J, Rosenberg MS (1997). Resampling tests for meta-analysis of ecological data. Ecology.

[48] Revell LJ (2012). phytools: An R package for phylogenetic comparative biology (and other things). Methods Ecol. Evol..

[49] Adams DC (2008). Phylogenetic meta-analysis. Evolution.

[50] Yan K (2018). Caution is needed in quantifying terrestrial biomass responses to elevated temperature: Meta-analyses of field-based experimental warming across China. Forests.

