# Supplementary material for: Variations in foliar carbon:nitrogen and nitrogen:phosphorus ratios under global change: a meta-analysis of experimental field studies
Source: Sci Rep. 2020 Jul 22;10:12156. doi: 10.1038/s41598-020-68487-0 (PMC7376191; doi:10.1038/s41598-020-68487-0)
Supplement: Supplementary file 1 — Supplementary Information 1 (DOCX 2988 kb) [file 41598_2020_68487_MOESM1_ESM.docx]

*Supplementary materials*

**Variations in foliar carbon:nitrogen and nitrogen:phosphorus ratios under global change: a meta-analysis of experimental field studies**

**Running head:** Foliar C:N and N:P ratios under global change

**Authors:** Shan Xu^1*^, Jordi Sardans^2,3^, Jinlong Zhang^4^, Josep Peñuelas^2,3^

**Author affiliations:**

^1^Guangdong Key Laboratory of Integrated Agro-environmental Pollution Control and Management, Guangdong Institute of Eco-environmental Science & Technology, Guangzhou, 510650, China

^2^CSIC, Global Ecology Unit CREAF- CSIC-UAB. 08913 Bellaterra. Catalonia. Spain

^3^CREAF. 08913 Cerdanyola del Vallès. Catalonia. Spain

^4^Flora Conservation Department, Kadoorie Farm and Botanic Garden, Tai Po, New Territories, Hong Kong SAR, China

**Table S1** Linear and nonlinear correlations among the variation (log of response ratio: lnRR) of each variable and latitude, MAP, MAT under warming, elevated CO_2_, increased precipitation and N addition. MAP: mean annual precipitation; MAT: mean annual temperature; CO_2_: carbon dioxide; N: nitrogen; [C]: lnRR of foliar [C]; [N]: lnRR of foliar [N]; [P]: lnRR of foliar [P]; NRE: nitrogen resorption efficiency; PRE: phosphorus resorption efficiency. The correlations were significant when *P*<0.05.

| Treatment | Independent | Dependent | a | b | x0 | y0 | *r*^2^ | *P* |
| --- | --- | --- | --- | --- | --- | --- | --- | --- |
| **Warming** | Latitude | [C] | - | - | - | - | - | - |
|  |  | [N] | - | - | - | - | - | - |
|  |  | [P] | - | - | - | - | - | - |
|  |  | NRE | -0.004 | 0.211 | - | - | 0.468 | **0.014** |
|  |  | PRE | - | - | - | - | - | - |
|  | MAP | [C] | - | - | - | - | - | - |
|  |  | [N] | - | - | - | - | - | - |
|  |  | [P] | -0.001 | 0.789 | - | - | 0.128 | **0.016** |
|  |  | NRE | - | - | - | - | - | - |
|  |  | PRE | - | - | - | - | - | - |
|  | MAT | [C] | - | - | - | - | - | - |
|  |  | [N] | - | - | - | - | - | - |
|  |  | [P] | -0.042 | 0.477 | - | - | 0.190 | **0.003** |
|  |  | NRE | - | - | - | - | - | - |
|  |  | PRE | - | - | - | - | - | - |
| **Increased precipitation** | Latitude | [C] | -0.478 | 21.135 | - | - | 0.672 | **0.001** |
|  |  | [N] | - | - | - | - | - | - |
|  |  | [P] | -0.090 | 3.895 | - | - | 0.270 | **0.008** |
|  |  | NRE | - | - | - | - | - | - |
|  |  | PRE | - | - | - | - | - | - |
|  | MAP | [C] | 0.002 | -0.385 | - | - | 0.672 | **0.001** |
|  |  | [N] | - | - | - | - | - | - |
|  |  | [P] | -0.090 | 3.895 | - | - | 0.270 | **0.008** |
|  |  | NRE | - | - | - | - | - | - |
|  |  | PRE | -0.001 | 0.090 | - | - | 0.480 | 0.006 |
|  | MAT | [C] | -0.044 | 0.414 | - | - | 0.672 | **0.001** |
|  |  | [N] | - | - | - | - | - | - |
|  |  | [P] | 0.023 | -0.110 | - | - | 0.183 | **0.033** |
|  |  | NRE | - | - | - | - | - | - |
|  |  | PRE | 0.919 | 0.832 | 2.546 | -0.861 | 0.542 | 0.043 |
| **N addition** | Latitude | [C] | - | - | - | - | - | - |
|  |  | [N] | - | - | - | - | - | - |
|  |  | [P] | 522.348 | 1303.451 | 59.469 | -522.155 | 0.063 | **0.012** |
|  |  | NRE | - | - | - | - | - | - |
|  |  | PRE | - | - | - | - | - | - |
|  | MAP | [C] | -0.001 | 0.402 | - | - | 0.257 | **<0.0001** |
|  |  | [N] | -9.76E-05 | 0.263 | - | - | 0.074 | **0.000** |
|  |  | [P] | -0.000 | 0.214 | - | - | 0.088 | **<0.0001** |
|  |  | NRE | - | - | - | - | - | - |
|  |  | PRE | -0.0001 | 0.001 | - | - | 0.180 | **0.010** |
|  | MAT | [C] | -0.019 | 0.163 | - | - | 0.142 | **0.001** |
|  |  | [N] | -0.008 | 0.250 | - | - | 0.095 | **<0.0001** |
|  |  | [P] | -0.008 | 0.167 | - | - | 0.060 | **0.001** |
|  |  | NRE | - | - | - | - | - | - |
|  |  | PRE | -0.010 | -0.023 | - | - | 0.176 | **0.011** |
| **Elevated [CO_2_]** | Latitude | [C] | - | - | - | - | - | - |
|  |  | [N] | 0.568 | 43.042 | 25.803 | -0.617 | 0.170 | **<0.0001** |
|  |  | [P] | 0.299 | 13.463 | 23.227 | -0.126 | 0.391 | **0.004** |
|  |  | NRE | - | - | - | - | - | - |
|  |  | PRE | - | - | - | - | - | - |
|  | MAP | [C] | - | - | - | - | - | - |
|  |  | [N] | - | - | - | - | - | - |
|  |  | [P] | - | - | - | - | - | - |
|  |  | NRE | - | - | - | - | - | - |
|  |  | PRE | - | - | - | - | - | - |
|  | MAT | [C] | - | - | - | - | - | - |
|  |  | [N] | 0.005 | -0.170 | - | - | 0.040 | **0.035** |
|  |  | [P] | - | - | - | - | - | - |
|  |  | NRE | - | - | - | - | - | - |
|  |  | PRE | - | - | - | - | - | - |

**Table S2** Functions for the correlations between multiple variables. See Figure 2 for the definitions of the abbreviations. The correlations were significant at *P*<0.05.

| Figure | Variable | Treatment | a | b | *r*^2^ | *P* |
| --- | --- | --- | --- | --- | --- | --- |
| 3a | LnRR of the C:N ratio vs lnRR [C] | Warming | - | - | - | - |
| 3b |  | Irrigation | 0.511 | 0.007 | 0.727 | **0.000** |
| 3c |  | Drought | - | - | - | - |
| 3d |  | N addition | - | - | - | - |
| 3e |  | Elevated [CO_2_] | - | - | - | - |
| 3f | LnRR of the C:N ratio vs lnRR [N] | Warming | -0.913 | -0.007 | 0.861 | **<0.0001** |
| 3g |  | Irrigation | - | - | - | - |
| 3h |  | Drought | -0.849 | 0.015 | 0.947 | **<0.0001** |
| 3i |  | N addition | -0.991 | -0.006 | 0.892 | **<0.0001** |
| 3j |  | Elevated [CO_2_] | -0.952 | 0.004 | 0.912 | **<0.0001** |
| 4a | LnRR of the N:P ratio vs lnRR [N] | Warming | 0.974 | 0.019 | 0.499 | **<0.0001** |
| 4b |  | Irrigation | 1.045 | -0.063 | 0.200 | **0.012** |
| 4c |  | Drought | 0.293 | -0.011 | 0.088 | 0.084 |
| 4d |  | N addition | 0.967 | -0.054 | 0.304 | **<0.0001** |
| 4e |  | Elevated [CO_2_] | 0.578 | -0.053 | 0.169 | **<0.0001** |
| 4f | LnRR of the N:P ratio vs lnRR [P] | Warming | -0.972 | -0.060 | 0.474 | **<0.0001** |
| 4g |  | Irrigation | -1.010 | 0.098 | 0.817 | **<0.0001** |
| 4h |  | Drought | -0.515 | 0.097 | 0.329 | **0.000** |
| 4i |  | N addition | -0.985 | 0.130 | 0.675 | **<0.0001** |
| 4j |  | Elevated [CO_2_] | -0.768 | -0.070 | 0.544 | **<0.0001** |
| 5b | LnRR of the C:N ratio vs increased precipitation | - | 0.000 | 0.077 | 0.127 | **0.002** |
| 5c | LnRR of the C:N ratio vs N-addition rate | - | 1525.194 | 510.935 | 0.448 | **<0.0001** |
| 5d | LnRR of the C:N ratio vs elevated [CO_2_] | - | 0.001 | -0.060 | 0.048 | 0.059 |
| 5f | LnRR of the N:P ratio vs increased precipitation | - | -0.000 | 0.143 | 0.124 | **0.005** |
| 5g | LnRR of the N:P ratio vs N-addition rate | - | 0.017 | -0.091 | 0.083 | **<0.0001** |

**Table S3** The model results for multiple regressions of "LnRR = Latitude + MAP + MAT + Branch length + Treatment duration + Treatment level". The model was significant when model *P* < 0.05. n represents the number of dataset used in the multiple regressions. [CO2]: carbon dioxide concentration; MAP: mean annual precipitation; MAT: mean annual temperature.

| Global change | Variable | Latitude | MAP | MAT | Branch length | Treatment duration | Treatment level | Model *r*^2^ | Model *P* | n |
| --- | --- | --- | --- | --- | --- | --- | --- | --- | --- | --- |
| Warming | foliar C:N ratio | 0.02 | 0.92 | 0.01 | 0.67 | 0.96 | 0.18 | 0.26 | 0.14 | 37 |
|  | foliar N:P ratio | 0.08 | 0.54 | 0.84 | 0.66 | 0.05 | 0.85 | 0.41 | 0.18 | 22 |
| Increased precipitation | foliar C:N ratio | 0.05 | NA | NA | 0.84 | NA | NA | 0.34 | 0.12 | 13 |
|  | foliar N:P ratio | 0.63 | NA | NA | 0.27 | NA | NA | 0.13 | 0.51 | 13 |
| N addition | foliar C:N ratio | **<0.0001** | **0.02** | **0.0009** | 0.24 | 0.22 | **0.0009** | 0.43 | **<0.0001** | 78 |
|  | foliar N:P ratio | **<0.0001** | 0.97 | 0.09 | 0.43 | **<0.0001** | **0.03** | 0.35 | **<0.0001** | 155 |
| Elevated [CO_2_] | foliar C:N ratio | **0.0006** | **0.05** | **0.01** | **0.01** | **0.003** | 0.42 | 0.87 | **<0.0001** | 23 |
|  | foliar N:P ratio | 0.86 | 0.9 | 0.76 | 0.79 | NA | NA | 0.04 | 0.97 | 17 |

**Figure S1** Distribution of experimental sites included in this meta-analysis, showing different global change treatments, where CO_2_ is carbon dioxide and N is nitrogen. This map was generated in R version 3.4.2 (R Core Team, 2017, [https://www.R-project.org/](https://www.r-project.org/)) using ggplot2 and the R packages ggsn and legendMap.


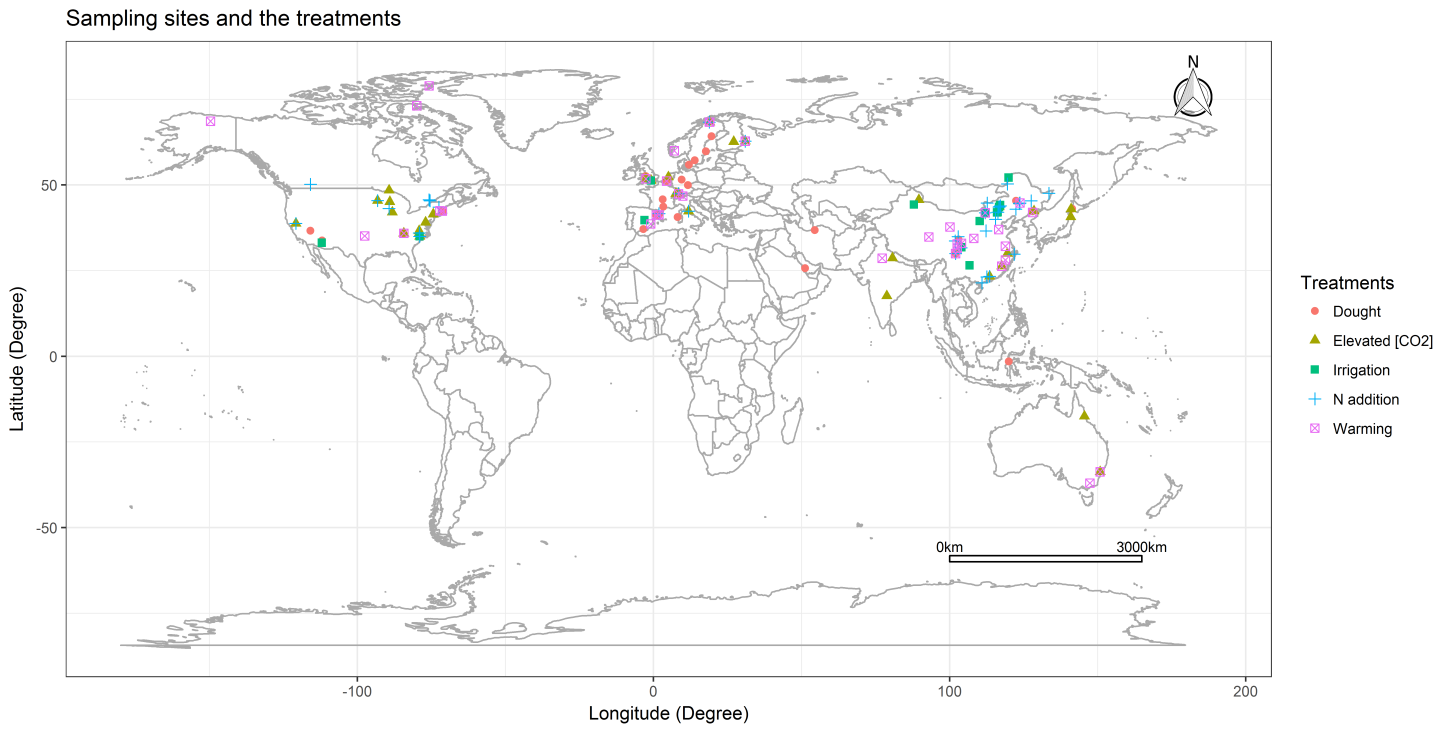


**Figure S2** Normal distribution of LnRR of foliar [C], foliar [N], foliar [P], foliar C:N, foliar N:P, foliar NRE, foliar PRE under warming. LnRR: natural log of response ratio; foliar [C]: foliar carbon concentration; foliar [N]: foliar nitrogen concentration; foliar [P]: foliar phosphorus concentration; foliar C:N: foliar carbon to nitrogen ratio; foliar N:P: foliar nitrogen to phosphorus ratio; NRE: nitrogen resportion efficiency; PRE: phosphorus resportion efficiency.

**
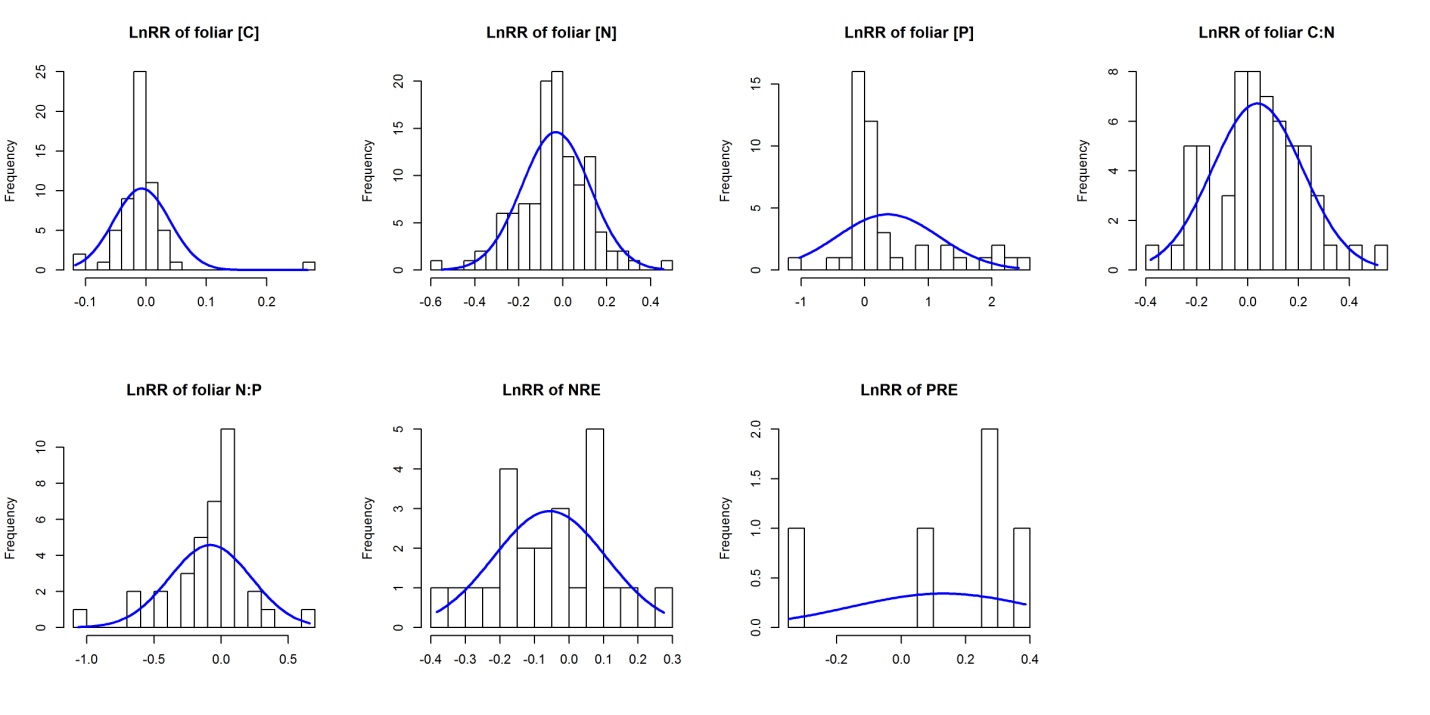
**

**Figure S3** Normal distribution of LnRR of foliar [C], foliar [N], foliar [P], foliar C:N, foliar N:P, foliar NRE, PRE under increased precipitation. The abbreviations follow the legend of figure S2.

**
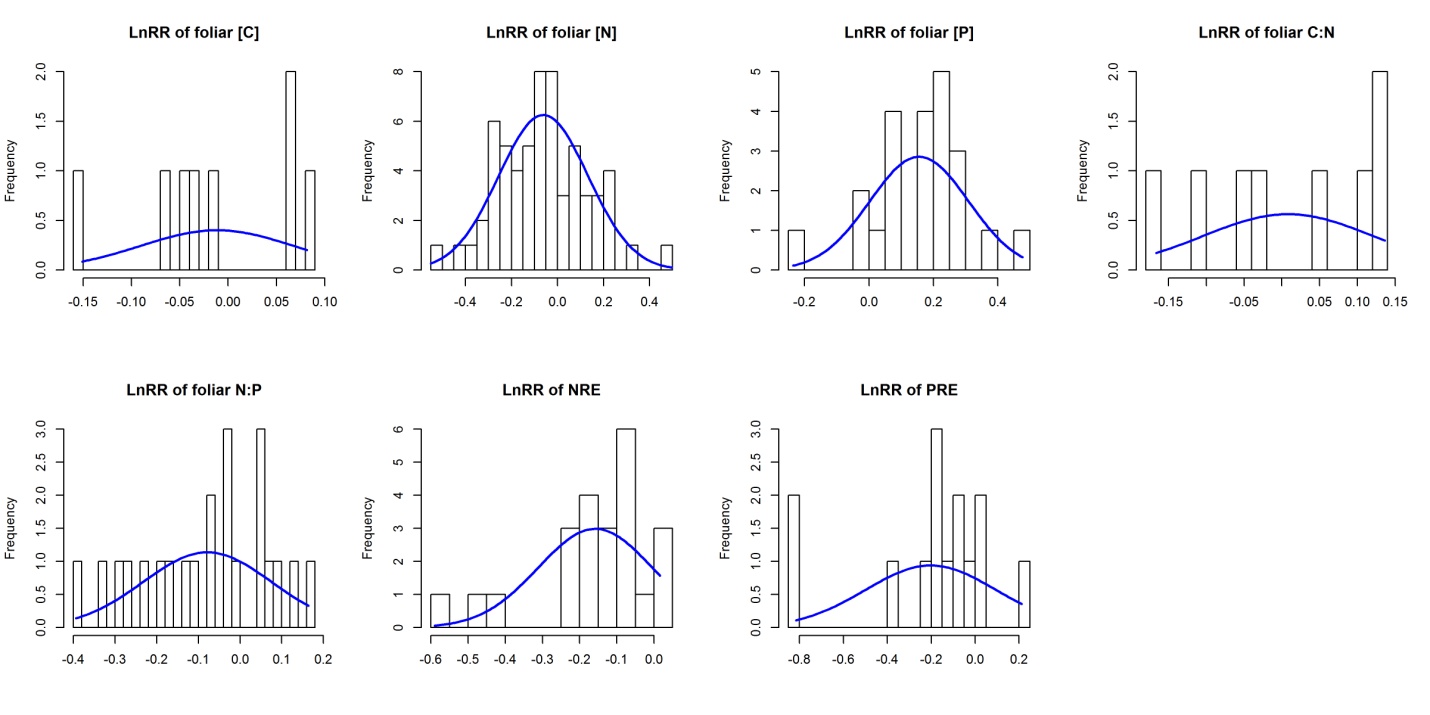
**

**Figure S4** Normal distribution of LnRR of foliar [C], foliar [N], foliar [P], foliar C:N, foliar N:P, foliar NRE, PRE under drought. The abbreviations follow the legend of figure S2.

**
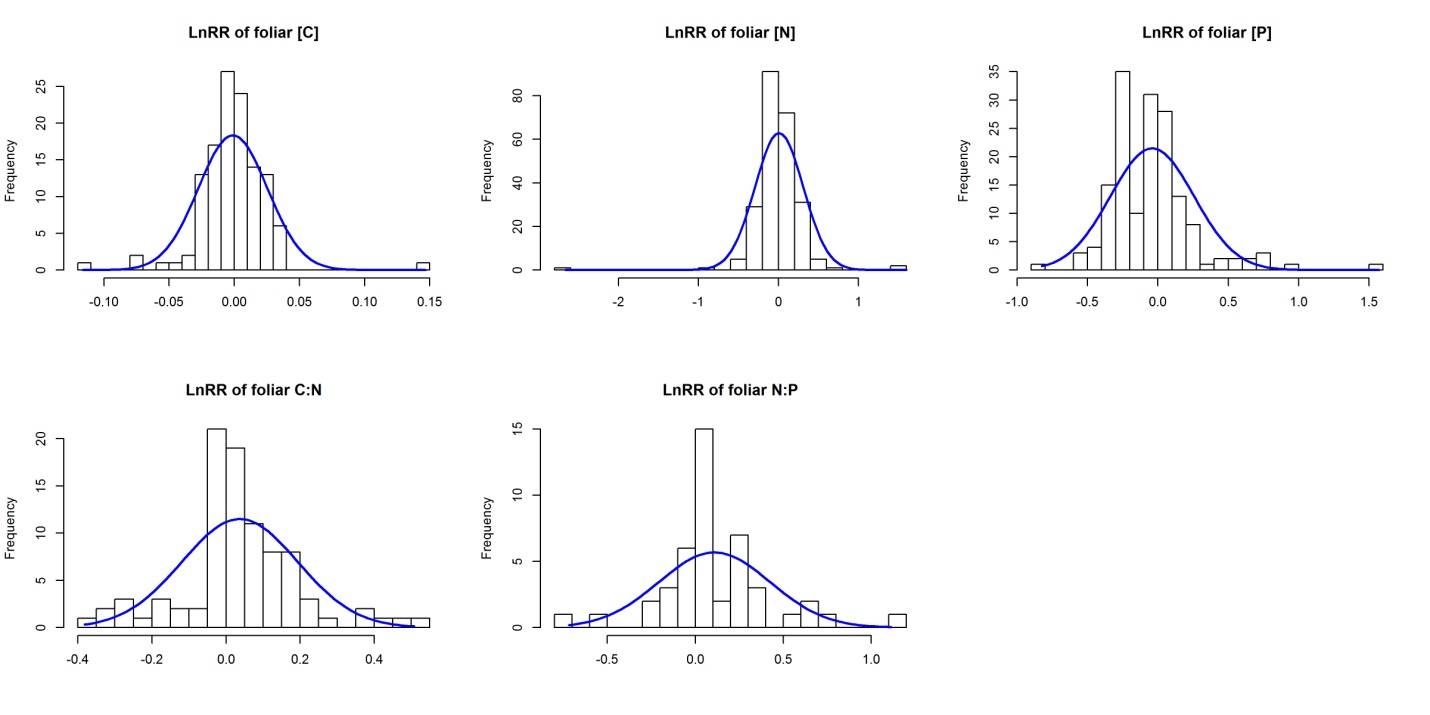
**

**Figure S5** Normal distribution of LnRR of foliar [C], foliar [N], foliar [P], foliar C:N, foliar N:P, foliar NRE, PRE under N addition. The abbreviations follow the legend of figure S2.

**
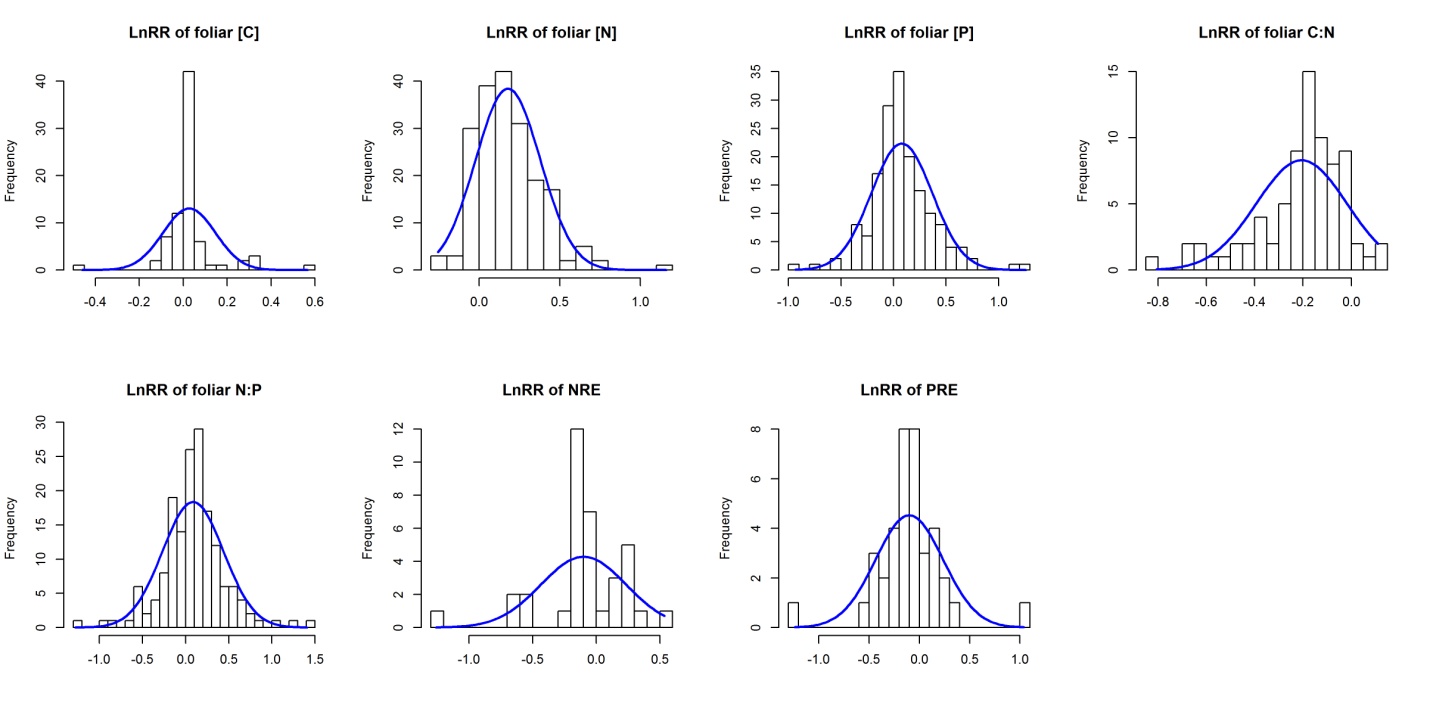
**

**Figure S6** Normal distribution of LnRR of foliar [C], foliar [N], foliar [P], foliar C:N, foliar N:P, foliar NRE, PRE under elevated CO_2_. The abbreviations follow the legend of figure S2.

**
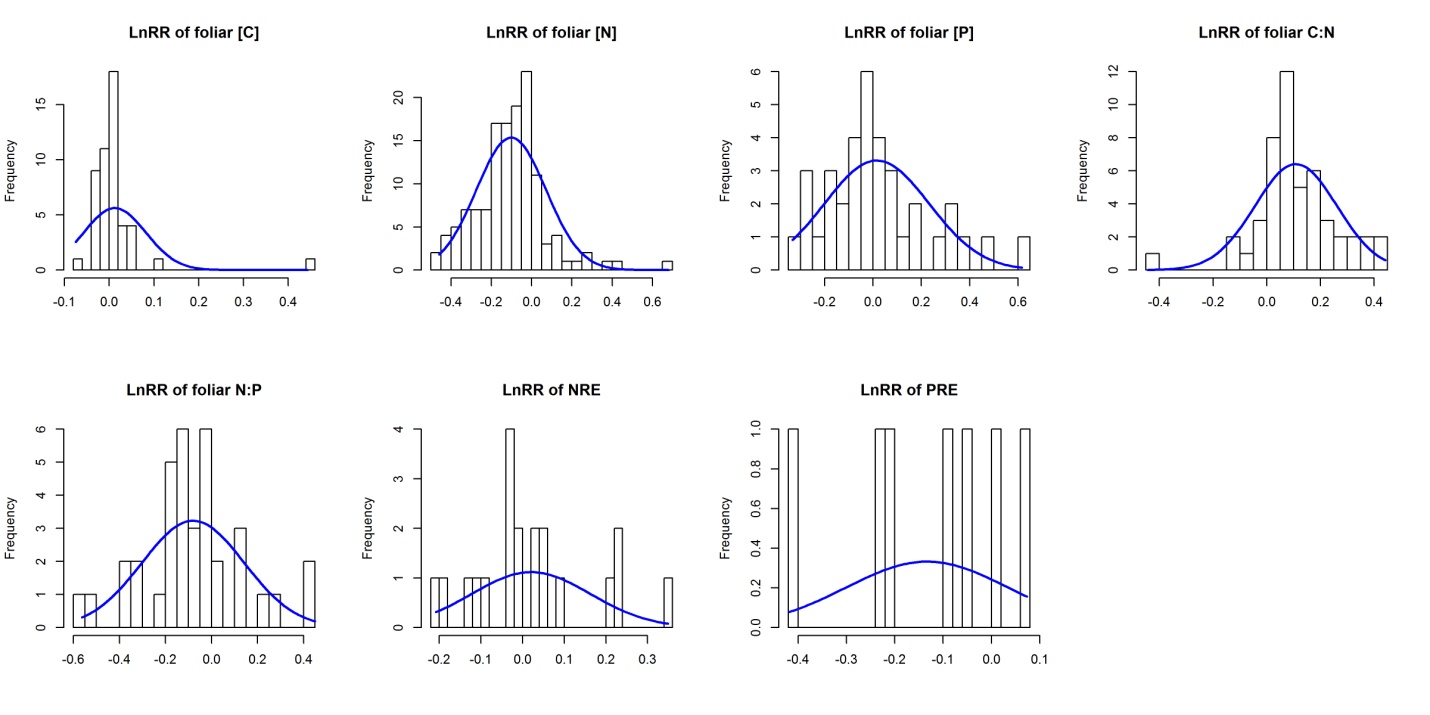
**

**Figure S7** Phylogeny for 218 plant species used in this study, obtained from the R package "V.PhyloMaker" in the R version 3.3.3 (R Core Team, 2017, [https://www.R-project.org/](https://www.r-project.org/)).


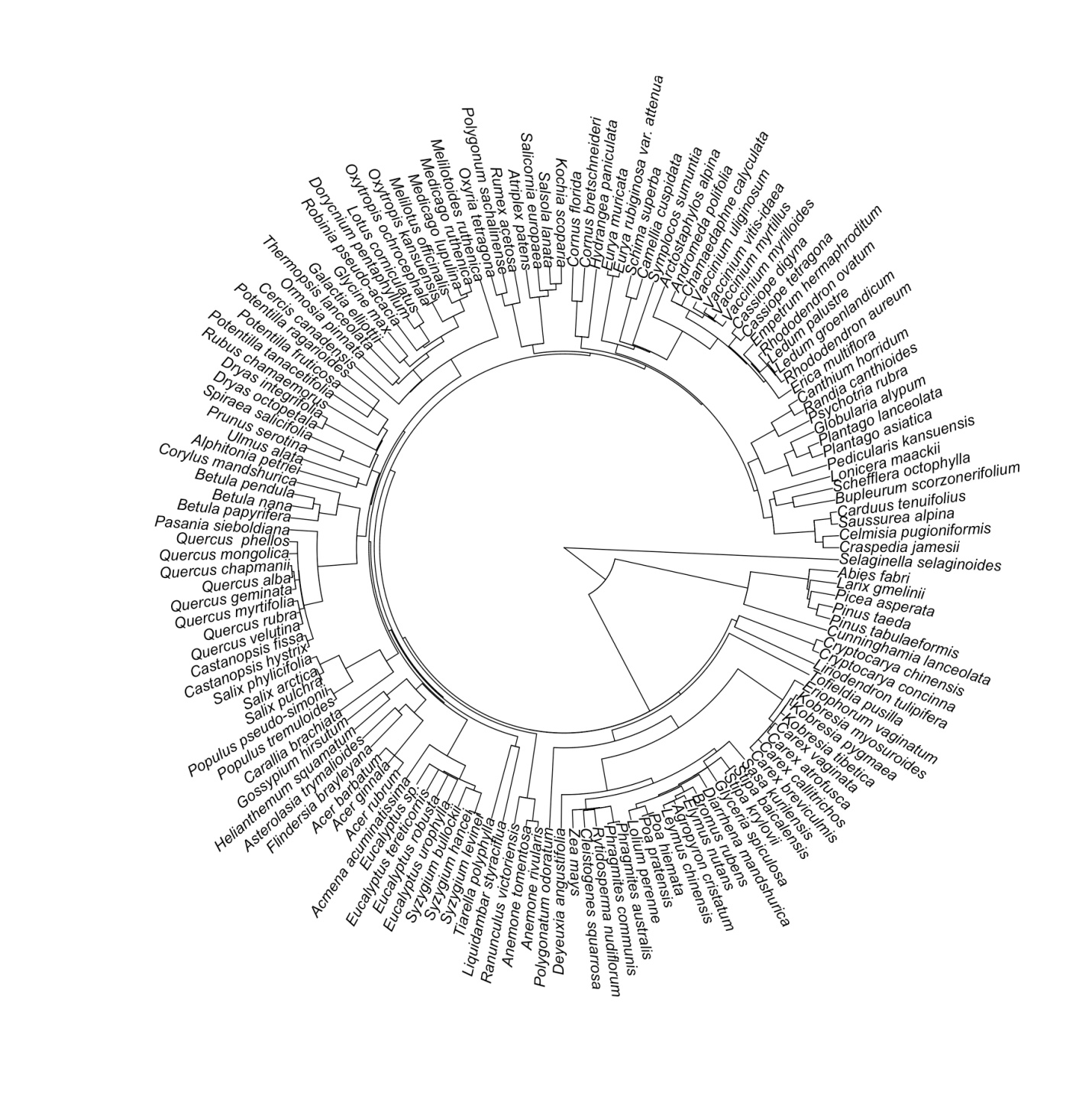


**Figure S8** Correlations between the response ratios of foliar C:N ratio, foliar N:P ratio and the branch length of each species. N, nitrogen; C:N ratio: carbon to nitrogen ratio; N:P ratio: nitrogen to phosphorus ratio; [CO_2_], carbon dioxide concentration; LnRR, natural log of the response ratio. The correlation was significant when *P*<0.05. The figure was performed using Sigmaplot version 11.0 (Systat Software, Inc.).

**
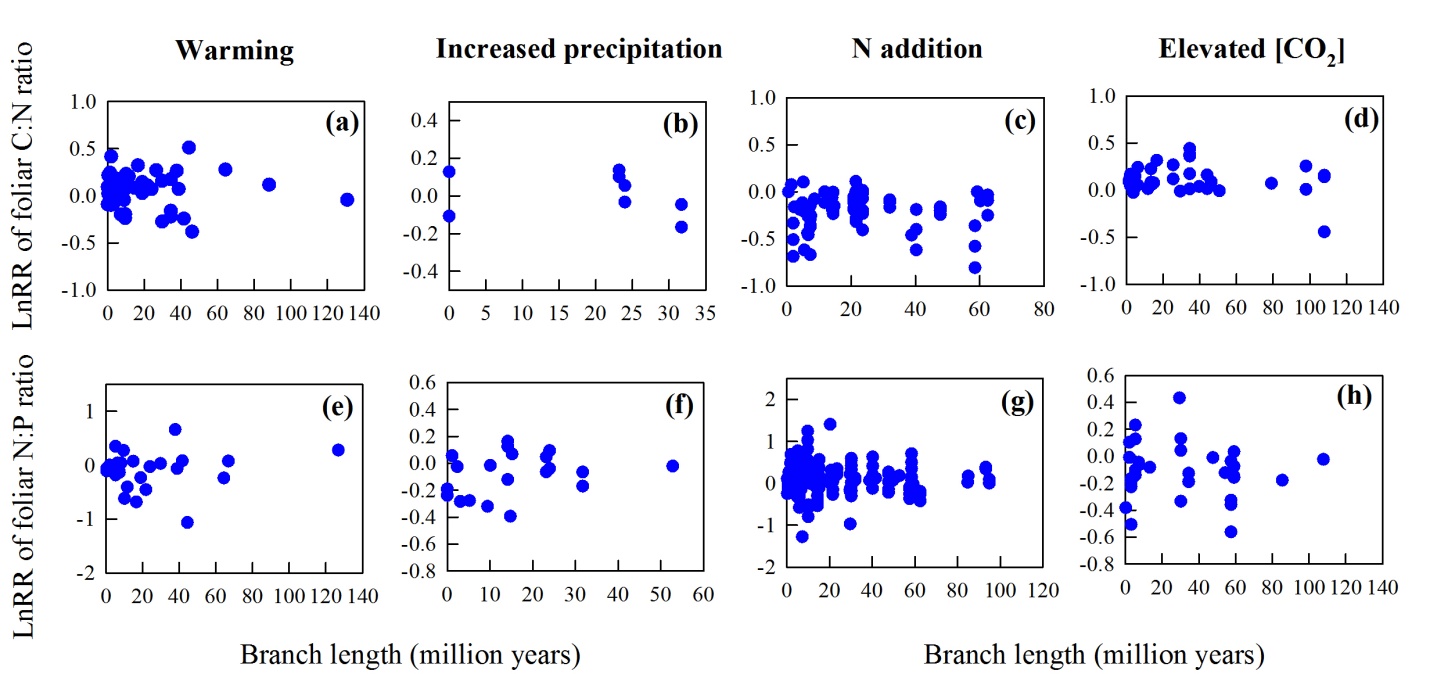
**

**Figure S9** Correlations between the response ratios of foliar [N] and N-resorption efficiency under warming, irrigation, N addition and elevated [CO_2_]. N, nitrogen; [N], N concentration; [CO_2_], carbon dioxide concentration; LnRR, natural log of the response ratio. The figure was performed using Sigmaplot version 11.0 (Systat Software, Inc.).

**
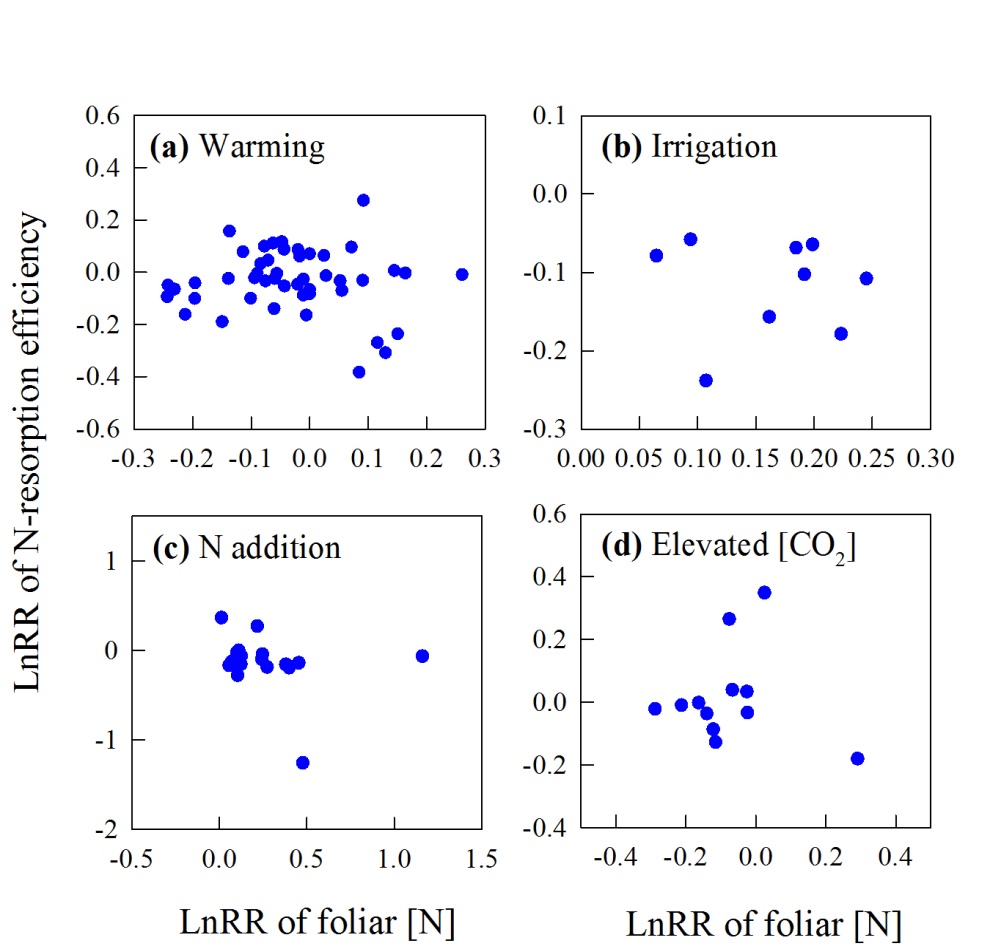
**

**Figure S10** Correlations between the response ratios of foliar [P] and P-resorption efficiency under warming, irrigation, N addition and elevated [CO_2_]. N, nitrogen; P, phosphorus; [P], P concentration; [CO_2_], carbon dioxide concentration; LnRR, natural log of the response ratio. The figure was performed using Sigmaplot version 11.0 (Systat Software, Inc.).


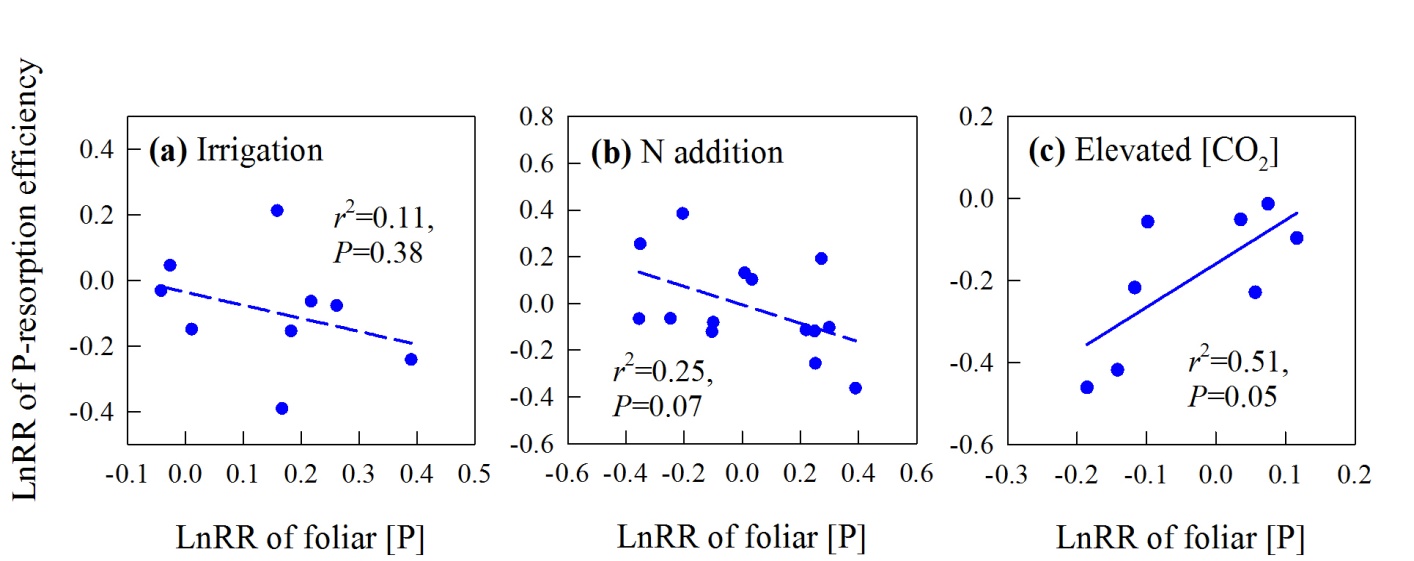


**Figure S11** Correlations between the response ratios of foliar [C], [N], [P] and the branch length of each species. N, nitrogen; [CO_2_], carbon dioxide concentration; LnRR, natural log of the response ratio; C], carbon concentration; [N], N concentration; [P], P concentration. The correlation was significant when *P*<0.05. The figure was performed using Sigmaplot version 11.0 (Systat Software, Inc.).

**
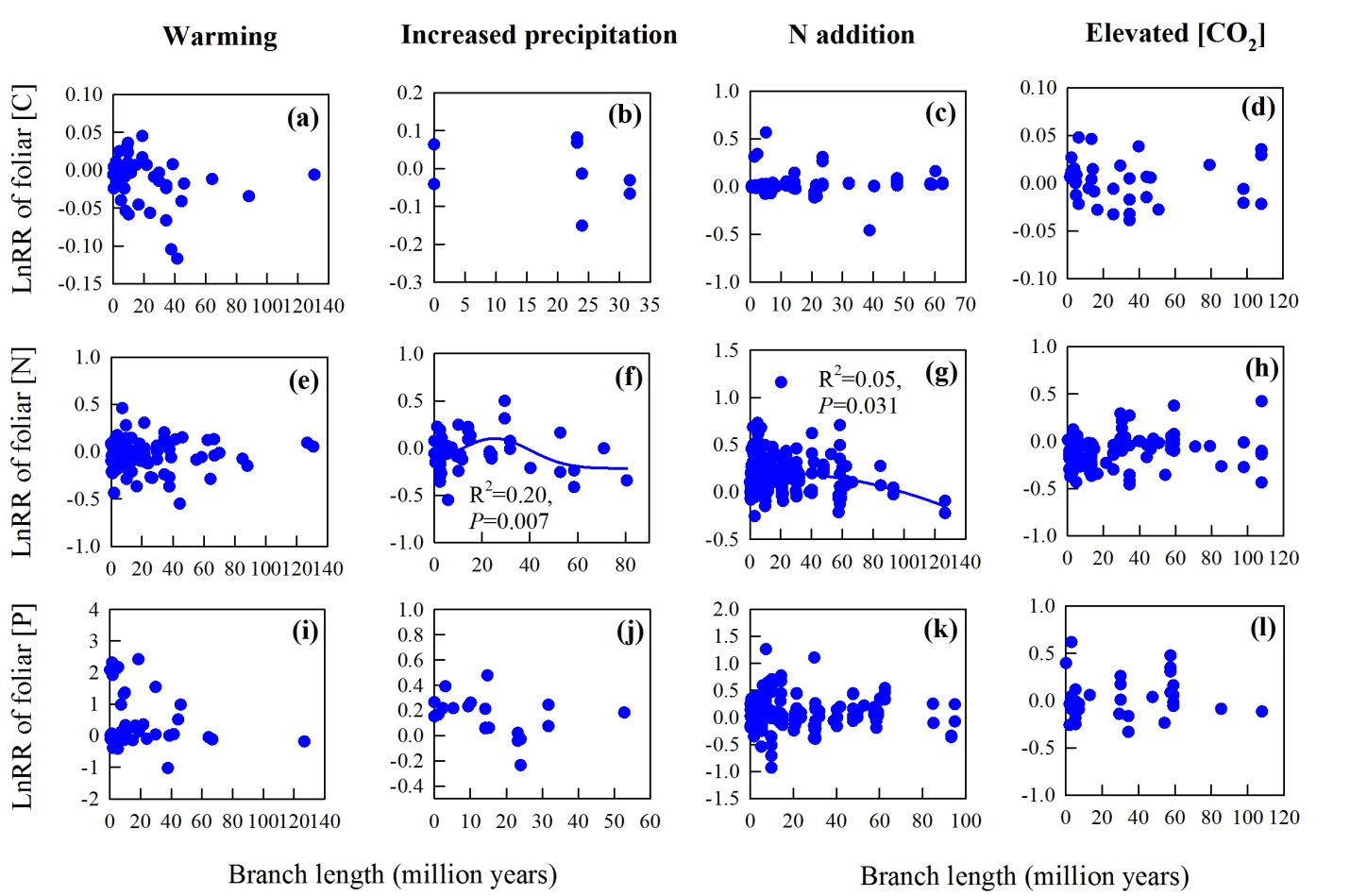
**

**Figure S12** Correlations between the response ratios of foliar [C], [N] and [P] and the treatment levels of warming, changed precipitation, N addition and elevated [CO_2_]. N, nitrogen; [N], N concentration; P, phosphorus; [P], P concentration; [C], carbon concentration; [CO_2_], carbon dioxide concentration; LnRR, natural log of the response ratio. The correlation was significant when *P*<0.05. The figure was performed using Sigmaplot version 11.0 (Systat Software, Inc.).

**
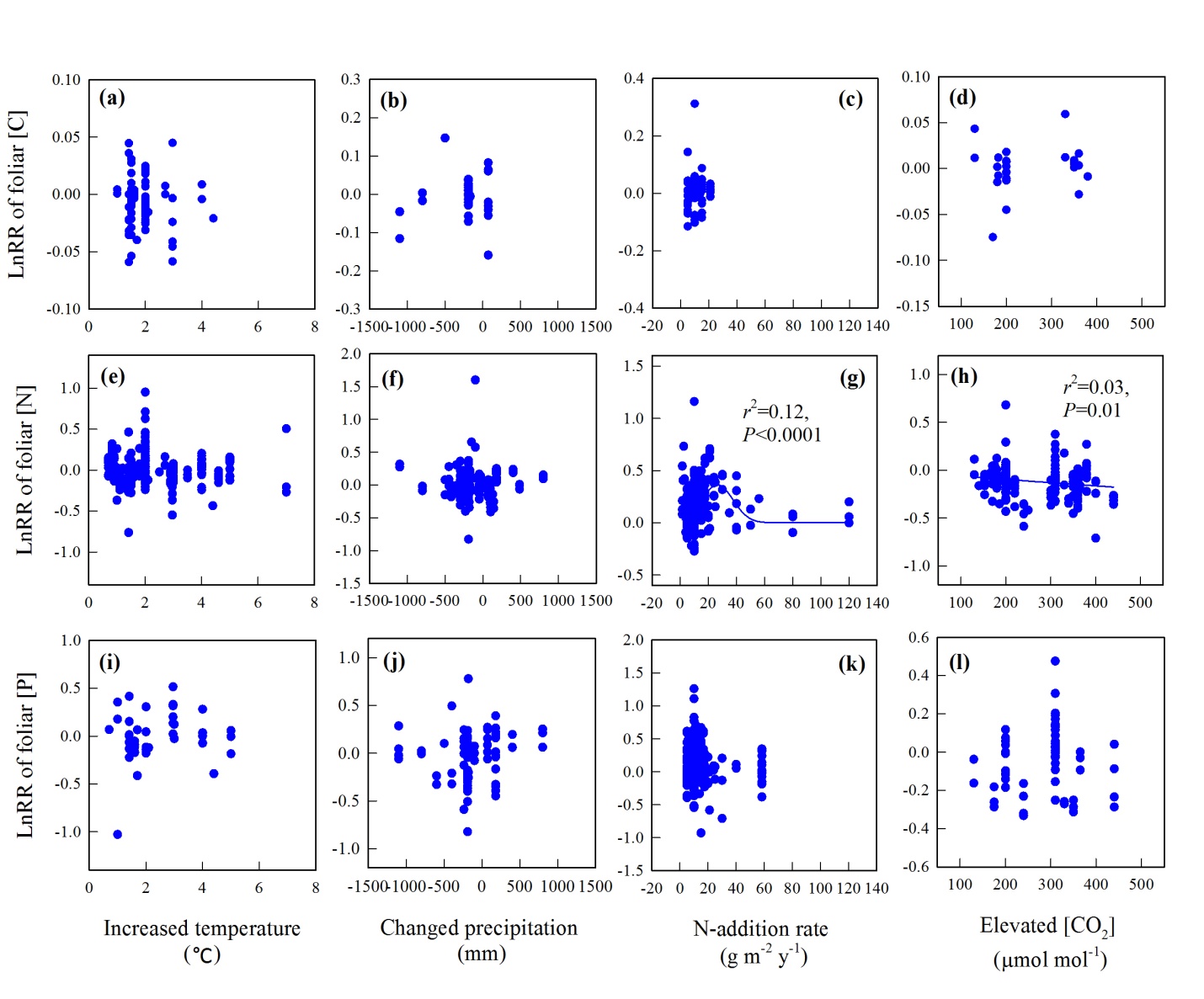
**

**Figure S13** Correlations between the response ratios of foliar [C], [N] and [P] and the treatment duration of warming, changed precipitation, N addition and elevated [CO_2_]. N, nitrogen; [N], N concentration; P, phosphorus; [P], P concentration; [C], carbon concentration; [CO_2_], carbon dioxide concentration; LnRR, natural log of the response ratio. The correlation was significant when *P*<0.05. The figure was performed using Sigmaplot version 11.0 (Systat Software, Inc.).

**
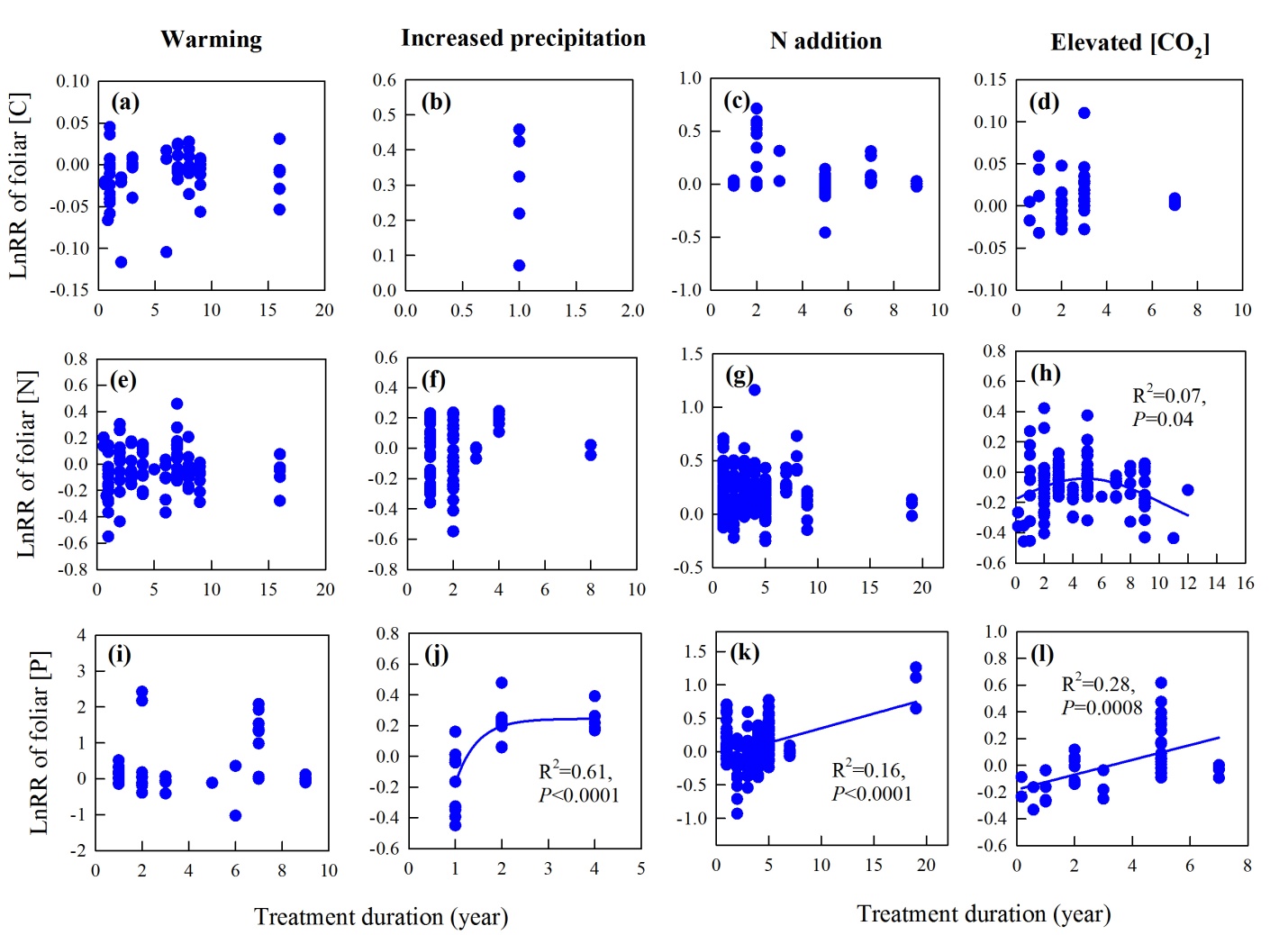
**

**Papers included in this dataset**

1. Aerts, R., Callaghan, T.V., Dorrepaal, E., van Logtestijn, R.S.P. & Cornelissen, J.H.C. Seasonal climate manipulations result in species-specific changes in leaf nutrient levels and isotopic composition in a sub-arctic bog. *Funct. Ecol.* **23,** 680-688 (2009).

2. Aerts, R., Cornelissen, J.H., van Logtestijn, R.S. & Callaghan, T.V. Climate change has only a minor impact on nutrient resorption parameters in a high-latitude peatland. *Oecologia* **151,** 132-139 (2007).

3. An, Y. *et al*. Plant nitrogen concentration, use efficiency, and contents in a tallgrass prairie ecosystem under experimental warming. *Glob. Change Biol.* **11,** 1733-1744 (2005).

4. Bader, M.K., Siegwolf, R. & Korner, C. Sustained enhancement of photosynthesis in mature deciduous forest trees after 8 years of free air CO(_2_) enrichment. *Planta* **232,** 1115-1125 (2010).

5. Billings, [S.A](https://onlinelibrary.wiley.xilesou.top/action/doSearch?ContribAuthorStored=BILLINGS%2C+SHARON+A). *et al*. Effects of elevated carbon dioxide on green leaf tissue and leaf litter quality in an intact Mojave Desert ecosystem. *Glob. Change Biol.* **9,** 729-735 (2003a).

6. Bin, Z.J. *et al*. Effects of N addition on ecological stoichiometric characteristics in six dominant plant species of alpine meadow on the Qinghai-Xizang Plateau, China. *Chin. J. Plant Ecol.* **38,** 231-237 (2014).

7. Booker, F.L., Shafer, S.R., Wei, C.M. & Horton, S.J. Carbon dioxide enrichment and nitrogen fertilization effects on cotton (*Gossypium hirsutum* L.) plant residue chemistry and decomposition. *Plant Soil* **220,** 89–98 (2000).

8. Booker, F.L. & Maier, [C.A.](https://a.glgoo.top/citations?user=0-Gn_TgAAAAJ&hl=zh-CN&oi=sra) Atmospheric carbon dioxide, irrigation, and fertilization effects on phenolic and nitrogen concentrations in loblolly pine (Pinus taeda) needles. *Tree Physiol.* **21,** 609–616 (2001a).

9. Broadmeadow, M.S.J. & Jackson, S.B. Growth responses of *Quercus petraea*, *Fraxinus excelsior* and *Pinus sylvestris* to elevated carbon dioxide, ozone and water supply. *New Phytol.* **146,** 437–451 (2000).

10. Bubier, J.L. *et al*. Effects of nutrient addition on leaf chemistry, morphology, and photosynthetic capacity of three bog shrubs. *Oecologia* **167,** 355-368 (2011).

11. Butler, S.M. *et al*. Soil warming alters nitrogen cycling in a New England forest: implications for ecosystem function and structure. *Oecologia* **168,** 819-828 (2012).

12. Calfapietra, [C](javascript:;). *et al*. Increased nitrogen-use efficiency of a short-rotation poplar plantation in elevated CO_2_ concentration. *Tree Physiol.* **27,** 1153–1163 (2007a).

13. Cao, B., Dang, Q.L. & Zhang, S. Relationship between photosynthesis and leaf nitrogen concentration in ambient and elevated [CO_2_] in white birch seedlings. *Tree Physiol.* **27,** 891–899 (2007b).

14. Chen, L., Zhao, J., Zhang, R., Wang, S. & Wang, G. Effects of nitrogen and phosphorus fertilization on legumes in *Potentilla fruticosa* shrub in alpine meadow. *Ecol. Sci.* **29,** 512-517 (2010).

15. Chen, F.S., *et al*. Nitrogen and phosphorus additions alter nutrient dynamics but not resorption efficiencies of Chinese fir leaves and twigs differing in age. *Tree Physiol.* **35,** 1106-1117 (2015).

16. Choi, W.J., Chang, S.X., Allen, H.L., Kelting, D.L. & Ro, H.M. Irrigation and fertilization effects on foliar and soil carbon and nitrogen isotope ratios in a loblolly pine stand. *For. Ecol. Manage.* **213,** 90-101 (2005).

17. Couture, J.J., Meehan, T.D. & Lindroth, R.L. Atmospheric change alters foliar quality of host trees and performance of two outbreak insect species. *Oecologia* **168,** 863-876 (2011).

18. Crous, K.Y., Reich, P.B., Hunter, M.D. & Ellsworth, D.S. Maintenance of leaf N controls the photosynthetic CO_2_ response of grassland species exposed to 9 years of free-air CO_2_ enrichment. *Glob. Change Biol.* **16,** 2076-2088 (2010).

19. Dermody, O., O’Neill, B.F., Zangerl, A.R., Berenbaum, M.R. & DeLucia, E.H. Effects of elevated CO_2_ and O_3_ on leaf damage and insect abundance in a soybean agroecosystem. *Arthropod-Plant Interact.* **2,** 125-135 (2008).

20. Doiron, M., Gauthier, G., Lévesque, E. & Newman, J. Effects of experimental warming on nitrogen concentration and biomass of forage plants for an arctic herbivore. *J. Ecol.* **102,** 508-517 (2014).

21. Duan, H. Changes in leaf nutrient traits and photosynthesis of four tree species: effects of elevated [CO_2_], N fertilization and canopy positions. *J. Plant Ecol.* **5,** 376-390 (2012).

22. Dury, S.J., Good, J.E.G., Perrins, C.M., Buse, A. & Kaye, T. The effects of increasing CO_2_ and temperature on oak leaf palatability and the implications for herbivorous insects. *Glob. Change Biol.* **4,** 55–61 (1998).

23. Esmeijer-Liu, A.J. *et al*. Nitrogen enrichment lowers *Betula pendula* green and yellow leaf stoichiometry irrespective of effects of elevated carbon dioxide. *Plant Soil* **316,** 311-322. (2008).

24. Finzi, [A.C.](https://esajournals.onlinelibrary.wiley.com/action/doSearch?ContribAuthorStored=Finzi%2C+Adrien+C), Allen, [A.S.](https://esajournals.onlinelibrary.wiley.com/action/doSearch?ContribAuthorStored=Allen%2C+Andrew+S), DeLucia, [E.H.,](https://esajournals.onlinelibrary.wiley.com/action/doSearch?ContribAuthorStored=DeLucia%2C+Evan+H) Ellsworth, [D.S. &](https://esajournals.onlinelibrary.wiley.com/action/doSearch?ContribAuthorStored=Ellsworth%2C+David+S) Schlesinger, [W.H.](https://esajournals.onlinelibrary.wiley.com/action/doSearch?ContribAuthorStored=Schlesinger%2C+William+H) Forest litter production, chemistry, and decomposition following two years of free‐air CO_2_ enrichment. *Ecology* **82,** 470–484 (2001b).

25. García-Palacios, P., Querejeta, J.I., Maestre, F.T., Escudero, A. & Valladares, F. Impact of simulated changes in rainfall regime and nutrient deposition on the relative dominance and isotopic composition of ruderal plants in anthropogenic grasslands. *Plant Soil* **352,** 303-319 (2011).

26. Gao, Z., Wang, H., Lv, X. & Wang, Z. Effects of nitrogen and phosphorus addition on C:N:P stoichiometry in roots and leaves of four dominant plant species in a meadow steppe of Hulunbuir. *Chin. J. Ecol.* **36,** 80-88 (2017).

27. Gherlenda, A.N., Haigh, A.M., Moore, B.D., Johnson, S.N. & Riegler, M. Responses of leaf beetle larvae to elevated [CO(_2_)] and temperature depend on *Eucalyptus* species. *Oecologia* **177,** 607-617 (2015).

28. Guo, S.Q., Li, W.J., Zhang, R.Y. & Wang, G. Effects of N and P additions on foliar stoichiometry and photosynthetic characteristics of *Potentilla fruiticosa*. *Guihaia* **34,** 629-634 (2014).

29. Gunthardt-Goerg, M.S. & Vollenweider, P. Responses of beech and spruce foliage to elevated carbon dioxide, increased nitrogen deposition and soil type. *AoB Plants* **7** (2015).

30. Hall, M.C., Stiling, P., Moon, D.C., Drake, B.G. & Hunter, M.D. Effects of elevated CO_2_ on foliar quality and herbivore damage in a scrub oak ecosystem. *J. Chem. Ecol.* **31,** 267-286 (2005).

31. Housman, D.C., Killingbeck, K.T., Dave, E.R., Charlet, T.N. & Smith, S.D. Foliar nutrient resorption in two Mojave Desert shrubs exposed to Free-Air CO_2_ Enrichment (FACE). *J. Arid. Environ.* **78,** 26-32 (2012).

32. Huang, W. *et al*. Effects of elevated carbon dioxide and nitrogen addition on foliar stoichiometry of nitrogen and phosphorus of five tree species in subtropical model forest ecosystems. *Environ. Pollut.* **168,** 113-120 (2012).

33. Hudson, J.M.G., Henry, G.H.R. & Cornwell, W.K. Taller and larger: shifts in Arctic tundra leaf traits after 16 years of experimental warming. *Glob. Change Biol.* **17,** 1013-1021 (2011).

34. Huttunen L. *et al*. Effects of elevated temperature, elevated CO_2_ and fertilization on quality and subsequent decomposition of silver birch leaf litter. *Soil Biol. Biochem.* **41,** 2414–2421 (2009).

35. Jach-Smith, L.C. & Jackson, R.D. Nitrogen conservation decreases with fertilizer addition in two perennial grass cropping systems for bioenergy. *Agric. Ecosyst. Environ.* **204,** 62-71 (2015).

36. Jiang, X.J., Hu, Y.L., Han, J.Q. & Zhou, Y.M. Effects of warming on carbon, nitrogen and phosphorus stoichiometry in tundra soil and leaves of typical plants. *Chin. J. Plant Ecol.* **38,** 941-948 (2014).

37. Johns, [C.V](https://a.glgoo.top/citations?user=Ih_wK2kAAAAJ&hl=zh-CN&oi=sra). & Hughes, [L.](https://a.glgoo.top/citations?user=LGc712IAAAAJ&hl=zh-CN&oi=sra) Interactive effects of elevated CO_2_ and temperature on the leaf-miner Dialectica scalariella Zeller (Lepidoptera: Gracillariidae) in Paterson's Curse, Echium plantagineum (Boraginaceae). *Glob. Change Biol.* **8,** 142-152 (2002).

38. Johnson, D.W., Ball, J.T. & Walker, R.F. Effects of CO_2_ and nitrogen fertilization on vegetation and soil nutrient content in juvenile ponderosa pine. *Plant Soil* **190,** 29–40 (1997).

39. Kanowski, J. Effects of elevated CO_2_ on the foliar chemistry of seedlings of two rainforest trees from north-east Australia: Implications for folivorous marsupials. *Austral Ecol.* **26,** 165–172 (2001).

40. Kim, S.H. *et al*. Canopy photosynthesis, evapotranspiration, leaf nitrogen, and transcription profiles of maize in response to CO_2_ enrichment. *Glob. Change Biol.* **12,** 588-600 (2006).

41. Knepp, R.G. *et al*. Elevated CO_2_ reduces leaf damage by insect herbivores in a forest community. *New Phytol.* **167,** 207-218 (2005).

42. Kuokkanen, K., Julkunen-Tiitto, R., Keinänen, M., Niemelä, P. & Tahvanainen, J. The effect of elevated CO_2_ and temperature on the secondary chemistry of Betula pendula seedlings. *Trees* **15,** 378-384 (2001).

43. Kuokkanen, K., Yan, S. & Niemela¨, P. Effects of elevated CO_2_ and temperature on the leaf chemistry of birch *Betula pendula* (Roth) and the feeding behaviour of the weevil Phyllobius maculicornis. *Agric. For. Entomol.* **5,** 209–217 (2003).

44. Lavola, A. *et al*. Combination treatment of elevated UVB radiation, CO_2_ and temperature has little effect on silver birch (*Betula pendula*) growth and phytochemistry. *Physiol. Plant* **149,** 499-514 (2013).

45. León-Sánchez, L., Nicolás, E., Nortes, P.A., Maestre, F.T. & Querejeta, J.I. Photosynthesis and growth reduction with warming are driven by nonstomatal limitations in a Mediterranean semi-arid shrub. *Ecol. Evol.* **6,** 2725-2738 (2016).

46. Li, D.J., Mo, J.M., Peng, S.L. & Fang, Y.T. Effects of simulated nitrogen deposition on elemental concentrations of *Schima superba* and *Cryptocarya concinna* seedlings in subtropical China. *ACTA Ecol. Sinica* **25,** 2165-2172 (2005).

47. Li, [F](https://esajournals.onlinelibrary.wiley.com/action/doSearch?ContribAuthorStored=Li%2C+Fei). *et al*. Warming effects on permafrost ecosystem carbon ﬂuxes associated with plant nutrients. *Ecology* **0,** 1-9 (2017).

48. Li, L.J., Zeng, D.H., Mao, R. & Yu, Z.Y. Nitrogen and phosphorus resorption of *Artemisia scoparia*, *Chenopodium acuminatum*, *Cannabis sativa*, and *Phragmites* communis under nitrogen and phosphorus additions in a semiarid grassland, China. *Plant Soil Environ.* **58,** 446–451 (2012).

49. Li, M.H. *et al*. Responses of leaf nitrogen and mobile carbohydrates in different Quercus species/provenances to moderate climate changes. *Plant Biol.* **15,** 177-184 (2013).

50. Li, X. *et al*. Combined effects of nitrogen addition and litter manipulation on nutrient resorption of Leymus chinensis in a semi-arid grassland of northern China. *Plant Biol.* **17,** 9-15 (2015).

51. Li, Y. *et al*. Nitrogen resorption efficiency of perennial plant decreases with warming and nitrogen addition in desert steppe. *ACTA Ecol. Sinica* **35,** 5948-5956 (2015).

52. Li, Y. *et al*. Effects of warming on ectomycorrhizal colonization and nitrogen nutrition of *Picea asperata* seedlings grown in two contrasting forest ecosystems. *Sci. Rep.* **5,** 17546-17555 (2015).

53. Lindroth, R.L. *et al*. Consequences of elevated carbon dioxide and ozone for foliar chemical composition and dynamics in trembling aspen (*Populus tremuloides*) and paper birch (*Betula papyrifera*). *Environ. Pollut.* **115,** 395-404 (2001).

54. Liu, J. *et al*. Effects of Nitrogen Addition on Nitrogen Resorption in Temperate Shrublands in Northern China. *PloS one* **10**, e0130434 (2015).

55. Liu, J. *et al*. Photosynthesis acclimation, leaf nitrogen concentration, and growth of four tree species over 3 years in response to elevated carbon dioxide and nitrogen treatment in subtropical China. *J. Soils Sediments* **11,** 1155-1164 (2011).

56. Llorens, L., Pen˜uelas, J. & Estiarte, M. Ecophysiological responses of two Mediterranean shrubs, *Erica multiflora* and *Globularia alypum*, to experimentally drier and warmer conditions. *Physiol. Plant.* **119,** 231–243 (2003).

57. Lü, X.T. & Han, X.G. Nutrient resorption responses to water and nitrogen amendment in semi-arid grassland of Inner Mongolia, China. *Plant Soil* **327,** 481-491 (2009).

58. Luo, Z.B., Calfapietra, C., Scarascia-Mugnozza, G., Liberloo, M. & Polle, A. Carbon-based secondary metabolites and internal nitrogen pools in Populus nigra under Free Air CO_2_ Enrichment (FACE) and nitrogen fertilisation. *Plant Soil* **304,** 45-57 (2008).

59. Lu, J. The effects of nitrogen addition on leaf nutrient characteristics of dominant species in evergreen broadleaved forest in Tiantong Mountain. *Master's thesis* (2014).

60. Lu, X., Mo, J., Li, D., Zhang, W. & Fang, Y. Effects of simulated N deposition on the photosynthetic and physiologic characteristics of dominant understorey plants in Dinghushan Mountain of subtropical China. *J. Beijing For. University* **29,** 1-9 (2007).

61. Maier, [C.A](javascript:;)., Palmroth, [S](javascript:;). & Ward, [E](javascript:;). Short-term effects of fertilization on photosynthesis and leaf morphology of field-grown loblolly pine following long-term exposure to elevated CO_2_ concentration. *Tree Physiol.* **28,** 597–606 (2008).

62. Manimanjari, D., *et al*. Temperature- and CO_2_-dependent life table parameters of *Spodoptera litura* (Noctuidae: Lepidoptera) on sunflower and prediction of pest scenarios. *J. insect sci.* **14** (2014).

63. Mao, R., Song, C.C., Zhang, X.H., Wang, X.W. & Zhang, Z.H. Response of leaf, sheath and stem nutrient resorption to 7 years of N addition in freshwater wetland of Northeast China. *Plant Soil* **364,** 385-394 (2012).

64. Mao, R., Zhang, X. & Song, C. Effects of nitrogen addition on plant functional traits in freshwater wetland of Sanjiang Plain, Northeast China. *Chin. Geogr. Sci.* **24,** 674-681 (2014).

65. Matsushima, M. & Chang, S.X. Vector analysis of understory competition, N fertilization, and litter layer removal effects on white spruce growth and nutrition in a 13-year-old plantation. *For. Ecol. Manag.* **236,** 332-341 (2006).

66. Mattson, W.J., Julkunen-Tiitto, R. & Herms, D.A. CO_2_ enrichment and carbon partitioning to phenolics: do plant responses accord better with the protein competition or the growth-differentiation balance models? *Oikos* **111,** 337-347 (2005).

67. Ma, W. & Fan, W. Effect of drought stress on mineral nutrient contents in leaves of sdeedling plant of *C. tangerina* Hort., *C. sinensis* Osbeck and *C. grandis* Osbeck. *Southwest China J. Agricul. Sci.* **20,** 630-633 (2007).

68. Mayor, J.R., Wright, S.J., Turner, B.L. & Austin, A. Species-specific responses of foliar nutrients to long-term nitrogen and phosphorus additions in a lowland tropical forest. *J. Ecol.* **102,** 36-44 (2014).

69. Mcelrone, [A.J.](https://onlinelibrary.wiley.xilesou.top/action/doSearch?ContribAuthorStored=Mcelrone%2C+Andrew+J), Reid, [C.D.](https://onlinelibrary.wiley.xilesou.top/action/doSearch?ContribAuthorStored=Reid%2C+Chantal+D), Hoye, [K.A.](https://onlinelibrary.wiley.xilesou.top/action/doSearch?ContribAuthorStored=Hoye%2C+Katherine+A), Hart, [E.](https://onlinelibrary.wiley.xilesou.top/action/doSearch?ContribAuthorStored=Hart%2C+Elizabeth) & Jackson, [R.B.](https://onlinelibrary.wiley.xilesou.top/action/doSearch?ContribAuthorStored=Jackson%2C+Robert+B) Elevated CO_2_ reduces disease incidence and severity of a red maple fungal pathogen via changes in host physiology and leaf chemistry. *Glob. Change Biol.* **11,** 1828–1836 (2005).

70. Mo, Q. *et al*. Response of plant nutrient stoichiometry to fertilization varied with plant tissues in a tropical forest. *Sci. Rep.* **5,** 14605 (2015).

71. Murray, T.J., Ellsworth, D.S., Tissue, D.T. & Riegler, M. Interactive direct and plant-mediated effects of elevated atmospheric [CO_2_] and temperature on a eucalypt-feeding insect herbivore. *Glob. Chang Biol.* **19,** 1407-1416 (2013).

72. Norby, R.J., Long, T.M., Hartz-Rubin, J.S. & O'Neill, E.G. Nitrogen resorption in senescing tree leaves in a warmer, CO_2_-enriched atmosephere. *Plant Soil* **224,** 15-29 (2000).

73. Norby, R.J. & Iversen, C.M. (2006) Nitrogen uptake, distribution, turnover, and efficiency of use in a CO_2_-enriched sweetgum forest. *Ecology* **87,** 5–14 (2000).

74. Norby, R.J., Warren, J.M., Iversen, C.M., Medlyn, B.E. & McMurtrie, R.E. CO_2_ enhancement of forest productivity constrained by limited nitrogen availability. *PNAS.* **107,** 19368–19373 (2010).

75. Novriyanti, E. *et al*. High nitrogen and elevated [CO_2_] effects on the growth, defense and photosynthetic performance of two eucalypt species. *Environ. Pollut.* **170,** 124-130 (2012).

76. Nybakken, L., Sandvik, S.M. & Klanderud, K. Experimental warming had little effect on carbon-based secondary compounds, carbon and nitrogen in selected alpine plants and lichens. *Environ. Exp. Bot.* **72,** 368-376 (2011).

77. Oksanen, E., Riikonen, J., Kaakinen, S., Holopainen, T. & Vapaavuori, E. Structural characteristics and chemical composition of birch (*Betula pendula*) leaves are modified by increasing CO_2_ and ozone. *Glob. Change Biol.* **11,** 732-748 (2005).

78. Olsrud, M., Carlsson, B.Å., Svensson, B.M., Michelsen, A. & Melillo, J.M. Responses of fungal root colonization, plant cover and leaf nutrients to long-term exposure to elevated atmospheric CO_2_ and warming in a subarctic birch forest understory. *Glob. Change Biol.* **16,** 1820-1829 (2009).

79. Onoda, Y., Hirose, T. & Hikosaka, K. Effect of elevated CO_2_ levels on leaf starch, nitrogen and photosynthesis of plants growing at three natural CO_2_ springs in Japan. *Ecol. Res.* **22,** 475-484 (2006).

80. Rinnan, R. *et al*. Few long-term effects of simulated climate change on volatile organic compound emissions and leaf chemistry of three subarctic dwarf shrubs. *Environ. Exp. Bot.* **72,** 377-386 (2011).

81. Ren, H. *et al*. Increased precipitation induces a positive plant-soil feedback in a semi-arid grassland. *Plant Soil* **389,** 211–223 (2015).

82. Rodgers, V.L., Hoeppner, S.S., Daley, M.J. & Dukes, J.S. Leaf-Level Gas Exchange and Foliar Chemistry of Common Old-Field Species Responding to Warming and Precipitation Treatments. *Int. J. Plant Sci.* **173,** 957-970 (2012).

83. Sardans, J., Peñuelas, J. & Estiarte, M. Warming and drought alter soil phosphatase activity and soil P availability in a Mediterranean shrubland. *Plant Soil* **289,** 227-238 (2006).

84. Sardans, J., Peñuelas, J. & Ogaya, R. Drought-Induced Changes in C and N Stoichiometry in a *Quercus ilex* Mediterranean Forest. *For. Sci.* **54,** 513–522 (2008).

85. Schaberg, P.G., Perkins, T.D. & McNulty, S.G. Effects of chronic low-level N additions on foliar elemental concentrations, morphology, and gas exchange of mature montane red spruce. *Can. J. For. Res.* **27,** 1622-1629 (1997).

86. Shi, Y. *et al*. Interactive effects of elevated CO_2_ and precipitation change on leaf nitrogen of dominant *Stipa* L. species. *Ecol. Evol.* **5,** 2956-2965 (2015).

87. Shen, Y. *et al*. Increased precipitation modulates the inﬂuence of nitrogen and litter inputs on the nutrient resorption proﬁciency rather than efﬁciency of *Leymus chinensis*. *Plant Ecol.* **219,** 217–230 (2018).

88. Sinclair, T.R. *et al*. Leaf nitrogen concentration of wheat subjected to elevated [CO_2_] and either water or N deﬁcits. *Agricul., Ecosyst. Environ.* **79,** 53–60 (2000).

89. Song, C.J. *et al*. Interactive effects of water, nitrogen and phosphorus on the growth, biomass partitioning and water-use efficiency of Bauhinia faberi seedlings. *J. Arid Environ.* **74,** 1003-1012 (2010).

90. Song, Y., Li, Q., Wang, P., Zhou, D. & Wu, Y. Response of *Leymus chinensis* functional traits and aboveground biomass to nitrogen addition in Songnen grassland in northeast China. *Pratacul. Sci.* **33,** 1383-1390 (2016).

91. Soudzilovskaia, N.A., Onipchenko, V.G., Cornelissen, J.H.C. & Aerts, R. Effects of fertilisation and irrigation on ʻfoliar afterlifeʼ in alpine tundra. *J. Veg. Sci.* **18,** 755-766 (2007).

92. Sun, L.J. & Jiang, C.Y. Effects of different nitrogen concentrations on *Castanopsis fissa* Leaf Characteristics and Root Soil Properties. *Southwest China J. Agricul. Sci.* **29,** 2908-2915 (2016).

93. Suseela, V., Tharayil, N., Xing, B. & Dukes, J.S. Warming and drought differentially influence the production and resorption of elemental and metabolic nitrogen pools in *Quercus rubra*. *Glob. Change Biol.* **21,** 4177-4195 (2015).

94. Tian, Q., Wang, G. & Cao, Z. The response of leaf traits of 14 plant species in typical steppe to the simulated rainfall. *J. Gansu Agricul. University* **5,** 129-134 (2008).

95. Van Heerwaarden, L.M., Toet, S. & Aerts, [R.](https://a.glgoo.top/citations?user=2mKvaHQAAAAJ&hl=zh-CN&oi=sra) Nitrogen and phosphorus resorption efﬁciency and proﬁciency in six sub-arctic bog species after 4 years of nitrogen fertilization. *J. Ecol.* **91,** 1060–1070 (2003b).

96. Veteli, T.O., Kuokkanen, K. & Julkunen‐Tiitto, R. Effects of elevated CO_2_ and temperature on plant growth and herbivore defensive chemistry. *Glob. Change Biol.* **8,** 1240-1252 (2002).

97. Volder, A., Gifford, R.M. & Evans, J.R. Effects of elevated atmospheric CO_2_ concentrations, clipping regimen and differential day/night atmospheric warming on tissue nitrogen concentrations of a perennial pasture grass. *AoB Plants* **7,** 1-15 (2015).

98. Wang, M., Murphy, M.T. & Moore, T.R. Nutrient resorption of two evergreen shrubs in response to longterm fertilization in a bog. *Oecologia* **174,** 365-377 (2014).

99. Wang, X.W., Ji, L.Z., Zhang, Q.H., Liu, Y. & Wang, G.Q. Effects of elevated CO_2_ on feeding preference and performance of the gypsy moth (*Lymantria dispar*) larvae. *J. Appl. Entomol.* **133,** 47-57 (2009).

100. Warren, J.M., Jensen, A.M., Medlyn, B.E., Norby, R.J. & Tissue, D.T. Carbon dioxide stimulation of photosynthesis in *Liquidambar styraciflua* is not sustained during a 12-year field experiment. *AoB Plants* **7** (2014).

101. Welker, J.M., Fahnestock, J.T., Sullivan, P.F. & Chimner, R.A. Leaf mineral nutrition of Arctic plants in response to warming and deeper snow in northern Alaska. *Oikos* **109,** 167-177 (2005).

102. White-Monsant, A.C., Clark, G.J., Chuen, M.A.G. N, K. & Tang, C. Experimental warming and antecedent fire alter leaf element composition and increase soil C:N ratio in sub-alpine open heathland. *Sci. Total Environ.* **595,** 41-50 (2017).

103. Williams, R.S., Lincoln, D.E. & Norby, R.J. Development of gypsy moth larvae feeding on red maple saplings at elevated CO_2_ and temperature. *Oecologia* **137,** 114-122 (2003).

104. Wu, Y., Zheng, X., Yan, L. & Tang, L. Photosynthetic responses and biomass allocation strategies of desert herbaceous plants under different precipitation patterns. *Chin. J. Ecol.* **32,** 2583-2590 (2013).

105. Xiao, D., Wang, X.J., Zhang, K., He, N.P. & Hou, J.H. Effects of nitrogen addition on leaf traits of common species in natural *Pinus tabuliformis* forests in Taiyue Mountain, Shanxi Province, China. *Chin. J. Plant Ecol.* **40,** 686-701 (2016).

106. Xie, X. *et al*. Asymmetric warming effects on N dynamics and productivity in rice (*Oryza sativa* L.). *Soil Sci. Plant Nutr.* **60,** 530-539 (2014).

107. Xu, Z.F., Hu, T.X., Wang, K.Y., Zhang, Y.B. & Xian, J.R. Short-term responses of phenology, shoot growth and leaf traits of four alpine shrubs in a timberline ecotone to simulated global warming, Eastern Tibetan Plateau, China. *Plant Spec. Biol.* **24,** 27-34 (2009).

108. Yang, L., Wang, G., Yang, Y. & Yang, Y. Responses of leaf functional traits and nitrogen and phosphorus stoichiometry in *Abies fabiri* seedlings in Gongga Mountain to simulated nitrogen deposition. *Chin. J. Ecol.* **31,** 44-50 (2012).

109. Yang, [H](https://a.glgoo.top/citations?user=XQMF_okAAAAJ&hl=zh-CN&oi=sra). & Luo, Y. Responses of the functional traits in *Cleistogenes squarrosa* to nitrogen addition and drought. *Chin. J. Plant Ecol.* **39,** 32-42 (2015).

110. Yang, Y., Wang, G., Klanderud, K. & Yang, L. Responses in leaf functional traits and resource allocation of a dominant alpine sedge (*Kobresia pygmaea*) to climate warming in the Qinghai-Tibetan Plateau permafrost region. *Plant Soil* **349,** 377-387 (2011).

111. Yang, Y., Wang, G., Yang, L. & Guo, J. Effects of Drought and Warming on Biomass, Nutrient Allocation, and Oxidative Stress in *Abies fabri* in Eastern Tibetan Plateau. *J. Plant Growth Regul.* **32,** 298-306 (2012).

112. Zhang, Y., Li, R. & Wang, Y. Night-time warming affects N and P dynamics and productivity of winter wheat plants. *Can. J. Plant Sci.* **93,** 397-406 (2013).

113. Zhang, L. *et al*. Effects of Elevated CO2 and N Addition on Growth and N_2_ Fixation of a Legume Subshrub (*Caragana microphylla* Lam.) in Temperate Grassland in China. *PloS one* **6**, e26842 (2011).

114. Zavalloni, C. *et al*. Exposure to warming and CO_2_ enrichment promotes greater above-ground biomass, nitrogen, phosphorus and arbuscular mycorrhizal colonization in newly established grasslands. *Plant Soil* **359,** 121-136 (2012).

115. Zhang, L. *et al*. Responses of a dominant temperate grassland plant (*Leymus chinensis*) to elevated carbon dioxide and nitrogen addition in China. *J. Environ. Qual.* **39,** 251-259 (2010).

116. Zhang, Q. *et al*. Short-term effects of soil warming and nitrogen addition on the N:P stoichiometry of *Cunninghamia lanceolata* in subtropical regions. *Plant Soil* **411,** 395-407 (2016a).

117. Zhang, S., Fu, W., Zhang, Z., Fan, Y. & Liu, T. Effects of elevated CO_2_ concentration and temperature on some physiological characteristics of cotton (*Gossypium hirsutum* L.) leaves. *Environ. Exp. Bot.* **133,** 108-117 (2017).

118. Zhang, T., Yang, S., Guo, R. & Guo, J. Warming and Nitrogen Addition Alter Photosynthetic Pigments, Sugars and Nutrients in a Temperate Meadow Ecosystem. *PloS one* **11**, e0155375 (2016b).

119. Zhao, C., Liang, J., He, J. & Liu, Q. Effects of elevated temperature and nitrogen fertilization on nitrogen metabolism and nutrient status of two coniferous species. *Soil Sci. Plant Nutr.* **58,** 772-782 (2012).

120. Zheng, Y., Xu, M., Shen, R. & Qiu, S. Effects of artificial warming on the structural, physiological, and biochemical changes of maize (*Zea mays* L.) leaves in northern China. *Acta Physiol. Plant.* **35,** 2891-2904 (2013).

121. Zong, N., Shi, P. & Chai, X. Effects of warming and nitrogen addition on nutrient resorption efficiency in an alpine meadow on the northern Tibetan Plateau. *Soil Sci. Plant Nutr.* **64,** 482-490 (2018).

122. Zhao, Q., Liu, X., Hu, Y. & Zeng, D. Effects of Nitrogen Addition on Nutrient Allocation and Nutrient Resorption Efficiency in Larix gmelinii. *Scientia Silvae Sinicae* **46,** 14-19 (2010).

123. Zhuang, M., Li, Y., Guo, Z., Yang, Q. & Chen, H. Effects of elevated CO_2_ on the leaf nutrient stoichiometrical characteristics in *Phyllostachys edulis* and *Oligostachyum lubricum*. *Plant Nutr. Fertil. Sci.* **19,** 239-245 (2013).
