# Supplementary material for: Variations in foliar carbon:nitrogen and nitrogen:phosphorus ratios under global change: a meta-analysis of experimental field studies
Source: Sci Rep. 2020 Jul 22;10:12156. doi: 10.1038/s41598-020-68487-0 (PMC7376191; doi:10.1038/s41598-020-68487-0)
Supplement: Supplementary file 3 — Supplementary Information 3 (DOCX 29 kb) [file 41598_2020_68487_MOESM3_ESM.docx]

phylosignal - with phylometa P<0.1

Jinlong Zhang

2/22/2020

setwd("/Users/jinlong/Desktop/phylometa/")
library(metafor)

## Loading required package: Matrix

## Loading 'metafor' package (version 2.1-0). For an overview
## and introduction to the package please type: help(metafor).

library(plantlist)

## This is plantlist 0.6.1.

library(openxlsx)
library(V.PhyloMaker)

## Loading required package: ape

library(picante)

## Loading required package: vegan

## Loading required package: permute

## Loading required package: lattice

## This is vegan 2.5-6

##
## Attaching package: 'vegan'

## The following object is masked from 'package:metafor':
##
## permutest

## Loading required package: nlme

rm(list = ls())
dat <- read.xlsx("Appendix B20200222.xlsx")
head(dat)

## Code Treatment Variable Reference Sites
## 1 1 Warming foliar C Gherlenda et al 2015 Australia
## 2 2 Warming foliar C Gherlenda et al 2015 Australia
## 3 3 Warming foliar C Hudson et al 2011&#10; Canada
## 4 4 Warming foliar C Hudson et al 2011&#10; Canada
## 5 5 Warming foliar C Hudson et al 2011&#10; Canada
## 6 6 Warming foliar C Hudson et al 2011&#10; Canada
## Species Branch_length Longitude Latitude MAP MAT
## 1 Eucalyptus_robusta 34.5298919999999 150.74 -33.61 900 17.6
## 2 Eucalyptus_tereticornis 34.5298919999999 150.74 -33.61 900 17.6
## 3 Dryas_integrifolia 9.01963200000012 -75.78 78.88 123 -14.4
## 4 Cassiope_digyna 8.185473 -75.78 78.88 123 -14.4
## 5 <NA> <NA> -75.78 78.88 123 -14.4
## 6 Salix_arctica 0.124984 -75.78 78.88 123 -14.4
## Duration Level LnRR
## 1 0.58 4 -0.0202148370572155
## 2 0.58 4 -0.0238044425697222
## 3 16 1.5 0.0307359250082878
## 4 16 1.5 -0.0538502975322379
## 5 16 1.5 -0.029056393683752
## 6 16 1.5 -0.00634177701688674

dat$longlat <- paste(dat$Longitude, dat$Latitude)
dat <- subset(dat, select = c("Treatment", "Variable", "Reference", "Species", "longlat", "LnRR"))

splist <- unique(TPL(gsub("_", " ", na.omit(dat$Species))))$YOUR_SEARCH
dat <- dat[gsub("_", " ", dat$Species) %in% splist,]
dat <- dat[dat$longlat != "NA NA",]

combinations <- unique(data.frame(dat$Treatment, dat$Variable))
dim(combinations)

## [1] 29 2

combinations <- combinations[combinations[,1] != "Treatment",]
dim(combinations)

## [1] 28 2

splist <- unique(dat$Species)

tab2 <- subset(TPL(gsub("_", " ", splist)),
 select = c("YOUR_SEARCH", "POSSIBLE_GENUS", "FAMILY"))
colnames(tab2) <- c("species", "genus", "family")
tree <- phylo.maker(tab2, scenarios = c("S1"))

## [1] "Note: 1 taxa fail to be binded to the tree,"
## [1] "Species"

for (i in 1:nrow(combinations)){

 print(paste("-------------Combination:", i, "---------------"))
 print(combinations[i,])

 temp_dat <- subset(dat, dat$Treatment == combinations[i,1] & dat$Variable == combinations[i,2] )
 temp_dat <- na.omit(temp_dat)
 temp_dat$LnRR <- as.numeric(temp_dat$LnRR)
 temp_splist <- unique(temp_dat$Species)

 tem_phy <- drop.tip(tree[[1]], splist[! splist %in% temp_splist])

 lnrr <- tapply(as.numeric(temp_dat$LnRR), temp_dat$Species, mean)
 lnrr2 <- as.vector(lnrr)
 names(lnrr2) <- names(lnrr)
 matched_dat <- match.phylo.data(phy = multi2di(tem_phy), data = lnrr2)
 res <- phylosignal(matched_dat$data, matched_dat$phy, reps = 9999)

 if(res$PIC.variance.P < 0.1){
 corMatrix <- vcv(matched_dat$phy) #default model="Brownian"

 cor_mat <- as.matrix(corMatrix[match(temp_dat$Species, rownames(corMatrix)), match(temp_dat$Species, colnames(corMatrix))])

 x_mod <- rma.mv(yi = LnRR, V = rep(1, nrow(temp_dat)),
 random = list(~1|Species),
 R = list(Species = cor_mat),
 Rscale = "cov0",
 method="REML",
 verbose = FALSE,
 data = temp_dat)
 print(summary(x_mod))
 }

 print(" ")
 print("List of species:")
 print(sort(unique(temp_dat$Species)))
 print(" ")
 print("Number of species:")
 print(length(unique(temp_dat$Species)))
 print(" ")
 print("Result of Phylogenetic Signal:")
 print(res)
 print(" ")
 print("Saving phylogenetic tree")
 jpeg(paste(combinations[i,1],"tree.jpg", collapse = "_"), width = 3600, height = 3600, res = 300)
 plot(matched_dat$phy, type = "f")
 dev.off()
 print(" ")
 print("--------------End of this combination----------------")
 print(" ")
 print(" ")
}

## [1] "-------------Combination: 1 ---------------"
## dat.Treatment dat.Variable
## 1 Warming foliar C
##
## Multivariate Meta-Analysis Model (k = 47; method: REML)
##
## logLik Deviance AIC BIC AICc
## -42.3313 84.6625 88.6625 92.3198 88.9416
##
## Variance Components:
##
## estim sqrt nlvls fixed factor R
## sigma^2 0.0000 0.0000 42 no Species yes
##
## Test for Heterogeneity:
## Q(df = 46) = 0.1202, p-val = 1.0000
##
## Model Results:
##
## estimate se zval pval ci.lb ci.ub
## -0.0057 0.1459 -0.0388 0.9691 -0.2915 0.2802
##
## ---
## Signif. codes: 0 '***' 0.001 '**' 0.01 '*' 0.05 '.' 0.1 ' ' 1
##
## [1] " "
## [1] "List of species:"
## [1] "Abies_fabri" "Arctostaphylos_alpina"
## [3] "Asterolasia_trymalioides" "Betula_nana"
## [5] "Carex_atrofusca" "Carex_breviculmis"
## [7] "Carex_vaginata" "Cassiope_digyna"
## [9] "Celmisia_pugioniformis" "Craspedia_jamesii"
## [11] "Dorycnium_pentaphyllum" "Dryas_integrifolia"
## [13] "Dryas_octopetala" "Erica_multiflora"
## [15] "Eriophorum_vaginatum" "Eucalyptus_robusta"
## [17] "Eucalyptus_tereticornis" "Globularia_alypum"
## [19] "Kobresia_pygmaea" "Kobresia_tibetica"
## [21] "Ledum_palustre" "Leymus_chinensis"
## [23] "Lolium_perenne" "Lotus_corniculatus"
## [25] "Medicago_lupulina" "Oxyria_tetragona"
## [27] "Phragmites_communis" "Plantago_lanceolata"
## [29] "Poa_hiemata" "Poa_pratensis"
## [31] "Ranunculus_victoriensis" "Rhododendron_aureum"
## [33] "Rumex_acetosa" "Rytidosperma_nudiflorum"
## [35] "Salix_arctica" "Salix_pulchra"
## [37] "Saussurea_alpina" "Selaginella_selaginoides"
## [39] "Tofieldia_pusilla" "Vaccinium_uliginosum"
## [41] "Vaccinium_vitis-idaea" "Zea_mays"
## [1] " "
## [1] "Number of species:"
## [1] 42
## [1] " "
## [1] "Result of Phylogenetic Signal:"
## K PIC.variance.obs PIC.variance.rnd.mean PIC.variance.P
## 1 0.1206073 0.0001024697 0.0008556158 0.0864
## PIC.variance.Z
## 1 -0.4245283
## [1] " "
## [1] "Saving phylogenetic tree"

## [1] " "
## [1] "--------------End of this combination----------------"
## [1] " "
## [1] " "
## [1] "-------------Combination: 2 ---------------"
## dat.Treatment dat.Variable
## 49 Warming foliar N
## [1] " "
## [1] "List of species:"
## [1] "Abies_fabri" "Acer_rubrum"
## [3] "Acer_saccharum" "Allium_atrosanguineum"
## [5] "Andromeda_polifolia" "Arctagrostis_latifolia"
## [7] "Arctostaphylos_alpina" "Artemisia_frigida"
## [9] "Asterolasia_trymalioides" "Betula_nana"
## [11] "Betula_pendula" "Calamagrostis_lapponica"
## [13] "Carex_atrofusca" "Carex_breviculmis"
## [15] "Carex_vaginata" "Cassiope_digyna"
## [17] "Cassiope_tetragona" "Celmisia_pugioniformis"
## [19] "Cleistogenes_songorica" "Convolvulus_ammannii"
## [21] "Craspedia_jamesii" "Cunninghamia_lanceolata"
## [23] "Daphne_retusa" "Dorycnium_pentaphyllum"
## [25] "Dryas_integrifolia" "Dryas_octopetala"
## [27] "Dupontia_fisheri" "Elymus_nutans"
## [29] "Empetrum_hermaphroditum" "Erica_multiflora"
## [31] "Eriophorum_scheuchzeri" "Eriophorum_vaginatum"
## [33] "Eucalyptus_robusta" "Eucalyptus_tereticornis"
## [35] "Globularia_alypum" "Gossypium_hirsutum"
## [37] "Helianthemum_squamatum" "Kobresia_pygmaea"
## [39] "Kobresia_tibetica" "Kochia_prostrata"
## [41] "Koeleria_macrantha" "Ledum_palustre"
## [43] "Leymus_chinensis" "Lolium_perenne"
## [45] "Lotus_corniculatus" "Luzula_sp."
## [47] "Medicago_lupulina" "Oryza_sativa"
## [49] "Oxyria_tetragona" "Phragmites_communis"
## [51] "Picea_asperata" "Plantago_lanceolata"
## [53] "Poa_hiemata" "Poa_pratensis"
## [55] "Potentilla_fruticosa" "Quercus_sp."
## [57] "Ranunculus_victoriensis" "Rhododendron_aureum"
## [59] "Rubus_chamaemorus" "Rumex_acetosa"
## [61] "Rytidosperma_nudiflorum" "Salix_arctica"
## [63] "Salix_myrsinifolia" "Salix_phylicifolia"
## [65] "Salix_pulchra" "Saussurea_alpina"
## [67] "Selaginella_selaginoides" "Spiraea_mongolica"
## [69] "Stipa_breviflora" "Tofieldia_pusilla"
## [71] "Vaccinium_myrtillus" "Vaccinium_uliginosum"
## [73] "Vaccinium_vitis-idaea" "Vicia_unijuga"
## [75] "Zea_mays"
## [1] " "
## [1] "Number of species:"
## [1] 75
## [1] " "
## [1] "Result of Phylogenetic Signal:"
## K PIC.variance.obs PIC.variance.rnd.mean PIC.variance.P
## 1 0.02674778 0.002814619 0.004047979 0.4774
## PIC.variance.Z
## 1 -0.3465966
## [1] " "
## [1] "Saving phylogenetic tree"

## [1] " "
## [1] "--------------End of this combination----------------"
## [1] " "
## [1] " "
## [1] "-------------Combination: 3 ---------------"
## dat.Treatment dat.Variable
## 146 Warming foliar P
## [1] " "
## [1] "List of species:"
## [1] "Abies_fabri" "Arctostaphylos_alpina"
## [3] "Asterolasia_trymalioides" "Betula_nana"
## [5] "Carex_atrofusca" "Carex_breviculmis"
## [7] "Cassiope_tetragona" "Celmisia_pugioniformis"
## [9] "Craspedia_jamesii" "Cunninghamia_lanceolata"
## [11] "Dryas_octopetala" "Elymus_nutans"
## [13] "Erica_multiflora" "Eriophorum_vaginatum"
## [15] "Globularia_alypum" "Helianthemum_squamatum"
## [17] "Kobresia_pygmaea" "Kobresia_tibetica"
## [19] "Leymus_chinensis" "Lolium_perenne"
## [21] "Lotus_corniculatus" "Medicago_lupulina"
## [23] "Phragmites_communis" "Plantago_lanceolata"
## [25] "Poa_hiemata" "Poa_pratensis"
## [27] "Ranunculus_victoriensis" "Rhododendron_aureum"
## [29] "Rumex_acetosa" "Rytidosperma_nudiflorum"
## [31] "Salix_myrsinifolia" "Salix_phylicifolia"
## [33] "Salix_pulchra" "Vaccinium_myrtillus"
## [35] "Vaccinium_uliginosum" "Vaccinium_vitis-idaea"
## [37] "Vicia_unijuga" "Zea_mays"
## [1] " "
## [1] "Number of species:"
## [1] 38
## [1] " "
## [1] "Result of Phylogenetic Signal:"
## K PIC.variance.obs PIC.variance.rnd.mean PIC.variance.P
## 1 0.08346434 0.04011107 0.08086681 0.314
## PIC.variance.Z
## 1 -0.6403761
## [1] " "
## [1] "Saving phylogenetic tree"

## [1] " "
## [1] "--------------End of this combination----------------"
## [1] " "
## [1] " "
## [1] "-------------Combination: 4 ---------------"
## dat.Treatment dat.Variable
## 189 Warming foliar CN
## [1] " "
## [1] "List of species:"
## [1] "Abies_fabri" "Arctostaphylos_alpina"
## [3] "Asterolasia_trymalioides" "Betula_nana"
## [5] "Carex_atrofusca" "Carex_breviculmis"
## [7] "Carex_vaginata" "Cassiope_digyna"
## [9] "Celmisia_pugioniformis" "Craspedia_jamesii"
## [11] "Dorycnium_pentaphyllum" "Dryas_integrifolia"
## [13] "Dryas_octopetala" "Erica_multiflora"
## [15] "Eriophorum_vaginatum" "Eucalyptus_robusta"
## [17] "Eucalyptus_tereticornis" "Globularia_alypum"
## [19] "Kobresia_pygmaea" "Kobresia_tibetica"
## [21] "Ledum_palustre" "Leymus_chinensis"
## [23] "Lolium_perenne" "Lotus_corniculatus"
## [25] "Medicago_lupulina" "Oxyria_tetragona"
## [27] "Phragmites_communis" "Plantago_lanceolata"
## [29] "Poa_hiemata" "Poa_pratensis"
## [31] "Ranunculus_victoriensis" "Rhododendron_aureum"
## [33] "Rumex_acetosa" "Rytidosperma_nudiflorum"
## [35] "Salix_arctica" "Salix_pulchra"
## [37] "Saussurea_alpina" "Selaginella_selaginoides"
## [39] "Tofieldia_pusilla" "Vaccinium_uliginosum"
## [41] "Vaccinium_vitis-idaea" "Zea_mays"
## [1] " "
## [1] "Number of species:"
## [1] 42
## [1] " "
## [1] "Result of Phylogenetic Signal:"
## K PIC.variance.obs PIC.variance.rnd.mean PIC.variance.P
## 1 0.02507359 0.005145662 0.009221532 0.4197
## PIC.variance.Z
## 1 -0.4478011
## [1] " "
## [1] "Saving phylogenetic tree"

## [1] " "
## [1] "--------------End of this combination----------------"
## [1] " "
## [1] " "
## [1] "-------------Combination: 5 ---------------"
## dat.Treatment dat.Variable
## 237 Warming foliar NP
##
## Multivariate Meta-Analysis Model (k = 32; method: REML)
##
## logLik Deviance AIC BIC AICc
## -30.0283 60.0566 64.0566 66.9246 64.4852
##
## Variance Components:
##
## estim sqrt nlvls fixed factor R
## sigma^2 0.0000 0.0000 31 no Species yes
##
## Test for Heterogeneity:
## Q(df = 31) = 3.0824, p-val = 1.0000
##
## Model Results:
##
## estimate se zval pval ci.lb ci.ub
## -0.0832 0.1768 -0.4707 0.6379 -0.4297 0.2633
##
## ---
## Signif. codes: 0 '***' 0.001 '**' 0.01 '*' 0.05 '.' 0.1 ' ' 1
##
## [1] " "
## [1] "List of species:"
## [1] "Abies_fabri" "Asterolasia_trymalioides"
## [3] "Betula_nana" "Carex_atrofusca"
## [5] "Carex_breviculmis" "Cassiope_tetragona"
## [7] "Celmisia_pugioniformis" "Craspedia_jamesii"
## [9] "Cunninghamia_lanceolata" "Erica_multiflora"
## [11] "Globularia_alypum" "Helianthemum_squamatum"
## [13] "Kobresia_pygmaea" "Kobresia_tibetica"
## [15] "Leymus_chinensis" "Lolium_perenne"
## [17] "Lotus_corniculatus" "Medicago_lupulina"
## [19] "Phragmites_communis" "Plantago_lanceolata"
## [21] "Poa_hiemata" "Poa_pratensis"
## [23] "Ranunculus_victoriensis" "Rhododendron_aureum"
## [25] "Rumex_acetosa" "Rytidosperma_nudiflorum"
## [27] "Salix_phylicifolia" "Vaccinium_myrtillus"
## [29] "Vaccinium_uliginosum" "Vaccinium_vitis-idaea"
## [31] "Zea_mays"
## [1] " "
## [1] "Number of species:"
## [1] 31
## [1] " "
## [1] "Result of Phylogenetic Signal:"
## K PIC.variance.obs PIC.variance.rnd.mean PIC.variance.P
## 1 0.2193035 0.00239984 0.01159904 0.0268
## PIC.variance.Z
## 1 -0.7130188
## [1] " "
## [1] "Saving phylogenetic tree"

## [1] " "
## [1] "--------------End of this combination----------------"
## [1] " "
## [1] " "
## [1] "-------------Combination: 6 ---------------"
## dat.Treatment dat.Variable
## 270 Warming NRE
## [1] " "
## [1] "List of species:"
## [1] "Acer_rubrum" "Acer_saccharum"
## [3] "Ambrosia_psilostachyia" "Anaphalis_xylorhiza"
## [5] "Artemisia_frigida" "Aster_ericoides"
## [7] "Betula_nana" "Calamagrostis_lapponica"
## [9] "Cleistogenes_songorica" "Convolvulus_ammannii"
## [11] "Cunninghamia_lanceolata" "Dichanthelium_oligosnathes"
## [13] "Kobresia_pygmaea" "Kochia_prostrata"
## [15] "Rubus_chamaemorus" "Schizachyrium_scoparium"
## [17] "Sporobolus_asper" "Stipa_breviflora"
## [19] "Stipa_capillacea" "Vaccinium_uliginosum"
## [1] " "
## [1] "Number of species:"
## [1] 20
## [1] " "
## [1] "Result of Phylogenetic Signal:"
## K PIC.variance.obs PIC.variance.rnd.mean PIC.variance.P
## 1 0.1157217 0.001898944 0.001428617 0.7935
## PIC.variance.Z
## 1 0.5716539
## [1] " "
## [1] "Saving phylogenetic tree"

## [1] " "
## [1] "--------------End of this combination----------------"
## [1] " "
## [1] " "
## [1] "-------------Combination: 7 ---------------"
## dat.Treatment dat.Variable
## 291 Warming PRE
## [1] " "
## [1] "List of species:"
## [1] "Anaphalis_xylorhiza" "Cunninghamia_lanceolata"
## [3] "Kobresia_pygmaea" "Stipa_capillacea"
## [1] " "
## [1] "Number of species:"
## [1] 4
## [1] " "
## [1] "Result of Phylogenetic Signal:"
## K PIC.variance.obs PIC.variance.rnd.mean PIC.variance.P
## 1 0.3875128 0.0002651454 0.00016671 0.89445
## PIC.variance.Z
## 1 1.610529
## [1] " "
## [1] "Saving phylogenetic tree"

## [1] " "
## [1] "--------------End of this combination----------------"
## [1] " "
## [1] " "
## [1] "-------------Combination: 8 ---------------"
## dat.Treatment dat.Variable
## 296 Increased precipitation foliar C
## [1] " "
## [1] "List of species:"
## [1] "Atriplex_patens" "Kochia_scoparia" "Salicornia_europaea"
## [4] "Salsola_lanata"
## [1] " "
## [1] "Number of species:"
## [1] 4
## [1] " "
## [1] "Result of Phylogenetic Signal:"
## K PIC.variance.obs PIC.variance.rnd.mean PIC.variance.P PIC.variance.Z
## 1 1.108252 0.0001905818 0.0002249203 0.10345 -0.9026561
## [1] " "
## [1] "Saving phylogenetic tree"

## [1] " "
## [1] "--------------End of this combination----------------"
## [1] " "
## [1] " "
## [1] "-------------Combination: 9 ---------------"
## dat.Treatment dat.Variable
## 305 Increased precipitation foliar N
##
## Multivariate Meta-Analysis Model (k = 35; method: REML)
##
## logLik Deviance AIC BIC AICc
## -31.8993 63.7986 67.7986 70.8514 68.1857
##
## Variance Components:
##
## estim sqrt nlvls fixed factor R
## sigma^2 0.0000 0.0000 24 no Species yes
##
## Test for Heterogeneity:
## Q(df = 34) = 1.3108, p-val = 1.0000
##
## Model Results:
##
## estimate se zval pval ci.lb ci.ub
## -0.0437 0.1690 -0.2587 0.7959 -0.3750 0.2876
##
## ---
## Signif. codes: 0 '***' 0.001 '**' 0.01 '*' 0.05 '.' 0.1 ' ' 1
##
## [1] " "
## [1] "List of species:"
## [1] "Agropyron_cristatum" "Artemisia_frigida"
## [3] "Astragalus_scaberrimus" "Atriplex_patens"
## [5] "Bauhinia_faberi" "Bromus_rubens"
## [7] "Carduus_tenuifolius" "Cleistogenes_squarrosa"
## [9] "Heteropappus_altaicus" "Iris_ensata"
## [11] "Kochia_scoparia" "Medicago_ruthenica"
## [13] "Melilotus_officinalis" "Oxytropis_microphylla"
## [15] "Phlomis_umbrosa" "Pinus_taeda"
## [17] "Poa_annua" "Potentilla_acaulis"
## [19] "Potentilla_bifurca" "Potentilla_chinensis"
## [21] "Potentilla_tanacetifolia" "Salicornia_europaea"
## [23] "Salsola_lanata" "Stipa_krylovii"
## [1] " "
## [1] "Number of species:"
## [1] 24
## [1] " "
## [1] "Result of Phylogenetic Signal:"
## K PIC.variance.obs PIC.variance.rnd.mean PIC.variance.P
## 1 0.3321451 0.0004831738 0.0009026582 0.0216
## PIC.variance.Z
## 1 -1.56245
## [1] " "
## [1] "Saving phylogenetic tree"

## [1] " "
## [1] "--------------End of this combination----------------"
## [1] " "
## [1] " "
## [1] "-------------Combination: 10 ---------------"
## dat.Treatment dat.Variable
## 341 Increased precipitation foliar P
## [1] " "
## [1] "List of species:"
## [1] "Agropyron_cristatum" "Atriplex_patens"
## [3] "Bromus_rubens" "Carduus_tenuifolius"
## [5] "Cleistogenes_squarrosa" "Kochia_scoparia"
## [7] "Medicago_ruthenica" "Melilotus_officinalis"
## [9] "Potentilla_tanacetifolia" "Salicornia_europaea"
## [11] "Salsola_lanata" "Stipa_krylovii"
## [1] " "
## [1] "Number of species:"
## [1] 12
## [1] " "
## [1] "Result of Phylogenetic Signal:"
## K PIC.variance.obs PIC.variance.rnd.mean PIC.variance.P
## 1 0.4914217 0.0004980762 0.0008308188 0.1126
## PIC.variance.Z
## 1 -1.046977
## [1] " "
## [1] "Saving phylogenetic tree"

## [1] " "
## [1] "--------------End of this combination----------------"
## [1] " "
## [1] " "
## [1] "-------------Combination: 11 ---------------"
## dat.Treatment dat.Variable
## 358 Increased precipitation foliar CN
## [1] " "
## [1] "List of species:"
## [1] "Atriplex_patens" "Kochia_scoparia" "Salicornia_europaea"
## [4] "Salsola_lanata"
## [1] " "
## [1] "Number of species:"
## [1] 4
## [1] " "
## [1] "Result of Phylogenetic Signal:"
## K PIC.variance.obs PIC.variance.rnd.mean PIC.variance.P
## 1 0.8789174 0.0004097412 0.0003969975 0.6089
## PIC.variance.Z
## 1 0.1678933
## [1] " "
## [1] "Saving phylogenetic tree"

## [1] " "
## [1] "--------------End of this combination----------------"
## [1] " "
## [1] " "
## [1] "-------------Combination: 12 ---------------"
## dat.Treatment dat.Variable
## 367 Increased precipitation foliar NP
## [1] " "
## [1] "List of species:"
## [1] "Agropyron_cristatum" "Atriplex_patens"
## [3] "Bromus_rubens" "Carduus_tenuifolius"
## [5] "Cleistogenes_squarrosa" "Kochia_scoparia"
## [7] "Medicago_ruthenica" "Melilotus_officinalis"
## [9] "Potentilla_tanacetifolia" "Salicornia_europaea"
## [11] "Salsola_lanata" "Stipa_krylovii"
## [1] " "
## [1] "Number of species:"
## [1] 12
## [1] " "
## [1] "Result of Phylogenetic Signal:"
## K PIC.variance.obs PIC.variance.rnd.mean PIC.variance.P
## 1 0.1868429 0.001271379 0.0008119789 0.9457
## PIC.variance.Z
## 1 1.704219
## [1] " "
## [1] "Saving phylogenetic tree"

## [1] " "
## [1] "--------------End of this combination----------------"
## [1] " "
## [1] " "
## [1] "-------------Combination: 13 ---------------"
## dat.Treatment dat.Variable
## 384 Increased precipitation NRE
## [1] " "
## [1] "List of species:"
## [1] "Agropyron_cristatum" "Anemone_speciosa"
## [3] "Carex_caryophyllea" "Carex_sempervirens"
## [5] "Cleistogenes_squarrosa" "Festuca_ovina"
## [7] "Medicago_ruthenica" "Polygonum_bistorta"
## [9] "Potentilla_tanacetifolia" "Stipa_krylovii"
## [11] "Trifolium_polyphyllum"
## [1] " "
## [1] "Number of species:"
## [1] 11
## [1] " "
## [1] "Result of Phylogenetic Signal:"
## K PIC.variance.obs PIC.variance.rnd.mean PIC.variance.P
## 1 0.09822227 0.001294315 0.0006478258 0.9066
## PIC.variance.Z
## 1 1.388867
## [1] " "
## [1] "Saving phylogenetic tree"

## [1] " "
## [1] "--------------End of this combination----------------"
## [1] " "
## [1] " "
## [1] "-------------Combination: 14 ---------------"
## dat.Treatment dat.Variable
## 396 Increased precipitation PRE
## [1] " "
## [1] "List of species:"
## [1] "Agropyron_cristatum" "Carex_sempervirens"
## [3] "Cleistogenes_squarrosa" "Medicago_ruthenica"
## [5] "Polygonum_bistorta" "Potentilla_tanacetifolia"
## [7] "Stipa_krylovii"
## [1] " "
## [1] "Number of species:"
## [1] 7
## [1] " "
## [1] "Result of Phylogenetic Signal:"
## K PIC.variance.obs PIC.variance.rnd.mean PIC.variance.P
## 1 0.6941624 0.0009957817 0.001273967 0.5212
## PIC.variance.Z
## 1 -0.4173418
## [1] " "
## [1] "Saving phylogenetic tree"

## [1] " "
## [1] "--------------End of this combination----------------"
## [1] " "
## [1] " "
## [1] "-------------Combination: 15 ---------------"
## dat.Treatment dat.Variable
## 404 N addition foliar C
## [1] " "
## [1] "List of species:"
## [1] "Acer_ginnala" "Anemone_rivularis"
## [3] "Anemone_tomentosa" "Betula_pendula"
## [5] "Bupleurum_scorzonerifolium" "Carex_callitrichos"
## [7] "Chamaedaphne_calyculata" "Cornus_bretschneideri"
## [9] "Corylus_mandshurica" "Deyeuxia_angustifolia"
## [11] "Diarrhena_mandshurica" "Elymus_nutans"
## [13] "Glyceria_spiculosa" "Kobresia_myosuroides"
## [15] "Larix_gmelinii" "Ledum_groenlandicum"
## [17] "Leymus_chinensis" "Lonicera_maackii"
## [19] "Oxytropis_ochrocephala" "Pedicularis_kansuensis"
## [21] "Phragmites_communis" "Pinus_tabulaeformis"
## [23] "Polygonatum_odoratum" "Potentilla_ragarioides"
## [25] "Quercus_mongolica" "Spiraea_salicifolia"
## [27] "Stipa_baicalensis" "Thermopsis_lanceolata"
## [29] "Vaccinium_myrilloides"
## [1] " "
## [1] "Number of species:"
## [1] 29
## [1] " "
## [1] "Result of Phylogenetic Signal:"
## K PIC.variance.obs PIC.variance.rnd.mean PIC.variance.P
## 1 0.3245267 0.0005297887 0.0006447396 0.4476
## PIC.variance.Z
## 1 -0.329004
## [1] " "
## [1] "Saving phylogenetic tree"

## [1] " "
## [1] "--------------End of this combination----------------"
## [1] " "
## [1] " "
## [1] "-------------Combination: 16 ---------------"
## dat.Treatment dat.Variable
## 483 N addition foliar N
##
## Multivariate Meta-Analysis Model (k = 183; method: REML)
##
## logLik Deviance AIC BIC AICc
## -170.7611 341.5223 345.5223 351.9303 345.5893
##
## Variance Components:
##
## estim sqrt nlvls fixed factor R
## sigma^2 0.0000 0.0000 74 no Species yes
##
## Test for Heterogeneity:
## Q(df = 182) = 7.0287, p-val = 1.0000
##
## Model Results:
##
## estimate se zval pval ci.lb ci.ub
## 0.1759 0.0739 2.3792 0.0173 0.0310 0.3208 *
##
## ---
## Signif. codes: 0 '***' 0.001 '**' 0.01 '*' 0.05 '.' 0.1 ' ' 1
##
## [1] " "
## [1] "List of species:"
## [1] "Abies_fabri" "Acer_ginnala"
## [3] "Acmena_acuminatissima" "Agropyron_cristatum"
## [5] "Andromeda_polifolia" "Anemone_rivularis"
## [7] "Anemone_tomentosa" "Artemisia_frigida"
## [9] "Betula_nana" "Betula_pendula"
## [11] "Bupleurum_scorzonerifolium" "Camellia_cuspidata"
## [13] "Caragana_microphylla" "Carex_callitrichos"
## [15] "Cassiope_tetragona" "Castanopsis_fissa"
## [17] "Castanopsis_hystrix" "Chamaedaphne_calyculata"
## [19] "Cleistogenes_songorica" "Cleistogenes_squarrosa"
## [21] "Convolvulus_ammannii" "Cornus_bretschneideri"
## [23] "Corylus_mandshurica" "Cryptocarya_chinensis"
## [25] "Cryptocarya_concinna" "Cunninghamia_lanceolata"
## [27] "Deyeuxia_angustifolia" "Diarrhena_mandshurica"
## [29] "Elymus_nutans" "Empetrum_hermaphroditum"
## [31] "Erica_multiflora" "Eriophorum_vaginatum"
## [33] "Eurya_muricata" "Eurya_rubiginosa_var._attenuata"
## [35] "Glyceria_spiculosa" "Kobresia_myosuroides"
## [37] "Kochia_prostrata" "Larix_gmelinii"
## [39] "Ledum_groenlandicum" "Leymus_chinensis"
## [41] "Lonicera_maackii" "Medicago_ruthenica"
## [43] "Melilotoides_ruthenica" "Ormosia_pinnata"
## [45] "Oxytropis_kansuensis" "Oxytropis_ochrocephala"
## [47] "Pasania_sieboldiana" "Pedicularis_kansuensis"
## [49] "Phragmites_communis" "Picea_abies"
## [51] "Picea_asperata" "Picea_glauca"
## [53] "Pinus_tabulaeformis" "Pinus_taeda"
## [55] "Polygonatum_odoratum" "Populus_nigra"
## [57] "Potentilla_fruticosa" "Potentilla_ragarioides"
## [59] "Potentilla_tanacetifolia" "Quercus_mongolica"
## [61] "Randia_canthioides" "Rhododendron_ovatum"
## [63] "Rubus_chamaemorus" "Salix_phylicifolia"
## [65] "Schima_superba" "Spiraea_salicifolia"
## [67] "Stipa_baicalensis" "Stipa_breviflora"
## [69] "Stipa_krylovii" "Symplocos_sumuntia"
## [71] "Syzygium_hancei" "Thermopsis_lanceolata"
## [73] "Vaccinium_myrilloides" "Vaccinium_uliginosum"
## [1] " "
## [1] "Number of species:"
## [1] 74
## [1] " "
## [1] "Result of Phylogenetic Signal:"
## K PIC.variance.obs PIC.variance.rnd.mean PIC.variance.P
## 1 0.1289172 0.001587649 0.003498772 0.0714
## PIC.variance.Z
## 1 -0.7880597
## [1] " "
## [1] "Saving phylogenetic tree"

## [1] " "
## [1] "--------------End of this combination----------------"
## [1] " "
## [1] " "
## [1] "-------------Combination: 17 ---------------"
## dat.Treatment dat.Variable
## 667 N addition foliar P
##
## Multivariate Meta-Analysis Model (k = 158; method: REML)
##
## logLik Deviance AIC BIC AICc
## -150.7076 301.4153 305.4153 311.5278 305.4932
##
## Variance Components:
##
## estim sqrt nlvls fixed factor R
## sigma^2 0.0000 0.0000 61 no Species yes
##
## Test for Heterogeneity:
## Q(df = 157) = 12.8686, p-val = 1.0000
##
## Model Results:
##
## estimate se zval pval ci.lb ci.ub
## 0.0742 0.0796 0.9328 0.3509 -0.0817 0.2301
##
## ---
## Signif. codes: 0 '***' 0.001 '**' 0.01 '*' 0.05 '.' 0.1 ' ' 1
##
## [1] " "
## [1] "List of species:"
## [1] "Abies_fabri" "Acer_ginnala"
## [3] "Acmena_acuminatissima" "Agropyron_cristatum"
## [5] "Andromeda_polifolia" "Anemone_rivularis"
## [7] "Anemone_tomentosa" "Betula_nana"
## [9] "Camellia_cuspidata" "Canthium_horridum"
## [11] "Carallia_brachiata" "Carex_callitrichos"
## [13] "Cassiope_tetragona" "Castanopsis_fissa"
## [15] "Castanopsis_hystrix" "Cleistogenes_squarrosa"
## [17] "Cornus_bretschneideri" "Corylus_mandshurica"
## [19] "Cryptocarya_chinensis" "Cryptocarya_concinna"
## [21] "Deyeuxia_angustifolia" "Diarrhena_mandshurica"
## [23] "Elymus_nutans" "Empetrum_hermaphroditum"
## [25] "Eriophorum_vaginatum" "Eurya_muricata"
## [27] "Eurya_rubiginosa_var._attenuata" "Glyceria_spiculosa"
## [29] "Kobresia_myosuroides" "Larix_gmelinii"
## [31] "Leymus_chinensis" "Lonicera_maackii"
## [33] "Medicago_ruthenica" "Melilotoides_ruthenica"
## [35] "Ormosia_pinnata" "Oxytropis_kansuensis"
## [37] "Oxytropis_ochrocephala" "Pasania_sieboldiana"
## [39] "Pedicularis_kansuensis" "Phragmites_communis"
## [41] "Picea_asperata" "Pinus_tabulaeformis"
## [43] "Polygonatum_odoratum" "Potentilla_fruticosa"
## [45] "Potentilla_ragarioides" "Potentilla_tanacetifolia"
## [47] "Psychotria_rubra" "Quercus_mongolica"
## [49] "Randia_canthioides" "Rhododendron_ovatum"
## [51] "Rubus_chamaemorus" "Salix_phylicifolia"
## [53] "Schefflera_octophylla" "Schima_superba"
## [55] "Spiraea_salicifolia" "Stipa_krylovii"
## [57] "Symplocos_sumuntia" "Syzygium_bullockii"
## [59] "Syzygium_hancei" "Syzygium_levinei"
## [61] "Vaccinium_uliginosum"
## [1] " "
## [1] "Number of species:"
## [1] 61
## [1] " "
## [1] "Result of Phylogenetic Signal:"
## K PIC.variance.obs PIC.variance.rnd.mean PIC.variance.P
## 1 0.1321983 0.002926649 0.01052719 0.066
## PIC.variance.Z
## 1 -0.7195973
## [1] " "
## [1] "Saving phylogenetic tree"

## [1] " "
## [1] "--------------End of this combination----------------"
## [1] " "
## [1] " "
## [1] "-------------Combination: 18 ---------------"
## dat.Treatment dat.Variable
## 826 N addition foliar CN
## [1] " "
## [1] "List of species:"
## [1] "Acer_ginnala" "Anemone_rivularis"
## [3] "Anemone_tomentosa" "Betula_pendula"
## [5] "Bupleurum_scorzonerifolium" "Carex_callitrichos"
## [7] "Chamaedaphne_calyculata" "Cornus_bretschneideri"
## [9] "Corylus_mandshurica" "Deyeuxia_angustifolia"
## [11] "Diarrhena_mandshurica" "Elymus_nutans"
## [13] "Glyceria_spiculosa" "Kobresia_myosuroides"
## [15] "Larix_gmelinii" "Ledum_groenlandicum"
## [17] "Leymus_chinensis" "Lonicera_maackii"
## [19] "Oxytropis_ochrocephala" "Pedicularis_kansuensis"
## [21] "Phragmites_communis" "Pinus_tabulaeformis"
## [23] "Polygonatum_odoratum" "Potentilla_ragarioides"
## [25] "Quercus_mongolica" "Spiraea_salicifolia"
## [27] "Stipa_baicalensis" "Thermopsis_lanceolata"
## [29] "Vaccinium_myrilloides"
## [1] " "
## [1] "Number of species:"
## [1] 29
## [1] " "
## [1] "Result of Phylogenetic Signal:"
## K PIC.variance.obs PIC.variance.rnd.mean PIC.variance.P
## 1 0.1673538 0.0009051467 0.0009341873 0.5363
## PIC.variance.Z
## 1 -0.08248061
## [1] " "
## [1] "Saving phylogenetic tree"

## [1] " "
## [1] "--------------End of this combination----------------"
## [1] " "
## [1] " "
## [1] "-------------Combination: 19 ---------------"
## dat.Treatment dat.Variable
## 905 N addition foliar NP
##
## Multivariate Meta-Analysis Model (k = 158; method: REML)
##
## logLik Deviance AIC BIC AICc
## -153.9463 307.8926 311.8926 318.0051 311.9705
##
## Variance Components:
##
## estim sqrt nlvls fixed factor R
## sigma^2 0.0000 0.0000 61 no Species yes
##
## Test for Heterogeneity:
## Q(df = 157) = 19.3459, p-val = 1.0000
##
## Model Results:
##
## estimate se zval pval ci.lb ci.ub
## 0.0923 0.0796 1.1608 0.2457 -0.0636 0.2483
##
## ---
## Signif. codes: 0 '***' 0.001 '**' 0.01 '*' 0.05 '.' 0.1 ' ' 1
##
## [1] " "
## [1] "List of species:"
## [1] "Abies_fabri" "Acer_ginnala"
## [3] "Acmena_acuminatissima" "Agropyron_cristatum"
## [5] "Andromeda_polifolia" "Anemone_rivularis"
## [7] "Anemone_tomentosa" "Betula_nana"
## [9] "Camellia_cuspidata" "Canthium_horridum"
## [11] "Carallia_brachiata" "Carex_callitrichos"
## [13] "Cassiope_tetragona" "Castanopsis_fissa"
## [15] "Castanopsis_hystrix" "Cleistogenes_squarrosa"
## [17] "Cornus_bretschneideri" "Corylus_mandshurica"
## [19] "Cryptocarya_chinensis" "Cryptocarya_concinna"
## [21] "Deyeuxia_angustifolia" "Diarrhena_mandshurica"
## [23] "Elymus_nutans" "Empetrum_hermaphroditum"
## [25] "Eriophorum_vaginatum" "Eurya_muricata"
## [27] "Eurya_rubiginosa_var._attenuata" "Glyceria_spiculosa"
## [29] "Kobresia_myosuroides" "Larix_gmelinii"
## [31] "Leymus_chinensis" "Lonicera_maackii"
## [33] "Medicago_ruthenica" "Melilotoides_ruthenica"
## [35] "Ormosia_pinnata" "Oxytropis_kansuensis"
## [37] "Oxytropis_ochrocephala" "Pasania_sieboldiana"
## [39] "Pedicularis_kansuensis" "Phragmites_communis"
## [41] "Picea_asperata" "Pinus_tabulaeformis"
## [43] "Polygonatum_odoratum" "Potentilla_fruticosa"
## [45] "Potentilla_ragarioides" "Potentilla_tanacetifolia"
## [47] "Psychotria_rubra" "Quercus_mongolica"
## [49] "Randia_canthioides" "Rhododendron_ovatum"
## [51] "Rubus_chamaemorus" "Salix_phylicifolia"
## [53] "Schefflera_octophylla" "Schima_superba"
## [55] "Spiraea_salicifolia" "Stipa_krylovii"
## [57] "Symplocos_sumuntia" "Syzygium_bullockii"
## [59] "Syzygium_hancei" "Syzygium_levinei"
## [61] "Vaccinium_uliginosum"
## [1] " "
## [1] "Number of species:"
## [1] 61
## [1] " "
## [1] "Result of Phylogenetic Signal:"
## K PIC.variance.obs PIC.variance.rnd.mean PIC.variance.P
## 1 0.1494805 0.004331925 0.0171982 0.0376
## PIC.variance.Z
## 1 -0.7168398
## [1] " "
## [1] "Saving phylogenetic tree"

## [1] " "
## [1] "--------------End of this combination----------------"
## [1] " "
## [1] " "
## [1] "-------------Combination: 20 ---------------"
## dat.Treatment dat.Variable
## 1064 N addition NRE
## [1] " "
## [1] "List of species:"
## [1] "Agropyron_cristatum" "Andromeda_polifolia"
## [3] "Andropogon_gerardii" "Anemone_speciosa"
## [5] "Artemisia_frigida" "Artemisia_scoparia"
## [7] "Betula_nana" "Cannabis_sativa"
## [9] "Carex_caryophyllea" "Carex_sempervirens"
## [11] "Chamaedaphne_calyculata" "Chenopodium_acuminatum"
## [13] "Cleistogenes_songorica" "Cleistogenes_squarrosa"
## [15] "Convolvulus_ammannii" "Empetrum_hermaphroditum"
## [17] "Eriophorum_vaginatum" "Festuca_ovina"
## [19] "Helianthus_grosseserratus" "Kochia_prostrata"
## [21] "Melilotoides_ruthenica" "Panicum_virgatum"
## [23] "Phragmites_communis" "Polygonum_bistorta"
## [25] "Potentilla_tanacetifolia" "Rhododendron_groenlandicum"
## [27] "Rubus_chamaemorus" "Sorghastrum_nutans"
## [29] "Stipa_breviflora" "Stipa_krylovii"
## [31] "Tiarella_polyphylla" "Vaccinium_uliginosum"
## [1] " "
## [1] "Number of species:"
## [1] 32
## [1] " "
## [1] "Result of Phylogenetic Signal:"
## K PIC.variance.obs PIC.variance.rnd.mean PIC.variance.P
## 1 0.1170968 0.008491621 0.008886101 0.5927
## PIC.variance.Z
## 1 -0.08097604
## [1] " "
## [1] "Saving phylogenetic tree"

## [1] " "
## [1] "--------------End of this combination----------------"
## [1] " "
## [1] " "
## [1] "-------------Combination: 21 ---------------"
## dat.Treatment dat.Variable
## 1101 N addition PRE
## [1] " "
## [1] "List of species:"
## [1] "Agropyron_cristatum" "Andromeda_polifolia"
## [3] "Anemone_speciosa" "Artemisia_scoparia"
## [5] "Betula_nana" "Cannabis_sativa"
## [7] "Carex_caryophyllea" "Chamaedaphne_calyculata"
## [9] "Chenopodium_acuminatum" "Cleistogenes_squarrosa"
## [11] "Empetrum_hermaphroditum" "Eriophorum_vaginatum"
## [13] "Melilotoides_ruthenica" "Phragmites_communis"
## [15] "Potentilla_tanacetifolia" "Rhododendron_groenlandicum"
## [17] "Rubus_chamaemorus" "Stipa_krylovii"
## [19] "Vaccinium_uliginosum"
## [1] " "
## [1] "Number of species:"
## [1] 19
## [1] " "
## [1] "Result of Phylogenetic Signal:"
## K PIC.variance.obs PIC.variance.rnd.mean PIC.variance.P
## 1 0.2176153 0.007897228 0.005137034 0.9128
## PIC.variance.Z
## 1 1.381649
## [1] " "
## [1] "Saving phylogenetic tree"

## [1] " "
## [1] "--------------End of this combination----------------"
## [1] " "
## [1] " "
## [1] "-------------Combination: 22 ---------------"
## dat.Treatment dat.Variable
## 1121 eCO2 foliar C
## [1] " "
## [1] "List of species:"
## [1] "Acer_barbatum"
## [2] "Acer_rubrum"
## [3] "Betula_papyrifera"
## [4] "Cercis_canadensis"
## [5] "Eucalyptus_deglupta_Eucalyptus_camaldulensis"
## [6] "Eucalyptus_robusta"
## [7] "Eucalyptus_tereticornis"
## [8] "Eucalyptus_urophylla"
## [9] "Galactia_elliottii"
## [10] "Glycine_max"
## [11] "Gossypium_hirsutum"
## [12] "Hydrangea_paniculata"
## [13] "Liquidambar_styraciflua"
## [14] "Liriodendron_tulipifera"
## [15] "Phragmites_australis"
## [16] "Plantago_asiatica"
## [17] "Polygonum_sachalinense"
## [18] "Populus_pseudo-simonii"
## [19] "Populus_tremuloides"
## [20] "Prunus_serotina"
## [21] "Quercus__phellos"
## [22] "Quercus_alba"
## [23] "Quercus_chapmanii"
## [24] "Quercus_geminata"
## [25] "Quercus_myrtifolia"
## [26] "Quercus_rubra"
## [27] "Quercus_velutina"
## [28] "Robinia_pseudo-acacia"
## [29] "Sasa_kurilensis"
## [30] "Tiarella_polyphylla"
## [31] "Ulmus_alata"
## [1] " "
## [1] "Number of species:"
## [1] 31
## [1] " "
## [1] "Result of Phylogenetic Signal:"
## K PIC.variance.obs PIC.variance.rnd.mean PIC.variance.P PIC.variance.Z
## 1 0.322698 0.0001742363 0.0003921904 0.5952 -0.452623
## [1] " "
## [1] "Saving phylogenetic tree"

## [1] " "
## [1] "--------------End of this combination----------------"
## [1] " "
## [1] " "
## [1] "-------------Combination: 23 ---------------"
## dat.Treatment dat.Variable
## 1160 eCO2 foliar N
##
## Multivariate Meta-Analysis Model (k = 106; method: REML)
##
## logLik Deviance AIC BIC AICc
## -98.1207 196.2414 200.2414 205.5493 200.3590
##
## Variance Components:
##
## estim sqrt nlvls fixed factor R
## sigma^2 0.0000 0.0000 60 no Species yes
##
## Test for Heterogeneity:
## Q(df = 105) = 3.2643, p-val = 1.0000
##
## Model Results:
##
## estimate se zval pval ci.lb ci.ub
## -0.0886 0.0971 -0.9118 0.3619 -0.2789 0.1018
##
## ---
## Signif. codes: 0 '***' 0.001 '**' 0.01 '*' 0.05 '.' 0.1 ' ' 1
##
## [1] " "
## [1] "List of species:"
## [1] "Acer_barbatum"
## [2] "Acer_campestre"
## [3] "Acer_rubrum"
## [4] "Acer_saccharum"
## [5] "Achillea_millefolium"
## [6] "Acmena_acuminatissima"
## [7] "Agropyron_repens"
## [8] "Alphitonia_petriei"
## [9] "Anemone_cylindrica"
## [10] "Betula_papyrifera"
## [11] "Bromus_inermis"
## [12] "Caragana_microphylla"
## [13] "Carpinus_betulus"
## [14] "Castanopsis_hystrix"
## [15] "Cercis_canadensis"
## [16] "Cornus_florida"
## [17] "Eucalyptus_deglupta_Eucalyptus_camaldulensis"
## [18] "Eucalyptus_robusta"
## [19] "Eucalyptus_tereticornis"
## [20] "Eucalyptus_urophylla"
## [21] "Fagus_sylvatica"
## [22] "Flindersia_brayleyana"
## [23] "Galactia_elliottii"
## [24] "Glycine_max"
## [25] "Gossypium_hirsutum"
## [26] "Hydrangea_paniculata"
## [27] "Koeleria_cristata"
## [28] "Leymus_chinensis"
## [29] "Liquidambar_styraciflua"
## [30] "Liriodendron_tulipifera"
## [31] "Ormosia_pinnata"
## [32] "Phragmites_australis"
## [33] "Picea_abies"
## [34] "Pinus_taeda"
## [35] "Plantago_asiatica"
## [36] "Poa_pratensis"
## [37] "Polygonum_sachalinense"
## [38] "Populus_nigra"
## [39] "Populus_pseudo-simonii"
## [40] "Populus_tremuloides"
## [41] "Prunus_serotina"
## [42] "Quercus__phellos"
## [43] "Quercus_alba"
## [44] "Quercus_chapmanii"
## [45] "Quercus_geminata"
## [46] "Quercus_myrtifolia"
## [47] "Quercus_petraea"
## [48] "Quercus_rubra"
## [49] "Quercus_velutina"
## [50] "Robinia_pseudo-acacia"
## [51] "Salix_myrsinifolia"
## [52] "Sasa_kurilensis"
## [53] "Schima_superba"
## [54] "Solidago_rigida"
## [55] "Syzygium_hancei"
## [56] "Tiarella_polyphylla"
## [57] "Tilia_platyphyllos"
## [58] "Ulmus_alata"
## [59] "Vaccinium_myrtillus"
## [60] "Vaccinium_vitis-idaea"
## [1] " "
## [1] "Number of species:"
## [1] 60
## [1] " "
## [1] "Result of Phylogenetic Signal:"
## K PIC.variance.obs PIC.variance.rnd.mean PIC.variance.P
## 1 0.1666999 0.001027199 0.001879225 0.0801
## PIC.variance.Z
## 1 -1.05286
## [1] " "
## [1] "Saving phylogenetic tree"

## [1] " "
## [1] "--------------End of this combination----------------"
## [1] " "
## [1] " "
## [1] "-------------Combination: 24 ---------------"
## dat.Treatment dat.Variable
## 1267 eCO2 foliar P
## [1] " "
## [1] "List of species:"
## [1] "Acer_rubrum" "Acmena_acuminatissima"
## [3] "Alphitonia_petriei" "Castanopsis_hystrix"
## [5] "Cercis_canadensis" "Cornus_florida"
## [7] "Eucalyptus_robusta" "Eucalyptus_tereticornis"
## [9] "Flindersia_brayleyana" "Liquidambar_styraciflua"
## [11] "Ormosia_pinnata" "Pinus_taeda"
## [13] "Schima_superba" "Syzygium_hancei"
## [15] "Vaccinium_myrtillus" "Vaccinium_vitis-idaea"
## [1] " "
## [1] "Number of species:"
## [1] 16
## [1] " "
## [1] "Result of Phylogenetic Signal:"
## K PIC.variance.obs PIC.variance.rnd.mean PIC.variance.P
## 1 0.2414633 0.0007827651 0.0007516691 0.6888
## PIC.variance.Z
## 1 0.07174423
## [1] " "
## [1] "Saving phylogenetic tree"

## [1] " "
## [1] "--------------End of this combination----------------"
## [1] " "
## [1] " "
## [1] "-------------Combination: 25 ---------------"
## dat.Treatment dat.Variable
## 1296 eCO2 foliar CN
##
## Multivariate Meta-Analysis Model (k = 38; method: REML)
##
## logLik Deviance AIC BIC AICc
## -34.4036 68.8071 72.8071 76.0290 73.1601
##
## Variance Components:
##
## estim sqrt nlvls fixed factor R
## sigma^2 0.0000 0.0000 31 no Species yes
##
## Test for Heterogeneity:
## Q(df = 37) = 0.8057, p-val = 1.0000
##
## Model Results:
##
## estimate se zval pval ci.lb ci.ub
## 0.1061 0.1622 0.6538 0.5132 -0.2119 0.4240
##
## ---
## Signif. codes: 0 '***' 0.001 '**' 0.01 '*' 0.05 '.' 0.1 ' ' 1
##
## [1] " "
## [1] "List of species:"
## [1] "Acer_barbatum"
## [2] "Acer_rubrum"
## [3] "Betula_papyrifera"
## [4] "Cercis_canadensis"
## [5] "Eucalyptus_deglupta_Eucalyptus_camaldulensis"
## [6] "Eucalyptus_robusta"
## [7] "Eucalyptus_tereticornis"
## [8] "Eucalyptus_urophylla"
## [9] "Galactia_elliottii"
## [10] "Glycine_max"
## [11] "Gossypium_hirsutum"
## [12] "Hydrangea_paniculata"
## [13] "Liquidambar_styraciflua"
## [14] "Liriodendron_tulipifera"
## [15] "Phragmites_australis"
## [16] "Plantago_asiatica"
## [17] "Polygonum_sachalinense"
## [18] "Populus_pseudo-simonii"
## [19] "Populus_tremuloides"
## [20] "Prunus_serotina"
## [21] "Quercus__phellos"
## [22] "Quercus_alba"
## [23] "Quercus_chapmanii"
## [24] "Quercus_geminata"
## [25] "Quercus_myrtifolia"
## [26] "Quercus_rubra"
## [27] "Quercus_velutina"
## [28] "Robinia_pseudo-acacia"
## [29] "Sasa_kurilensis"
## [30] "Tiarella_polyphylla"
## [31] "Ulmus_alata"
## [1] " "
## [1] "Number of species:"
## [1] 31
## [1] " "
## [1] "Result of Phylogenetic Signal:"
## K PIC.variance.obs PIC.variance.rnd.mean PIC.variance.P
## 1 0.6417335 0.0002757535 0.001236894 0.0059
## PIC.variance.Z
## 1 -1.073798
## [1] " "
## [1] "Saving phylogenetic tree"

## [1] " "
## [1] "--------------End of this combination----------------"
## [1] " "
## [1] " "
## [1] "-------------Combination: 26 ---------------"
## dat.Treatment dat.Variable
## 1335 eCO2 foliar NP
## [1] " "
## [1] "List of species:"
## [1] "Acer_rubrum" "Acmena_acuminatissima"
## [3] "Alphitonia_petriei" "Castanopsis_hystrix"
## [5] "Cercis_canadensis" "Cornus_florida"
## [7] "Eucalyptus_robusta" "Eucalyptus_tereticornis"
## [9] "Flindersia_brayleyana" "Liquidambar_styraciflua"
## [11] "Ormosia_pinnata" "Pinus_taeda"
## [13] "Schima_superba" "Syzygium_hancei"
## [15] "Vaccinium_myrtillus" "Vaccinium_vitis-idaea"
## [1] " "
## [1] "Number of species:"
## [1] 16
## [1] " "
## [1] "Result of Phylogenetic Signal:"
## K PIC.variance.obs PIC.variance.rnd.mean PIC.variance.P
## 1 0.5117789 0.0003207588 0.0006629795 0.1415
## PIC.variance.Z
## 1 -0.6431006
## [1] " "
## [1] "Saving phylogenetic tree"

## [1] " "
## [1] "--------------End of this combination----------------"
## [1] " "
## [1] " "
## [1] "-------------Combination: 27 ---------------"
## dat.Treatment dat.Variable
## 1364 eCO2 NRE
## [1] " "
## [1] "List of species:"
## [1] "Acer_rubrum" "Acer_saccharum"
## [3] "Ambrosia_dumosa" "Betula_papyrifera"
## [5] "Cercis_canadensis" "Cornus_florida"
## [7] "Larrea_tridentata" "Liquidambar_styraciflua"
## [9] "Lycium_pallidum" "Phalaris_aquatica"
## [11] "Pinus_taeda" "Populus_alba"
## [13] "Populus_euramerica" "Populus_nigra"
## [15] "Populus_tremuloides" "Quercus_alba"
## [1] " "
## [1] "Number of species:"
## [1] 16
## [1] " "
## [1] "Result of Phylogenetic Signal:"
## K PIC.variance.obs PIC.variance.rnd.mean PIC.variance.P
## 1 0.1616517 0.0006514611 0.001542177 0.2563
## PIC.variance.Z
## 1 -0.7815919
## [1] " "
## [1] "Saving phylogenetic tree"

## [1] " "
## [1] "--------------End of this combination----------------"
## [1] " "
## [1] " "
## [1] "-------------Combination: 28 ---------------"
## dat.Treatment dat.Variable
## 1384 eCO2 PRE
## [1] " "
## [1] "List of species:"
## [1] "Acer_rubrum" "Ambrosia_dumosa"
## [3] "Cercis_canadensis" "Cornus_florida"
## [5] "Liquidambar_styraciflua" "Lycium_pallidum"
## [7] "Pinus_taeda"
## [1] " "
## [1] "Number of species:"
## [1] 7
## [1] " "
## [1] "Result of Phylogenetic Signal:"
## K PIC.variance.obs PIC.variance.rnd.mean PIC.variance.P
## 1 0.6060295 0.0002491684 0.0002470403 0.3597
## PIC.variance.Z
## 1 0.05316673
## [1] " "
## [1] "Saving phylogenetic tree"

## [1] " "
## [1] "--------------End of this combination----------------"
## [1] " "
## [1] " "

#### Warming foliar NP ##################
#### Increased precipitation foliar N ###################
#### N addition foliar NP ###################
#### eCO2 foliar CN ###################
